# Endothelial Cell Protein Targeting by Myeloperoxidase-Derived 2-Chlorofatty Aldehyde

**DOI:** 10.3390/antiox11050940

**Published:** 2022-05-10

**Authors:** Shubha Shakya, Roger A. Herr, Haley L. Carlson, Raphael A. Zoeller, Carolyn J. Albert, David A. Ford

**Affiliations:** 1 Center for Cardiovascular Research, Saint Louis University School of Medicine, St. Louis, MO 63104, USA; shubha.shakya@slu.edu (S.S.); r.a.herr@gmail.com (R.A.H.); haley.carlson@slu.edu (H.L.C.); carolyn.albert@health.slu.edu (C.J.A.); 2 Edward A. Doisy Department of Biochemistry and Molecular Biology, Saint Louis University School of Medicine, St. Louis, MO 63104, USA; 3 Department of Physiology and Biophysics, Boston University School of Medicine, Boston, MA 02118, USA; rzoeller@bu.edu

**Keywords:** chlorinated lipids, protein modification, proteomics, myeloperoxidase

## Abstract

Neutrophils are important cellular mediators of injury and repair in diseases including ischemic heart disease, atherosclerosis, and sepsis. Myeloperoxidase-derived (MPO)-oxidants released from neutrophils are potential mediators of endothelial injury in disease. MPO-derived HOCl attacks plasmalogen phospholipid to liberate 2-chlorofatty aldehyde (2-ClFALD). Both 2-ClFALD and its oxidation product, 2-chlorofatty acid (2-ClFA), are electrophilic lipids, and both probably react with proteins through several mechanisms. In the present study, we investigate protein modification specifically by 2-ClFALD under non-reducing conditions (e.g., without stabilizing Schiff base bonds), which likely reflects nucleophilic targeting of the electrophilic chlorinated carbon. Protein modification by the ω-alkyne analog of 2-chlorohexadecanal (2-ClHDA), 2-ClHDyA, was compared to that with the ω-alkyne analog of 2-chlorohexadecanoic acid (2-ClHA), 2-ClHyA, in multiple cell lines, which demonstrated 2-ClFALD preferentially modifies proteins compared to 2-ClFA. The 2-ClHDyA modified proteins from EA.hy926 cells and human lung microvascular endothelial cells analyzed by shotgun proteomics and over-representation analysis included adherens junction, cell adhesion molecule binding, and cell substrate junction enrichment categories. It is possible that proteins in these groups may have roles in previously described 2-ClFALD-elicited endothelial barrier dysfunction.

## 1. Introduction

Neutrophils are the first responders of the immune system [1,2,3]. Neutrophils have been implicated in a number of diseases including ischemic heart disease, atherosclerosis, and sepsis [4,5,6]. The intimate relationship between circulating neutrophils and endothelial injury probably has a key role in the pathophysiological sequelae of these diseases. In this respect, the activation of neutrophils in the circulation, or as they tether to the endothelium, is accompanied by a number of reactive oxygen species (ROS) and proteases, which may impact endothelial function. We have shown neutrophil myeloperoxidase-derived HOCl targets both neutrophil and endothelial plasmalogens leading to the production of 2-chlorofatty aldehyde (2-ClFALD), and subsequently its oxidation product, 2-chlorofatty acid (2-ClFA) [7,8,9]. In addition to 2-ClFA, 2-chlorodicarboxylic acid ω-oxidation products of 2-ClFA have been identified, which are excreted in the urine [10]. The 2-ClFALD can also be reduced to 2-chlorofatty alcohol [7,8]. However, 2-chlorofatty alcohol has not been reported to be present in vivo. Plasmalogens are a major phospholipid in endothelial cells, monocytes, neutrophils, and other tissues in the human body [11,12,13]. Increased 2-ClFALD levels have been found in activated neutrophils [9], monocytes [14], human atherosclerotic tissue [15], and in infarcted rat heart tissue [16]. Furthermore, 2-ClFALD causes endothelial cell activation [17,18], blood–brain barrier dysfunction [19,20], and neutrophil chemoattraction [9]. The predominant molecular species of 2-ClFALD are 2-chlorohexadecanal (2-ClHDA) and 2-chlorooctadecanal, since their precursor aliphatic groups are present at the *sn*-1 position of plasmalogens. Additionally, recently it was revealed that the neutrophil plasmalogen pool has a very diverse group of molecular species including those with very long chain aliphatic groups that give rise to very long chain molecular species of 2-ClFALD [21].

Aldehydes are reactive with primary amines resulting in Schiff base adduct formation. The identification of Schiff base adducts is enhanced by stabilizing the adducts by reduction. We have previously shown that 2-ClHDA forms Schiff base adducts with amines present in peptides and phosphatidylethanolamine [22]. Furthermore, the proteome of brain endothelial cells modified by 2-ClHDA under reducing conditions has been described [19]. We have shown that the α-chlorinated carbon of 2-ClHDA undergoes nucleophilic attack by the cysteine sulfhydryl of glutathione [23], which suggests that 2-ClHDA may modify proteins through mechanisms independent of the Schiff base adduct formation (i.e., protein alkylation of nucleophilic residues). In addition, myeloperoxidase-generated 2-ClFALD may ultimately lead to protein modification following oxidation to 2-ClFA and subsequent thioesterification mechanisms similar to protein palmitoylation.

In the present study, we investigated protein modification by 2-ClHDA in the absence of reduction, the conversion of 2-ClHDA to its oxidation product, 2-chlorohexadecanoic acid (2-ClHA), as well as the relative amount of protein modification by 2-ClHA compared to 2-ClHDA. For these studies we employed the click chemistry analogs, ω-alkynes, of 2-ClHDA and 2-ClHA, to investigate protein modification in several cell types including fatty aldehyde dehydrogenase (FALDH)-competent CHO.K1 cells, FALDH-deficient CHO cells (FAA.K1A), transformed endothelial cells (EA.hy926), and primary human lung microvascular endothelial cells (HLMVECs).

## 2. Material and Methods

### 2.1. Materials

Cell culture supplies were purchased from Sigma-Aldrich (St. Louis, MO, USA). Click chemistry reagents were purchased from Click Chemistry Tools, (Scottsdale, AZ, USA). All other chemicals were purchased from Sigma-Aldrich (St. Louis, MO, USA) or Thermo Fisher Scientific (Waltham, MA, USA). The alkyne analog of 2-ClHDA (2-ClHDyA) and 2-ClHA (2-ClHyA), were synthesized, according to the protocols described previously [17,24,25].

### 2.2. Cell Culture

Wild-type Chinese hamster ovary (CHO.K1) cells and an isolated mutant cell line (FAA.K1A) with defective fatty aldehyde dehydrogenase (FALDH) activity were grown at 37 °C with Ham’s F-12 (Corning, cat.10-080-CV, Corning, NY, USA) medium with 10% fetal bovine serum and supplemented with 1 mM glutamine in 5% CO_2_/95% air [26,27]. CHO.K1 and FAA.K1A were provided by Raphael A. Zoeller, Boston University. EA.hy926 cells (ATCC cat. CRL-2922, Manassas, VA, USA) (Passage <8) were cultured in complete media, Dulbecco’s modified Eagle’s medium (DMEM, Sigma-Aldrich, St Louis, MO, USA), supplemented with 10% fetal bovine serum (FBS) at 37 °C in humidified atmosphere with 10% CO_2_ (*v*/*v*). Human lung microvascular endothelial cell (HLMVEC) (PromoCell cat. C-12281, Heidelberg, Germany) were grown in EGM-2MV (Lonza, cat. CC-3202, Basel, Switzerland) in 5% CO_2_/95% air at 37 °C. All experiments were carried out in the absence of serum.

### 2.3. Metabolism of 2-ClHDyA in Cells

CHO.K1, FAA.K1A, EA.hy926 and HLMVEC cells were grown to confluence in 6-well plates. Prior to treatments with click analogs of chlorolipids, the cell culture media was switched to fresh serum-free cell culture media. Cells were treated with 10 μM 2-ClHDyA. At the end of the treatments, cell and media lipids were extracted using the Bligh and Dyer method in the presence of 2-Cl-[*d*_4_]HDA and 2-Cl-[*d*_4_]HA [28]. The 2-ClHDyA was analyzed following derivatization to their pentafluorobenzyl (PFB) oximes using PFB hydroxylamine. The derivatized species were analyzed on GC/MS using selected ion monitoring, as previously described [29,30]. Free 2-ClHyA was analyzed by LC/MS (Altis, Thermo Fisher Scientific, Waltham, MA, USA) using selected reaction monitoring, as previously described [29,30].

### 2.4. Visualization of Cell Protein on Gel Electrophoresis

CHO.K1, FAA.K1A, EA.hy926, and HLMVEC cells were grown to confluence in 6-well plates. Cells were treated with either DMSO, 10 µM 2-ClHyA or 10 µM 2-ClHDyA for 1 h at 37 °C. After 1 h, cells were lysed with RIPA lysis buffer with 1XcOmplete mini EDTA-free protease inhibitor cocktail and 400 µM phenylmethylsulfonyl fluoride (PMSF) protease inhibitor. Cells were lysed and DNA sheared by passing through a 26G needle four–five times. Insoluble material in the lysates was removed by centrifuging at 14,000× *g* for 20 min at 4 °C. Protein concentration was determined using the BCA assay (Pierce cat. 23225, Thermo Fisher Scientific, Waltham, MA, USA). Protein lysates (100 µg) were conjugated to carboxytetramethylrhodamine (TAMRA)-azide as previously described using the Click-It daction buffer kit (Thermo Fisher Scientific, cat.10269, Waltham, MA, USA), following the manufacturer’s protocols [25]. Proteins were precipitated in methanol-chloroform. NuPAGE LDS Sample buffer (2X) (Invitrogen, cat. NP0007, Waltham, MA, USA) was added to the dry pellet at a concentration of 1–2 mg/mL. Samples were heated at 70 °C for 10 min and then cooled and centrifuged at 15,000× *g* for 5 min. Proteins were then resolved on 4–12% Bis-Tris gels by electrophoresis with MOPS running buffer. Modified proteins were subsequently visualized at 532/580 nm (excitation/emission) using a Typhoon imager. After visualization, the gel was stained with Coomassie blue for total protein using the SimplyBlue SafeStain (Invitrogen, Waltham, MA, USA).

### 2.5. Detection of 2-ClHDyA and 2-ClHyA in Cells

Confocal immunofluorescence experiments were performed, as previously described [17,25]. Briefly, cells were grown to confluence on sterile coverslips. Confluent cells were treated with DMSO, 10 µM 2-ClHDyA, or 10 µM 2-ClHyA, for 60 min. Cells were washed with PBS, fixed with formalin, and permeabilized with 0.25% Triton X-100 for 10 min. Unreacted lipids were removed by washing the cells with 2% (*w*/*v*) bovine serum albumin (BSA) in PBS and labeled with 5 µM TAMRA-azide. Excess reagents were washed with 2% BSA in PBS. The coverslips were mounted onto microscope slides with a vectashield solution containing 4,6-diamidino-2-phenylindole (DAPI; Vector Laboratories, Burlingame, CA, USA).

### 2.6. Confocal Microscopy

A Leica SP5 confocal microscope (Leica Microsystems, Mannheim, Germany) with a 63 × 1.4 oil immersion objective was used to acquire images. TAMRA-azide fluorescence was excited at 543 nm and detected between 570 and 650 nm. DAPI fluorescence was detected between 440 and 470 nm. The fluorescence was measured in 30 cells in five fields of view per condition. Total corrected cell fluorescence was calculated by using ImageJ software [25].

### 2.7. Click Reactions of 2-ClHDyA Modified Proteins with Biotin-Azide

EA.hy926 cells (Passage <8) were grown to confluence in T75 flasks with complete media Dulbecco’s Modified Eagle’s Medium (DMEM) supplemented with 10% FBS at 37 °C in humidified atmosphere with 10% CO_2_ (*v*/*v*). The cells were washed with 10 mL of DPBS (two times) followed by addition of 10 µM HDyA or 10 µM 2-ClHDyA or vehicle (DMSO only) in 11 mL DMEM for 1 h at 37 °C. After 1 h, cells were washed twice with cold DPBS, lysed with 750 µL RIPA with 1X cOmplete mini EDTA-free protease inhibitor cocktail and 400 µM PMSF. Protein lysates were incubated with high-capacity streptavidin-agarose beads (Pierce) equilibrated with lysis buffer (1X RIPA + 1X cOmplete mini EDTA-free protease inhibitor cocktail + 400 µM PMSF) overnight at 4 °C to reduce the background signal caused by endogenous biotinylated proteins. Protein concentrations of the supernatant were subsequently determined using the Pierce BCA Protein assay kit. Proteins from the supernatant (500 µg in 375 µL) were then diluted with 750 µL PBS. Subsequently, 100 µM of azide-biotin was added, followed by 90 µL of water, 40 µL of 150 µM THPTA, and 60 µL of a 1:5 ratio mixture of 150 mM THPTA: 30 mM Cu(II)SO_4_. The click reaction was then started by addition of 50 µL of 400 mM sodium ascorbate. Samples were subsequently mixed by end-to-end rotation for 30 min at room temperature. Excess click reagents were removed by using 10 mL Zeba 7K MW cutoff desalting columns (Thermo Fisher Scientific, cat. 89893, Waltham, MA, USA) equilibrated in DPBS, as per the manufacturer’s instructions. To prevent protein precipitation and degradation, NP-40 was added to a final concentration of 1%, SDS to final concentration 0.1%, PMSF to final concentration 400 µM and cOmplete mini protease inhibitor cocktail to a final concentration (1X).

### 2.8. Immunoprecipitation of Biotin-Azide Clicked Proteins with Streptavidin Beads

Biotin-azide clicked proteins were centrifuged (16,000× *g*_max_, 30 min, 4 °C) and supernatant was diluted to 2500 µL. A total of ~175 µg of cleaned biotin-azide clicked proteins were incubated with 30 µL of high-capacity streptavidin-agarose beads overnight at 4 °C with rotation. The beads were washed sequentially with 0.15% NP-40 (four times on ice), 6 M urea in 50 mM Tris pH 8.0 (4 times, 15 min, with rotation at 4 °C), 2 mM CaCl_2_ in 50 mM Tris pH 8.0 (4 times, 15 min on ice). Washed streptavidin beads were then subjected to either SDS-PAGE or proteomics analysis.

### 2.9. LC-MS/MS Analysis of Captured Proteins

Biotin-azide clicked proteins bound to streptavidin-agarose beads were subjected to on-bead trypsin digestion, peptide clean-up and proteomic analysis by LC-MS/MS. Additionally, control samples with beads alone were included and subjected to on-bead digest.

Briefly, proteins were reduced with 2.3 µL of 500 mM freshly made DTT at 56 °C for 30 min, followed by alkylation with 4.6 µL of 1M iodoacetamide (IAA) for 30 min in the dark. Excess IAA was quenched with an additional 2.4 µL of 500 mM DTT for 5 min at room temperature. Reduced and alkylated biotin-tagged proteins were digested with 500 ng of trypsin for 20 h at 37 °C by shaking at 1400 rpm. The tryptic peptides were cleaned up and concentrated with C18 columns (Thermo Fisher Scientific, cat. 89870, Waltham, MA, USA), according to the manufacturer’s protocol. Briefly, 225 µL of tryptic digest was mixed with 75 µL of 2% TFA in 20% acetonitrile. Tryptic peptides were eluted with 70% acetonitrile with 0.1% formic acid from C18 columns previously equilibrated with 0.5% TFA in 5% acetonitrile. The eluted peptides were dried and subsequently resuspended in 40 µL water/acetonitrile/formic acid (98/2/0.1).

Samples were analyzed on a Thermo Q Exactive orbitrap MS/MS equipped with a nanospray emitter (Thermo Fisher Scientific, Waltham, MA, USA). Nanospray parameters in positive ion mode were as follows: spray voltage, 2.0 kV; capillary temperature, 320 °C; S-lens RF level, 55.

Liquid chromatography was performed on a Dionex™ UltiMate™ 3000 RSLC (Thermo Fisher Scientific, Waltham, MA, USA). Mobile phase A consisted of 0.1% formic acid and mobile phase B consisted of 80% acetonitrile in water with 0.1% formic acid. Samples were loaded onto the loading column in 2% acetonitrile in water with 0.1% FA. The peptides were separated by reversed-phase chromatography on a C18 nano column (Acclaim PepMap C18 HPLC column 15 cm × 75 μm, 2 μm particles, 100 Å pore size, Thermo Fisher Scientific, Waltham, MA, USA) at a flow rate of 300 nL/min. The column temperature was maintained at 35 °C. The peptide samples were injected onto the column at 99% A, which was held for 3 min, followed by a linear gradient from 1% to 65% B over 90 min. Subsequently, hydrophobic peptides were step eluted over 1 min with 90% B, which was held for 4 min. Columns were subsequently re-equilibrated with 99% A for 22 min. The top 10 peptides were selected in a data-dependent mode for MS/MS fragmentation by collision-induced dissociation. The automatic gain control (AGC) target was set to 3 × 10^6^ with resolution 70,000 and maximum injection time 100 ms. For MS2 analysis, the AGC target mode was set to 5 × 10^4^ with a maximum injection time of 100 ms and a resolution of 17,500. The isolation window was set to 1.8 m/z and collision energy of 28 was used to fragment the ions. Dynamic exclusion of 40.0 s and charge exclusion of unassigned, 1, 6–8, and >8 were used.

### 2.10. Proteomic Analysis of Peptides of Captured Proteins

The MS/MS spectra were analyzed using Sequest HT and MSPepSearch, both integrated in Proteome Discoverer 2.4.1.15 (Thermo Fisher Scientific, Waltham, MA, USA). The Swiss-Prot (version: 25 October 2017, accessed 07 July 2020, St. Louis, MO, USA) database for Homo sapiens were used to search the data in Sequest HT. The NIST Human Orbitrap spectral library (NIST_Human_Orbitrap_HCD_20160923, version 1.0, accessed 07 September 2018, St. Louis, MO, USA) and ProteomeTools_HCD30_PD (version 1.0, accessed 16 October 2018, St. Louis, MO, USA) were used to search data with MSPepsearch. Search parameters for the Sequest HT database included trypsin, two missed cleavages, minimum peptide length of six, and maximum peptide length of 150. The dynamic modifications oxidation, N-terminal Acetyl, N-terminal Met-loss, and N-terminal Met-loss + Acetyl were allowed. Carbamidomethyl was specified as a static modification. The precursor mass tolerance was set to 10 ppm and fragment mass tolerance was set to 0.02 Da for both MSPepSearch and Sequest HT. Finally, the results were subjected to statistical analysis using the percolator node where the false discovery rate (FDR) was calculated using a decoy database search, and only high confidence peptide identifications with FDR < 0.01 were included. Identifications were accepted only for proteins with greater than one peptide identification and five PSMs. Further, accepted proteins that were present in seven of nine replicates were used for gene ontology analysis.

### 2.11. Gene Ontology Analysis

To functionally characterize the protein targets of 2-ClHDyA, we entered the UniProt accession ID of protein targets into the Web-based gene set analysis toolkit (WebGestalt 2019, accessed several times during December 2020 to May 2022) [31,32,33,34,35]. The latest version of the wGSEA 2019 recognizes 155,175 functional categories, 342 gene identifiers, and 12 organisms, including many user-defined functional databases [35]. We chose over-representation analysis as the enrichment method for gene ontology (Molecular Function (MF), Biological Process (BP), and Cellular Component (CC)). Homo sapiens was selected as the organism. The reference list was analyzed against the genome protein coding reference set. The parameters for the enrichment analysis for 2-ClHDyA-specific hits included the minimum number of IDs in the category (5), the maximum number of IDs in the category (2000), the Bonferroni method (*p* < 0.05) for computing the FDR (*p* < 0.05), and the significance level (FDR < 0.05). Protein analysis through evolutionary relations (Panther) was used to identify the protein classes [36,37].

### 2.12. SDS-PAGE and Western Blotting

Biotin-azide clicked proteins were eluted from streptavidin beads by heating the sample with 60 µL of NuPAGE LDS sample buffer at 96 °C for 10 min. The supernatant was mixed with NuPAGE reducing agent and heated at 70 °C for 10 min. The proteins were separated on Bis-Tris polyacrylamide gels (4–12%) using MOPS buffer over 50 min at 200 V. Proteins were visualized by silver staining. For Western blot analysis, 6 µg of protein was separated on Bis-Tris gels, and the proteins were transferred to Immobilon-P PVDF membranes (Millipore Sigma-Aldrich, St. Louis, MO, USA). Membranes were subsequently blocked with 5% BSA in PBS with Tween^®^20 (0.05% *v*/*v*) (PBS-T) overnight and then blotted with HRP-SA (100 ng/mL) for 1h. Following washing in PBS-T, biotin-tagged proteins were detected on CL-X Posure film (Thermo Fisher Scientific, cat. 34091, Waltham, MA, USA) using the ECL (Thermo Fisher Scientific, cat. 32106, Waltham, MA, USA).

## 3. Results

### 3.1. ClHDyA Metabolism and Protein Modification

**Figure 1:**
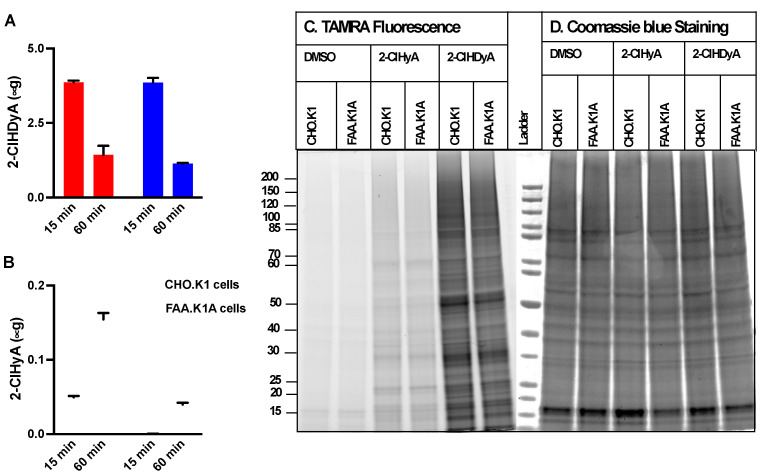
**2-ClHDyA metabolism and protein modification in CHO.K1 and FAA.K1A cells.** CHO.K1 and FAA.K1A cell lines were treated with 10 μM 2-ClHDyA for indicated time intervals. At the end of each time point, cellular and media lipids were extracted together, and 2-ClHDyA (**A**) and 2-ClHyA (**B**) were quantitated as described in Section 2. Values are average + SEM for *n* = 3 in both (**A**,**B**); (**C**) CHO.K1 and FAA.K1A cells were treated with either DMSO, 10 μM 2-ClHyA, or 10 μM 2-ClHDyA for 1 h at 37 °C. Proteins modified by these ω-alkyne chlorolipids were clicked with TAMRA-azide and were visualized after gel electrophoresis; (**D**) Coomassie blue staining of gel protein loads used for TAMRA fluorescence analyses.

**Figure 2:**
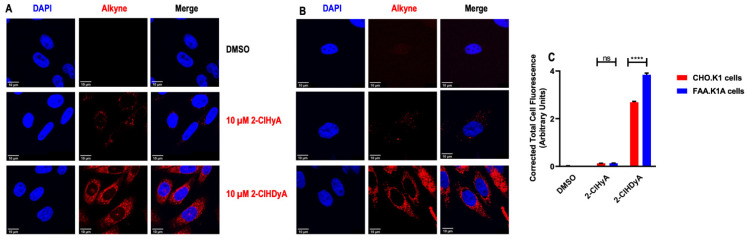
**Confocal imaging of proteins modified by 2-ClHDyA and 2-ClHyA in CHO.K1 (A) and FAA.K1A (B) cells.** CHO.K1 and FAA.K1A treated with 2-ClHDyA and 2-ClHyA as described in Figure 1 were subjected to confocal microscopy as described in Section 2. TAMRA in red indicates localization of proteins modified by 2-ClHDyA and 2-ClHyA and blue is DAPI nucleus staining; (**C**) Total corrected cell fluorescence was measured using ImageJ from CHO.K1 and FAA.K1A cells treated with DMSO, 10 µM 2-ClHDyA or 10 µM ClHyA following 1 h incubation. Mean + SEM **** *p* < 0.0001 and not significance (ns) for comparison within 2-ClHDyA and 2-ClHyA treatment between CHO.K1 and FAA.K1A cells, respectively.

The 2-ClFALD is metabolized to 2-ClFA in FALDH dependent manner [7,8,15]. Alternatively, 2-ClFALD has previously been shown to modify proteins [19,24,38]. Since the contribution of 2-ClFALD metabolism to 2-ClFA and subsequent protein modification by 2-ClFA has not been explored [19,24], we examined this possibility in four different cell lines. Initial studies compared CHO.K1 cells to FALDH deficient cells to show a defect in conversion of 2-ClFALD to 2-ClFA and to compare protein modification in these two cell lines. Data in Figure 1A show a similar loss of the click analog of 2-ClHDA, 2-ClHDyA over time in these cells. However, the appearance of the click analog of 2-ClHA, 2-ClHyA, was decreased in FALDH-deficient FAA.K1A cells (Figure 1B). Cells were treated with 10 μM 2-ClHDyA in these studies. This concentration has previously been shown to be physiologically relevant [9] and in other cell culture systems 10 μM 2-ClHDyA was not the cause of cell death following 1 h incubations [17,19,39]. Robust protein modification by 2-ClHDyA was observed in both CHO.K1 and FAA.K1A cells (Figure 1C). In comparison, 2-ClHyA protein modification was significantly less than that by 2-ClHDyA. Furthermore, confocal fluorescence imaging demonstrated greater protein modification of both CHO.K1 and FAA.K1A cells by 2-ClHDyA compared to 2-ClHyA (Figure 2A,B). Although co-localization studies were not performed, these results indicate disparate subcellular distributions of 2-ClHDyA and 2-ClHyA in CHO.K1 and FAA.K1A cells, which is similar to our previous findings for their distributions in endothelial cells [17,25]. Fluorescent intensity from confocal images also indicated 2-ClHDyA shows significantly more protein modification in FAA.K1A cells in comparison to CHO.K1 cells, which may be due to less 2-ClHDyA being converted to 2-ClHyA in FAA.K1A cells (Figure 2C).

**Figure 3:**
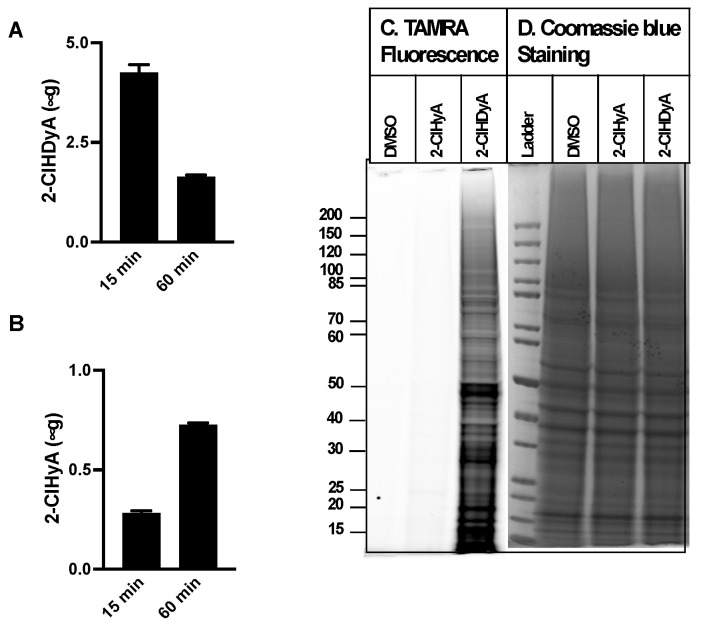
**2-ClHDyA metabolism and protein modification in EA.hy926 endothelial cells.** EA.hy926 cells were treated with 10 μM 2-ClHDyA for indicated time intervals. At the end of each time point, cellular and media lipids were extracted together, and 2-ClHDyA (**A**) and 2-ClHyA (**B**) were quantitated as described in Section 2. Values are average + SEM for *n* = 4 in both (**A**,**B**); (**C**) EA.hy926 cells were treated with either DMSO, 10 μM 2-ClHyA, or 10 μM 2-ClHDyA for 1 h at 37 °C. Proteins modified by these ω-alkyne chlorolipids were clicked with TAMRA-azide and were visualized after gel electrophoresis; (**D**) Coomassie blue staining of gel protein loads used for TAMRA fluorescence analyses.

**Figure 4:**
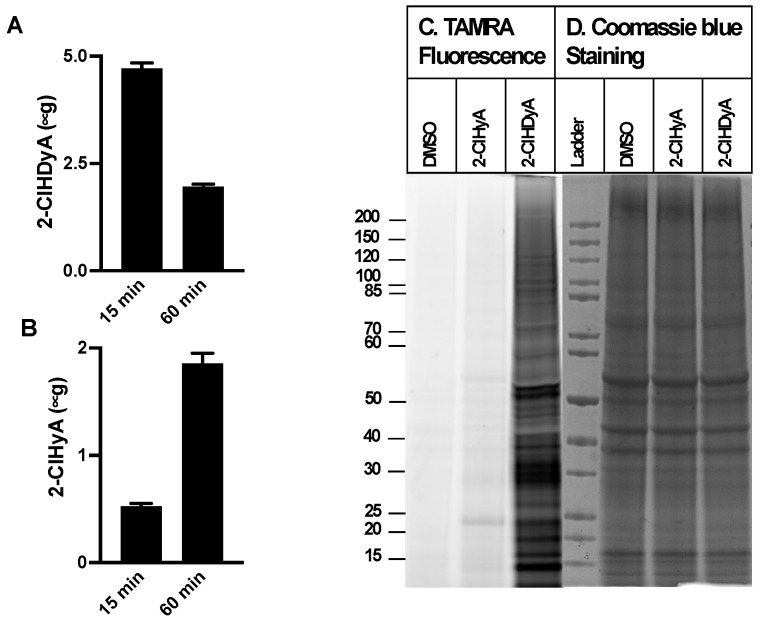
**2-ClHDyA metabolism and protein modification in HLMVECs.** HLMVECs were treated with 10 μM 2-ClHDyA for indicated time intervals. At the end of each time point, cellular and media lipids were extracted together, and 2-ClHDyA (**A**) and 2-ClHyA (**B**) were quantitated as described in Section 2. Values are average + SEM for *n* = 4 in both (**A**,**B**); (**C**) HLMVECs were treated with either DMSO, 10 μM 2-ClHyA, or 10 μM 2-ClHDyA for 1 h at 37 °C. Proteins modified by these ω-alkyne chlorolipids were clicked with TAMRA-azide and were visualized after gel electrophoresis; (**D**) Coomassie blue staining of gel protein loads used for TAMRA fluorescence analyses.

Since neutrophil activation leads to 2-ClFALD production in nearby endothelial cells [9], similar studies were performed with EA.hy926 cells, a transformed human endothelial model cell line, and HLMVECs, primary human lung microvascular endothelial cells. The EA.hy926 cell line is the result of fusing human umbilical vein endothelial cells with a thioguanine-resistant clone of A549 cells. EA.hy926 cells and HLMVEC metabolized 2-ClHDyA to 2-ClHyA more efficiently in comparison to the CHO.K1 and FAA.K1A cells (Figure 3A,B and Figure 4A,B). In addition, HLMVEC converted 2-ClHDyA to 2-ClHyA to a greater extent than the EA.hy926 cells. Despite this more efficient conversion of 2-ClHDyA to 2-ClHyA, 2-ClHDyA is likely still responsible for most protein modifications under the conditions employed, since the data in Figure 3C and Figure 4C show that there is minimal protein modification by 2-ClHyA in comparison to that by 2-ClHDyA.

### 3.2. Protein Targets of 2-ClHDyA in EA.hy926 Cells

**Figure 5:**
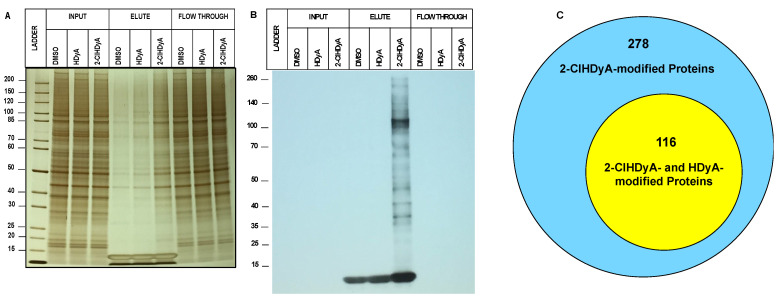
**2-ClHDyA modified EA.hy926 cell proteins.** Silver-stained gel (**A**) and Western blot (**B**) probed with streptavidin HRP of the samples from A were prepared as described in Section 2. Protein loads showing total proteins from lysates from each indicated condition (DMSO vehicle, HDyA (non-chlorinated aldehyde control) and 2-ClHDyA) input onto beads, elute from beads, and supernatant (Flow Through); (**C**) Venn diagram of number of 2-ClHDyA- and HDyA-modified proteins in EA.hy926 cells.

Our workflow to identify 2-ClHDA-modified proteins is shown in Figure S1. We treated EA.hy926 cells with either 2-ClHDyA, HDyA, or vehicle (DMSO) for 1 h at 37 °C (*n* = 3). Following streptavidin-agarose bead enrichment of modified proteins, eluted biotinylated proteins were separated and visualized in SDS-PAGE using silver stain (Figure 5A). The proteins were also transferred onto PVDF membranes and probed with streptavidin-HRP. Figure 5A,B shows successful protein pull-down of 2-ClHDyA-modified proteins by silver staining as well as the specificity of their biotinylation from click reactions by probing with streptavidin-HRP. Peptides generated by on-bead digest were analyzed by LC-MS/MS. The peptides associated with specific proteins were identified with Proteome Discoverer 2.4.1.15, using the Sequest HT and MSPepSearch databases. Only matches that met the defined criteria, as described in Section 2, were accepted. The samples were prepared with three biological replicates and analyzed three times (three technical replicates), each with LC-MS/MS.

**Figure 6:**
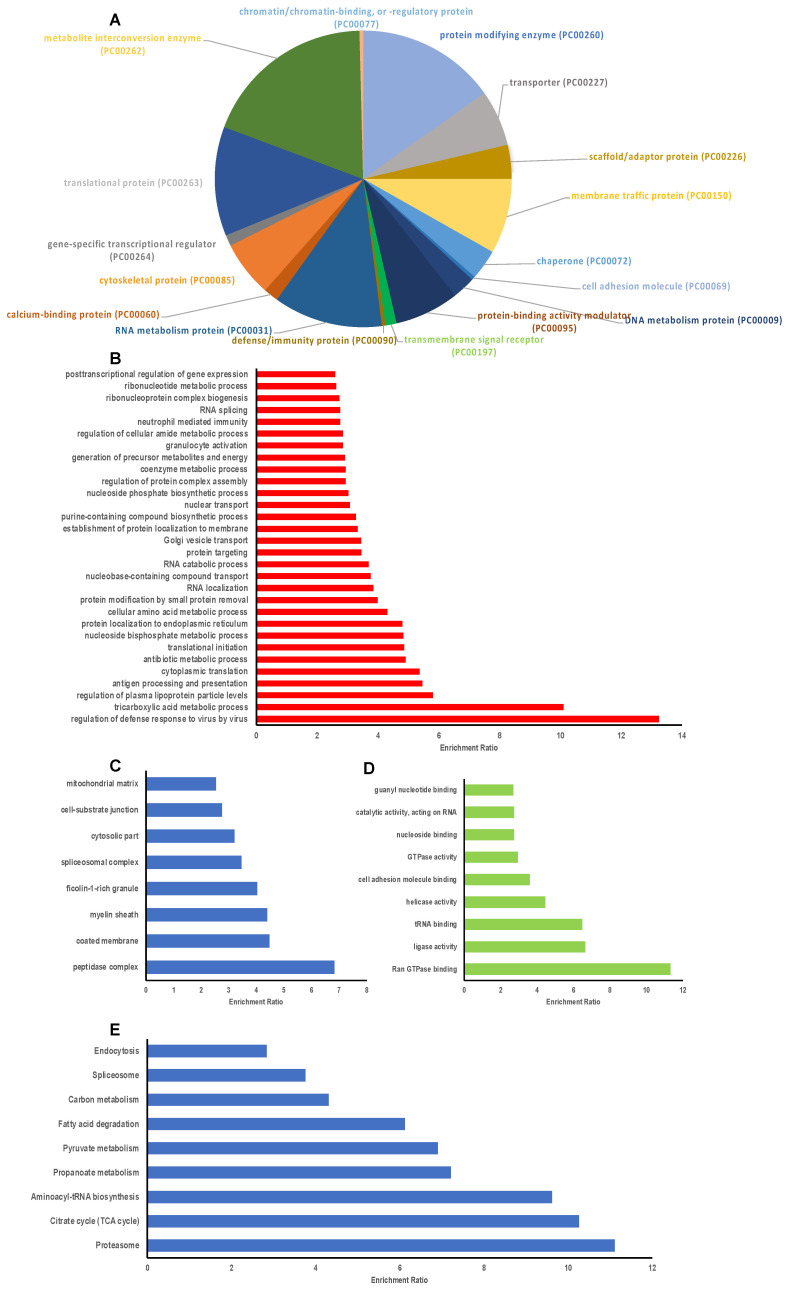
**Bioinformatic characterization of EA.hy926 proteins modified by 2-ClHDyA.** (**A**) Distribution of 2-ClHDyA-modified proteins by protein class analyzed using Panther; (**B**–**E**) Over representation analysis using the Web-based Gene Set Analysis Toolkit (WebGestalt). Enriched categories in Gene ontology (GO) Biological function (**B**); Cellular component (**C**); Molecular function (**D**); and KEGG Pathway using Bonferroni *p* < 0.05 for computing FDR (*p* < 0.05) (**E**).

A total of 394 proteins were modified by 2-ClHDyA (Figure 5C). Among these 394 proteins, 278 proteins were specifically modified by 2-ClHDyA samples. These 278 proteins were entered into the Panther database to identify the classes of protein targets (Figure 6A). Major classes of proteins targeted by 2-ClHDyA and HDyA included metabolite interconversion enzymes (21%), translational proteins (14.6%), protein modifying enzymes (12.7%), and nucleic acid metabolism proteins (11.4%). The identified proteins were also subjected to over-representation analysis (ORA) using WebGestalt. Protein user IDs were unambiguously mapped to unique Entrez Gene IDs. Data in Figure 6B–D show the extensive number of Entrez IDs associated with biological processes, cellular components, and molecular functions, respectively, which were significantly (FDR < 0.05) enriched, as determined by ORA. Additionally, the KEGG pathways for proteosome, aminoacyl-tRNA biosynthesis, citrate cycle, carbon metabolism, ribosome, spliceosome, and endocytosis were enriched (Figure 6E). A complete list of the EA.hy926 proteins modified by 2-ClHDyA is in Table S1. Since some biological processes elicited by 2-ClHDA may also be mediated by proteins modified by both 2-ClHDyA and HDyA, we also performed bioinformatics analyses on these proteins (Figure S2). A list of these proteins is in Table S2.

### 3.3. Protein Targets of 2-ClHDyA in Human Lung Microvascular Endothelial Cells

**Figure 7:**
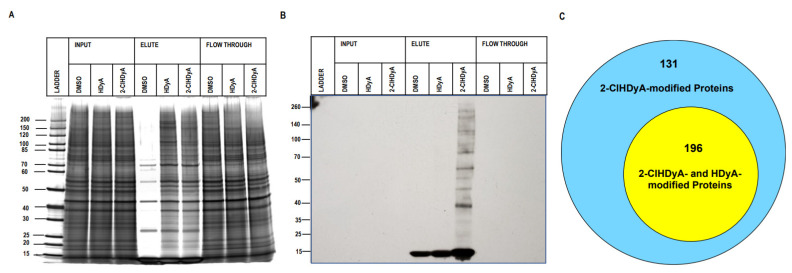
**2-ClHDyA modified HLMVEC cell proteins.** Silver-stained gel (**A**) and Western blot (**B**) probed with streptavidin HRP of the samples from A were prepared, as described in Section 2. Protein loads showing total proteins from lysates from each indicated condition (DMSO vehicle, HDyA (non-chlorinated aldehyde control), and 2-ClHDyA) input onto beads, elute from beads, and supernatant (Flow Through); (**C**) Venn diagram of number of 2-ClHDyA- and HDyA-modified proteins in HLMVECs.

**Figure 8:**
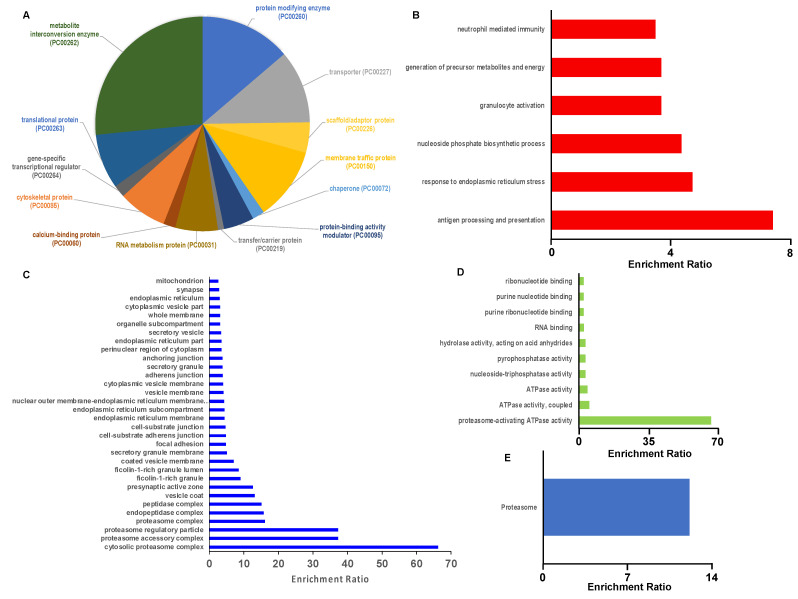
**Bioinformatic characterization of HLMVEC proteins modified by 2-ClHDyA.** (**A**) Distribution of 2-ClHDyA-modified proteins by protein class analyzed using Panther; (**B**–**E**) Over representation analysis using the Web-based Gene Set Analysis Toolkit (WebGestalt). Enriched categories in Gene ontology (GO) Biological function (**B**); Cellular component (**C**); Molecular function (**D**); and KEGG Pathway using Bonferroni *p* < 0.05 for computing FDR (*p* < 0.05) (**E**).

Since EA.hy926 cells are a transformed endothelial cell line that may have quite different properties compared to human primary endothelial cells, we also investigated the 2-ClHDyA-modified proteome in primary HLMVECs, using the same workflow employed for EA.hy926 proteomics studies. Figure 7A,B show the successful protein pull-down of 2-ClHDyA-modified proteins. A total of 327 proteins were modified by 2-ClHDyA in at least seven out of nine 2-ClHDyA replicates (Figure 7C). A complete list of the HLMVEC proteins modified by 2-ClHDyA is in Table S3. Of these 327 proteins, 196 of the proteins were also present in HDyA samples. The Panther database was used to identify the protein class distribution of the 327 proteins found in 2-ClHDyA samples. The major classes of proteins modified by 2-ClHDyA include the metabolite interconversion (23.2%), protein modifying enzyme (12.1%), transporter (10.4%), membrane traffic protein (10.0%), and translational (10.0%) classes, along with several other classes shown in Figure 8A. The list of 327 proteins modified by 2-ClHDyA was then entered into WebGestalt to identify significantly enriched gene ontology (GO) categories via ORA. There were numerous significantly enriched biological processes (FDR < 0.05) identified, as shown in Figure 8B. Since 2-ClHDA is produced during the oxidative burst of neutrophils it is interesting that neutrophil-mediated immunity and cell redox homeostasis are enriched biological processes. The neutrophil-mediated immunity group and cell redox homeostasis proteins are listed in Table S4 and Table S5, respectively. Significantly enriched (FDR < 0.05) cellular components and molecular functions for 2-ClHDyA-modified proteins are shown in Figure 8C,D. Finally, the significantly enriched KEGG pathways for this group of proteins are shown in Figure 8E, which show enrichment in the proteasome pathway, which may be important in the endothelial barrier dysfunction that has been observed in endothelial cells treated with 2-ClHDA [17]. Additionally, the individual adherens junction-associated proteins are listed in Table S6. Bioinformatics analyses on 2-ClHDyA modified proteins that were also modified by HDyA are shown in Figure S3 and listed in Table S7.

### 3.4. Comparison of Protein Targets of 2-ClHDyA in EA.hy196 Cells and Human Lung Microvascular Endothelial Cells

**Figure 9:**
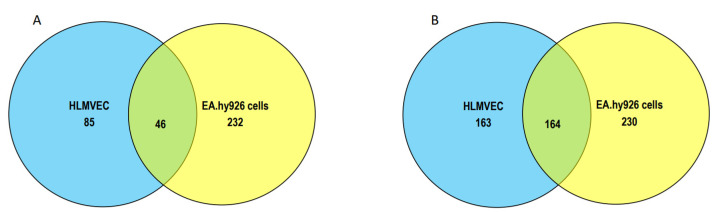
**Shared and unique proteins in EA.hy926 cells and HLMVECs modified by 2-ClHDyA.** (**A**) Venn diagram of number of proteins specifically modified by 2-ClHDyA in EA.hy926 cells and HLMVECs; (**B**) Venn diagram of number of proteins modified by 2-ClHDyA including those modified by HDyA in EA.hy926 cells and HLMVECs.

In comparing the proteins modified specifically by 2-ClHDyA in EA.hy196 cells and HLMVECs, there were 46 shared proteins (Figure 9A). The number of shared proteins was much greater in comparison to the proteins modified by 2-ClHDyA, which were also modified by HDyA (Figure 9B). The individual modified proteins shared between these cells under the criteria of data shown in Figure 9A,B are listed in Tables S8 and S9.

## 4. Discussion

The production of 2-ClFALD occurs during neutrophil activation due to MPO-derived hypochlorous acid targeting plasmalogens [9]. Then, 2-ClFALD can be subsequently oxidized to 2-ClFA [8]. Both 2-ClFALD and 2-ClFA are electrophilic lipids which can modify proteins. It is likely that 2-ClFA modifies proteins predominantly through thioesterification, but may also modify proteins by an alkylation reaction similar to that shown by 2-BrFA [40]. The 2-ClFALD modifies proteins conventionally through Schiff base adduct formation [19,22], which is stabilized under reducing conditions. However, protein alkylation by 2-ClFALD is a likely mechanism for 2-ClFALD modification of proteins, as supported by 2-ClFALD’s rapid modification of glutathione at the nucleophilic cysteine [23]. The present studies focus on protein modification by 2-ClFALD through alkylation reactions using a click chemistry analog of 2-ClFALD and click tools including TAMRA-azide and biotin-azide. These studies were performed in the absence of reducing conditions. Direct comparisons of 2-ClFALD protein modification to 2-ClFA protein modification indicated that the proteins modified by addition of 2-ClFALD likely were not a result of 2-ClFALD conversion to 2-ClFA and subsequent protein modification by the metabolite, 2-ClFA. These conclusions are drawn from experiments in CHO.K1 cells, FAA.K1A cells, EA.hy926 cells, and HLMVEC. The conversion of 2-ClFALD to 2-ClFA varied between the cell lines. Since FAA.K1A cells are deficient in FALDH, they displayed minimal conversion of 2-ClFALD to 2-ClFA compared to their wild-type cell CHO.K1. The transformed endothelial cells, EA.hy926 cells, converted 2-ClFALD to 2-ClFA more rapidly than CHO.K1 cells. Interestingly, the primary endothelial cells, HLMVEC, converted about 33% of the 2-ClFALD to 2-ClFA. Despite these variable conversions of 2-ClFALD to 2-ClFA in these cells, 2-ClFALD modification of proteins was both uniformly and significantly greater than 2-ClFA modification of proteins under all conditions.

Previous studies identifying protein targets of 2-ClHDA employed reducing conditions to stabilize the lipid–protein bond [19,38]. Nusshold, C. et al. identified 134 2-ClHDA modified proteins in hCMEC/D3 (an immortalized endothelial cell line), in which protein lysates were subjected to sodium cyanoborohydride reduction to stabilize Schiff base adducts [19]. Since we previously demonstrated the rapid reaction of 2-ClHDA with glutathione by an alkylation reaction [23], we examined proteins without using reducing conditions to evaluate protein modification that would be predominantly mediated via alkylation reactions. A workflow was developed for these studies utilizing the click alkyne analog of 2-ClHDA, 2-ClHDyA, which was then reacted with biotin-azide. We then applied affinity-based pull-down of the proteins using streptavidin beads to isolate the proteins modified by the 2-ClFALD analog. We chose an affinity-based approach to detect low-abundance proteins and remove unmodified proteins from the sample [19]. However, affinity-based methods can result in non-specific binding [41]. Therefore, we validated the affinity pull-down method by performing an on-bead digest on a sample with streptavidin beads alone. We also included a sample from cells treated with only vehicle treatment to exclude nonspecific and endogenous biotin-binding proteins. Importantly, we included on-bead digestion in our approach, in order to overcome the limitations of in-gel digestion [41].

Two hundred and seventy-eight individual proteins were uniquely modified by 2-ClHDyA in EA.hy926 cells. Since a total of 4596 individual proteins have been reported in EA.hy926 cells [42], the present findings indicate approximately 6% of EA.hy926 proteins are modified specifically by 2-ClHDyA. One hundred and thirty-one individual proteins were uniquely modified by 2-ClHDyA in HLMVEC. The 2-ClHDyA-modified proteins in both the metabolite interconversion enzyme and protein modifying enzyme groups were most abundantly represented in both EA.hy926 and HLMVEC (Figure 6A and Figure 8A). The proteins in the metabolite interconversion enzyme group including oxidoreductase, transferase, hydrolase, isomerase, and ligase, each of which have cysteine in their active sites. We have previously shown that 2-ClHDA interacts with protein thiols [24] and it is likely that the enrichment of proteins in the metabolite interconversion enzyme group represents a number of proteins that have been modified potentially at an active site cysteine. We have not identified the residue of proteins that are modified by 2-ClHDyA since this tryptic peptide remains bound to the streptavidin bead during tryptic digestion. However, manual analyses of tryptic peptides revealed 17/20 proteins randomly selected from the metabolite interconversion enzyme group were missing tryptic peptides containing the active site cysteine. It will be important in future studies to determine the nature of the modified peptide and confirm active site cysteines are modified accompanied by biochemical analyses of modifications in protein function.

Previous studies have demonstrated that chlorolipids accumulate in the mouse and rat lung as well as the bronchoalveolar lavage fluid following exposure to chlorine gas [43]. Additionally, 2-ClFA, produced from 2-ClFALD, accumulates in the lung in a rat sepsis model [44]. Considerable evidence suggests chlorinated lipids produced by activated neutrophils mediate endothelial dysfunction [17,18,25,44], suggesting a directionality of neutrophil-derived molecules impacting lung endothelial cells. It should also be noted that the mobilization of endothelial Weibel–Palade bodies indicates chlorinated lipids further promote neutrophil–endothelial signaling by enhancing neutrophil recruitment to the endothelium [25,45]. The present study suggests an additional layer of interaction between the endothelial cells and neutrophils, as well as other immune cells, since these proteomic studies reveal the enrichment of 2-ClHDyA-modified proteins in the granulocyte activation, neutrophil-mediated immunity and antigen processing and presentation GO biological function groups. Furthermore, regarding the Weibel–Palade body bodies, a number of proteins found in Weibel–Palade bodies were found to be targeted by 2-ClHDyA in both EA.hy926 cells and HLMVEC. It should be noted that EA.hy926 cells contain WPBs and although HLMVEC do not have WPBs, they do have separate granules containing von Willebrand factor and P-selectin (the two main components of WPBs) [46].

It should also be noted that others have also shown 2-ClHDA alters the endothelial function including blood–brain barrier dysfunction [19,20,39]. Brain microvascular endothelial cells showed morphological changes in both tight and adherens junctions [19]. The main component of the endothelial junction, VE-cadherin, interacts with β-catenin, p120-catenin, and γ-catenin. β-Catenin and γ-catenin connect to actin-binding α-catenin, and other proteins. This intracellular complex of VE-cadherin and catenins is essential for junctional stability [47]. The proteomics data herein show the GO biological processes cell junction organization, molecular function cell adhesion molecule binding, and KEGG pathway adherens junction were each significantly enriched among the proteins modified by 2-ClHDyA in HLMVEC. In addition, α-catenin, β-catenin, p120-catenin, and γ-catenin were identified as protein targets of 2-ClFALD, suggesting a potential mechanism of endothelial barrier dysfunction.

Comparisons were made between the proteins modified by 2-ClHDyA in EA.hy926 cells and HLMVECs. When examining those modified specifically by 2-ClHDyA there were only 46 identical proteins modified in both cell lines. This, however, represented over 35% of the proteins modified in HLMVEC. Due to the reduced number of overlapping proteins, it was not feasible to perform bioinformatic analyses to assess the groups and pathways common in both cells, but inspection of the list of proteins in Table S8 shows a number of proteasome-related proteins. The proteasome-related proteins potentially may have a role in the known permeability barrier dysfunction observed in endothelium treated with 2-ClHDA since proteasome-mediated proteolysis of β-catenin (also a common modified protein) may have a role in adherens junction leakiness [48].

## 5. Conclusions

The studies herein provide novel information about protein modification by 2-ClFALD in the absence of reduction. Under nonreducing conditions, it is assumed that protein modification by 2-ClFALD is mediated by an alkylation reaction similar to that observed and reactions of 2-ClFALD with glutathione [23]. Several protein targets of 2-ClFALD provide insight into potential mechanisms behind 2-ClFALD’s known effects on endothelial barrier permeability and cytotoxicity. The modification of cell junction and adhesion proteins may be one way that 2-ClFALD increases endothelial permeability. These results require further investigation to understand the functional consequences of the modifications as well as the specific peptide residues modified by 2-ClFALD. Understanding the effects of these modifications has the potential to lead to deeper knowledge of the pathophysiological sequelae mediated by MPO products, which could lead to future improved treatments of diseases mediated in part by neutrophils.

## Data Availability

All data are within the manuscript and Supplementary Materials.

## Supplementary Materials

The following supporting information can be downloaded at: https://www.mdpi.com/article/10.3390/antiox11050940/s1, Figure S1: Schematic of workflow applied to identify the proteins modified by 2-ClHDyA; Figure S2: Bioinformatic characterization of EA.hy926 cell proteins modified by 2-ClHDyA and HDyA; Figure S3: Bioinformatic characterization of HLMVEC proteins modified by 2-ClHDyA and HDyA; Table S1: List of 278 proteins modified by only 2-ClHDyA in EA.hy926 cells; Table S2: List of 394 proteins modified by 2-ClHDyA in EA.hy926 cells including those also modified by HDyA; Table S3: List of 131 proteins modified by only 2-ClHDyA in HLMVEC; Table S4: Proteins belonging to the enriched biological process “neutrophil-mediated immunity” as determined by over-representation analysis using WebGestalt; Table S5: Proteins belonging to the enriched biological process “cell redox homeostasis” as determined by over-representation analysis using WebGestalt; Table S6: Proteins belonging to the enriched KEGG pathway “adherens junction” as determined by over-representation analysis using WebGestalt; Table S7: List of 327 proteins modified by 2-ClHDyA in HLMVEC including those also modified by HDyA; Table S8: List of 46 proteins modified only by ClHDyA in both HLMVEC and EA.hy926 cells; Table S9: List of 164 proteins modified by ClHDyA and HDyA in both HLMVEC and EA.hy926 cells.
