# Supplementary material for: Endothelial Cell Protein Targeting by Myeloperoxidase-Derived 2-Chlorofatty Aldehyde
Source: Antioxidants (Basel). 2022 May 10;11(5):940. doi: 10.3390/antiox11050940 (PMC9138145; doi:10.3390/antiox11050940)
Supplement: Supplementary file 1 [file antioxidants-11-00940-s001.zip › antioxidants-1694063-supplementary.pdf]

antioxidants

Supplementary Materials: Endothelial Cell Protein Targeting by Myeloperoxidase-Derived 2-Chlorofatty Aldehyde

Shubha Shakya [1,2], Roger A. Herr [1,2], Haley L. Carlson [1,2], Raphael A. Zoeller [3], Carolyn J. Albert [1,2] and David A. Ford [1,2,*]

[1] Center for Cardiovascular Research, Saint Louis University School of Medicine, St. Louis, MO 63104, USA; shubha.shakya@slu.edu (S.S.); r.a.herr@gmail.com (R.A.H.); haley.carlson@slu.edu (H.L.C.); carolyn.albert@health.slu.edu (C.J.A.)
[2] Edward A. Doisy Department of Biochemistry and Molecular Biology, Saint Louis University School of Medicine, St. Louis, MO 63104, USA
[3] Department of Physiology and Biophysics, Boston University School of Medicine, Boston, MA 02118, USA; rzoeller@bu.edu
* Correspondence: david.ford@health.slu.edu

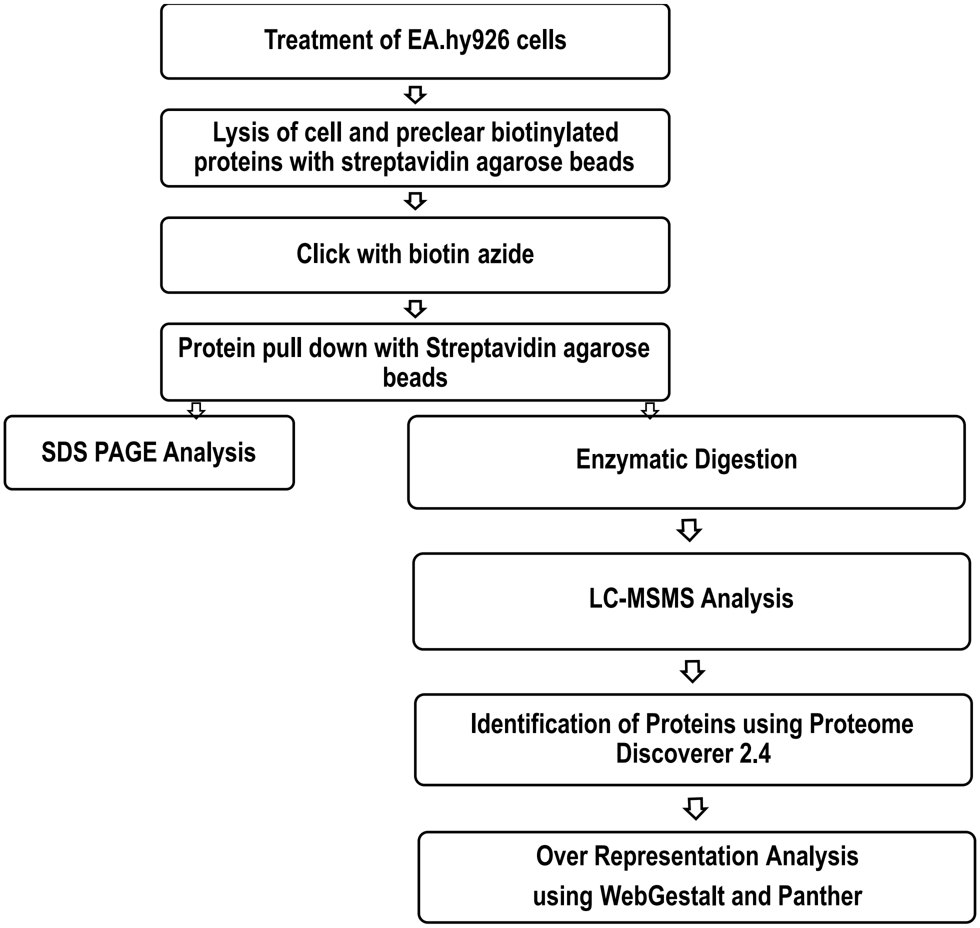

Figure S1. Schematic of workflow applied to identify the proteins modified by 2-ClHDyA. EA.hy926 cells were treated with DMSO, 10 µM HDyA or 10 µM ClHDyA for 1 h. Cells were lysed, clicked with azide-PEG3-biotin and incubated with streptavidin agarose beads. Streptavidin purified proteins were either used for SDS PAGE analysis or on-bead digestion. The digested peptides were subjected to LC-MS/MS and peptides were analyzed using and Proteome Discoverer 2.4 software. The WebGestalt tool was used to perform Over representation analysis.

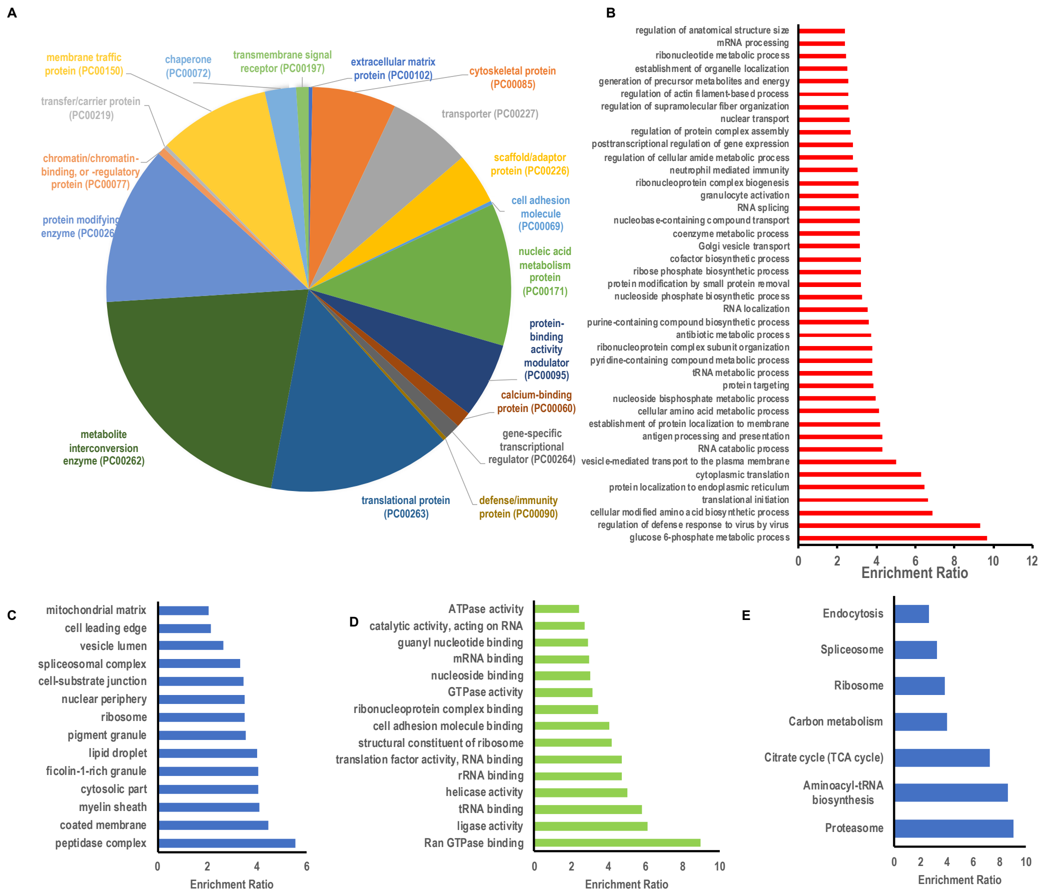

Figure S2: Bioinformatic characterization of EA.hy926 cell proteins modified by 2-ClHDyA and HDyA: (A) Distribution of 2-ClHDyA-modified proteins by protein class analyzed using Panther. (B-E) Over representation analysis using the Web-based Gene Set Analysis Toolkit (WebGestalt). Enriched categories in Gene ontology (GO) Biological function (B), Cellular component (C), Molecular function (D), and KEGG Pathway using Bonferroni P<0.05 for computing FDR (p<0.05) (E).

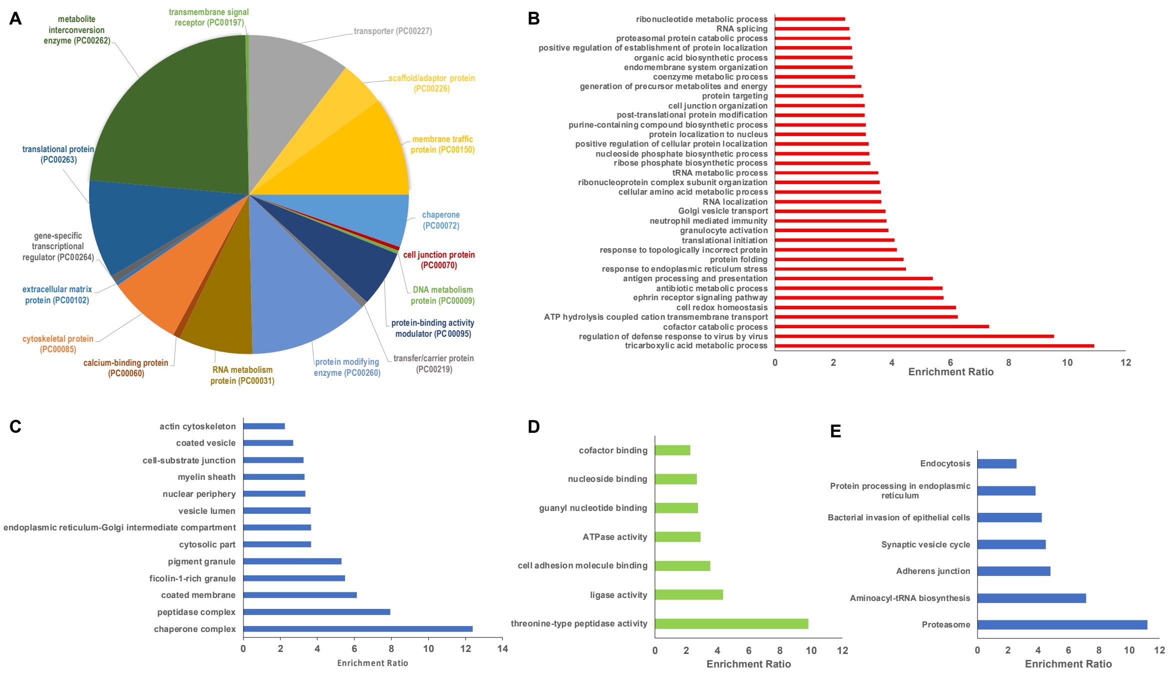

Figure S3: Bioinformatic characterization of HLMVEC proteins modified by 2-ClHDyA and HDyA: (A) Distribution of 2-ClHDyA-modified proteins by protein class analyzed using Panther. (B-E) Over representation analysis using the Web-based Gene Set Analysis Toolkit (WebGestalt). Enriched categories in Gene ontology (GO) Biological function (B), Cellular component (C), Molecular function (D), and KEGG Pathway using Bonferroni P<0.05 for computing FDR (p<0.05) (E).

Table S1: List of 278 proteins modified by only 2-ClHDyA in EA.hy926 cells

Accession	Description	Gene Symbol	# Peptides	# PSMs	# Unique Peptides	Coverage [%]	Sum PEP Score	Biological Process
P61221	ATP-binding cassette sub-family E member 1 [OS=Homo sapiens]	ABCE1	6	44	6	13	21.202	cell organization and biogenesis;metabolic process;regulation of biological process;transport
P09110-1	3-ketoacyl-CoA thiolase, peroxisomal [OS=Homo sapiens]	ACAA1	10	131	10	38	60.632	metabolic process;transport
P42765	3-ketoacyl-CoA thiolase, mitochondrial [OS=Homo sapiens]	ACAA2	6	42	6	28	22.725	cell organization and biogenesis;metabolic process;regulation of biological process;response to stimulus
Q13085-1	Acetyl-CoA carboxylase 1 [OS=Homo sapiens]	ACACA	6	41	6	3	24.463	cell organization and biogenesis;metabolic process;regulation of biological process;response to stimulus;transport
P24752	Acetyl-CoA acetyltransferase, mitochondrial [OS=Homo sapiens]	ACAT1	13	130	13	43	61.3	cell organization and biogenesis;metabolic process;response to stimulus
Q99798	Aconitate hydratase, mitochondrial [OS=Homo sapiens]	ACO2	5	18	5	10	17.453	metabolic process;response to stimulus
Q9Y305-1	Acyl-coenzyme A thioesterase 9, mitochondrial [OS=Homo sapiens]	ACOT9	4	30	4	10	14.675	metabolic process
O95573	long-chain-fatty-acid--CoA ligase 3 [OS=Homo sapiens]	ACSL3	5	46	3	10	21.683	cell organization and biogenesis;metabolic process;regulation of biological process;response to stimulus;transport
O60488-1	Long-chain-fatty-acid--CoA ligase 4 [OS=Homo sapiens]	ACSL4	6	44	4	12	24.194	cell differentiation;metabolic process;regulation of biological process;response to stimulus;transport
P61163	Alpha-centractin [OS=Homo sapiens]	ACTR1A	5	62	5	24	26.894	regulation of biological process;transport
P61158	actin-related protein 3 [OS=Homo sapiens]	ACTR3	8	81	8	29	45.111	cell division;cell organization and biogenesis;cellular component

								movement;regulation of biological process;response to stimulus
P37023	Serine/threonine-protein kinase receptor R3 [OS=Homo sapiens]	ACVRL1	2	16	2	6	8.395	cell differentiation;cell organization and biogenesis;cell proliferation;cellular component movement;metabolic process;regulation of biological process;response to stimulus
P30520	adenylosuccinate synthetase isozyme 2 [OS=Homo sapiens]	ADSS	4	54	4	13	26.475	metabolic process;response to stimulus
O00116	Alkyldihydroxyacetonephosphate synthase, peroxisomal [OS=Homo sapiens]	AGPS	3	47	3	6	16.795	metabolic process
Q13155	aminoacyl tRNA synthase complex-interacting multifunctional protein 2 [OS=Homo sapiens]	AIMP2	3	21	3	17	12.218	cell death;cell differentiation;cell organization and biogenesis;development;metabol ic process;regulation of biological process
P00568	Adenylate kinase isoenzyme 1 [OS=Homo sapiens]	AK1	2	31	2	13	7.818	metabolic process
P15121	aldose reductase [OS=Homo sapiens]	AKR1B1	5	92	4	11	25.171	metabolic process;regulation of biological process;response to stimulus
P49419	Alpha-aminoadipic semialdehyde dehydrogenase [OS=Homo sapiens]	ALDH7A1	5	25	5	15	17.78	metabolic process
P08133-1	annexin A6 [OS=Homo sapiens]	ANXA6	4	31	4	7	13.608	cell organization and biogenesis;cellular homeostasis;regulation of biological process;response to stimulus;transport
P20073-1	Annexin A7 [OS=Homo sapiens]	ANXA7	4	48	4	10	20.365	cell differentiation;cell organization and biogenesis;cell proliferation;cellular homeostasis;metabolic process;regulation of biological process;response to stimulus
Q9BXS5-1	AP-1 complex subunit mu-1 [OS=Homo sapiens]	AP1M1	5	20	5	17	15.492	cell organization and biogenesis;regulation of biological process;transport
O95782	AP-2 complex subunit alpha-1 [OS=Homo sapiens]	AP2A1	8	94	3	11	31.456	cell organization and biogenesis;cellular component

								movement;metabolic process;regulation of biological process;response to stimulus;transport
O94973	AP-2 complex subunit alpha-2 [OS=Homo sapiens]	AP2A2	9	71	4	13	30.102	cell organization and biogenesis;cellular component movement;metabolic process;regulation of biological process;response to stimulus;transport
P63010-1	AP-2 complex subunit beta [OS=Homo sapiens]	AP2B1	7	101	7	8	30.303	cell organization and biogenesis;cellular component movement;metabolic process;regulation of biological process;response to stimulus;transport
Q96CW1	AP-2 complex subunit mu [OS=Homo sapiens]	AP2M1	5	49	5	14	26.689	cell organization and biogenesis;cellular component movement;metabolic process;regulation of biological process;response to stimulus;transport
O00203-1	AP-3 complex subunit beta-1 [OS=Homo sapiens]	AP3B1	2	22	2	3	7.489	cell organization and biogenesis;cellular component movement;coagulation;regulation of biological process;transport
Q9Y2T2	ap-3 complex subunit mu-1 [OS=Homo sapiens]	AP3M1	2	23	2	6	7.702	cellular component movement;transport
P61204	ADP-ribosylation factor 3 [OS=Homo sapiens]	ARF3	7	86	4	51	42.394	metabolic process;regulation of biological process;response to stimulus;transport
P18085	ADP-ribosylation factor 4 [OS=Homo sapiens]	ARF4	6	49	3	41	27.618	cellular component movement;metabolic process;regulation of biological process;response to stimulus;transport
P62330	ADP-ribosylation factor 6 [OS=Homo sapiens]	ARF6	2	37	2	12	10.531	cell death;cell differentiation;cell division;cell organization and biogenesis;cellular component movement;regulation of biological process;response to stimulus;transport

Q8N392-1	Rho GTPase-activating protein 18 [OS=Homo sapiens]	ARHGAP 18	3	17	3	5	11.019	cell organization and biogenesis;regulation of biological process;response to stimulus
P40616	ADP-ribosylation factor-like protein 1 [OS=Homo sapiens]	ARL1	3	46	3	19	16.567	cell organization and biogenesis;metabolic process;regulation of biological process;response to stimulus;transport
O15143	Actin-related protein 2/3 complex subunit 1B [OS=Homo sapiens]	ARPC1B	3	45	3	11	16.043	cell organization and biogenesis;cellular component movement;regulation of biological process;response to stimulus
O15144	Actin-related protein 2/3 complex subunit 2 [OS=Homo sapiens]	ARPC2	4	41	4	12	14.602	cell organization and biogenesis;cellular component movement;regulation of biological process;response to stimulus
Q6DD88	Atlastin-3 [OS=Homo sapiens]	ATL3	4	66	4	11	27.694	cell organization and biogenesis;regulation of biological process;transport
Q9NQ88	Fructose-2,6-bisphosphatase TIGAR [OS=Homo sapiens]	C12orf5; TIGAR	2	19	2	10	8.744	cell death;metabolic process;regulation of biological process;response to stimulus
Q13557	Calcium/calmodulin-dependent protein kinase type II subunit delta [OS=Homo sapiens]	CAMK2D	3	41	3	7	14.872	cell differentiation;cell organization and biogenesis;cellular component movement;cellular homeostasis;metabolic process;regulation of biological process;response to stimulus
P27824	Calnexin [OS=Homo sapiens]	CANX	4	27	4	8	18.981	metabolic process;regulation of biological process;response to stimulus;transport
P07384	Calpain-1 catalytic subunit [OS=Homo sapiens]	CAPN1	4	51	4	7	22.1	cell death;cell organization and biogenesis;metabolic process;regulation of biological process;transport
P52907	F-actin-capping protein subunit alpha-1 [OS=Homo sapiens]	CAPZA1	3	46	2	14	13.776	cell organization and biogenesis;cellular component movement;coagulation;defense response;regulation of biological

								process;response to stimulus;transport
P47755	F-actin-capping protein subunit alpha-2 [OS=Homo sapiens]	CAPZA2	3	53	2	14	18.233	cell organization and biogenesis;cellular component movement;coagulation;defense response;regulation of biological process;response to stimulus;transport
P04040	catalase [OS=Homo sapiens]	CAT	3	22	3	9	16.407	cell differentiation;cell organization and biogenesis;metabolic process;regulation of biological process;response to stimulus;transport
Q16543	Hsp90 co-chaperone Cdc37 [OS=Homo sapiens]	CDC37	4	42	4	14	19.443	cell division;metabolic process;regulation of biological process;response to stimulus;transport
Q00535	Cyclin-dependent-like kinase 5 [OS=Homo sapiens]	CDK5	3	18	3	9	12.872	cell communication;cell death;cell differentiation;cell division;cell growth;cell organization and biogenesis;cell proliferation;cellular component movement;metabolic process;regulation of biological process;response to stimulus;transport
O00299	chloride intracellular channel protein 1 [OS=Homo sapiens]	CLIC1	7	124	7	45	47.443	metabolic process;regulation of biological process;response to stimulus;transport
Q9Y696	Chloride intracellular channel protein 4 [OS=Homo sapiens]	CLIC4	8	46	8	43	27.706	cell differentiation;cell organization and biogenesis;cellular homeostasis;metabolic process;regulation of biological process;response to stimulus;transport
Q96KP4	cytosolic non-specific dipeptidase [OS=Homo sapiens]	CNDP2	3	15	3	10	8.701	metabolic process
Q8NBJ5	Procollagen galactosyltransferase 1 [OS=Homo sapiens]	COLGALT1; GLT25D1	5	38	5	9	21.911	

P21964-1	Catechol O-methyltransferase [OS=Homo sapiens]	COMT	4	35	4	20	22.626	cell communication;metabolic process;regulation of biological process;response to stimulus
P61923	Coatomer subunit zeta-1 [OS=Homo sapiens]	COPZ1	3	30	3	27	14.817	transport
Q99829	Copine-1 [OS=Homo sapiens]	CPNE1	4	42	4	7	17.563	cell differentiation;cell growth;metabolic process;regulation of biological process;response to stimulus;transport
P36551	Oxygen-dependent coproporphyrinogen-III oxidase, mitochondrial [OS=Homo sapiens]	CPOX	4	36	4	12	18.099	metabolic process;response to stimulus
P41240	tyrosine-protein kinase CSK [OS=Homo sapiens]	CSK	3	14	3	8	9.862	cell differentiation;cell organization and biogenesis;defense response;metabolic process;regulation of biological process;response to stimulus
P56545	c-terminal-binding protein 2 [OS=Homo sapiens]	CTBP2	2	25	1	6	9.605	cell differentiation;metabolic process;regulation of biological process
P35222	Catenin beta-1 [OS=Homo sapiens]	CTNNB1	9	43	6	17	44.974	cell communication;cell differentiation;cell growth;cell organization and biogenesis;cell proliferation;cellular component movement;metabolic process;regulation of biological process;response to stimulus;transport
O43169	Cytochrome b5 type B [OS=Homo sapiens]	CYB5B	3	49	3	45	24.678	metabolic process;transport
Q6PI48	Aspartate--tRNA ligase, mitochondrial [OS=Homo sapiens]	DARS2	3	37	3	6	13.475	metabolic process
Q9NR30-1	Nucleolar RNA helicase 2 [OS=Homo sapiens]	DDX21	6	42	6	9	21.475	cell differentiation;metabolic process;regulation of biological process;response to stimulus
O00148	ATP-dependent RNA helicase DDX39A [OS=Homo sapiens]	DDX39A	9	184	2	23	48.632	metabolic process;regulation of biological process;response to stimulus;transport

Q13838-1	spliceosome RNA helicase DDX39B [OS=Homo sapiens]	DDX39B	10	183	3	23	51.709	cell organization and biogenesis;metabolic process;regulation of biological process;transport
Q86XP3-1	ATP-dependent RNA helicase DDX42 [OS=Homo sapiens]	DDX42	3	22	3	3	10.88	metabolic process;regulation of biological process
Q7L014	probable ATP-dependent RNA helicase DDX46 [OS=Homo sapiens]	DDX46	8	106	8	10	36.291	metabolic process;regulation of biological process
P26196	Probable ATP-dependent RNA helicase DDX6 [OS=Homo sapiens]	DDX6	7	72	7	20	32.456	cell organization and biogenesis;metabolic process;regulation of biological process
P23743-1	diacylglycerol kinase alpha [OS=Homo sapiens]	DGKA	3	12	3	6	9.986	metabolic process;regulation of biological process;response to stimulus
P09622	Dihydrolipoyl dehydrogenase, mitochondrial [OS=Homo sapiens]	DLD	4	42	4	13	15.742	cellular homeostasis;metabolic process;regulation of biological process
P36957	Dihydrolipoyllysine-residue succinyltransferase component of 2-oxoglutarate dehydrogenase complex, mitochondrial [OS=Homo sapiens]	DLST	3	56	3	8	22.62	metabolic process
Q9UDY4	DnaJ homolog subfamily B member 4 [OS=Homo sapiens]	DNAJB4	4	44	3	17	14.628	metabolic process;regulation of biological process;response to stimulus
Q8IXB1-1	DnaJ homolog subfamily C member 10 [OS=Homo sapiens]	DNAJC10	5	38	4	6	15.207	cellular homeostasis;metabolic process;regulation of biological process;response to stimulus
Q9Y295	developmentally-regulated GTP-binding protein 1 [OS=Homo sapiens]	DRG1	6	65	6	23	29.759	development;metabolic process
O75923-1	dysferlin [OS=Homo sapiens]	DYSF	3	20	3	2	13.4	cell organization and biogenesis
Q15029	116 kDa U5 small nuclear ribonucleoprotein component [OS=Homo sapiens]	EFTUD2	2	55	1	2	9.595	cell organization and biogenesis;metabolic process;response to stimulus
Q9NZN4	EH domain-containing protein 2 [OS=Homo sapiens]	EHD2	6	49	5	12	28.195	cell organization and biogenesis;coagulation;regulation of biological process;transport
Q9BY44-1	Eukaryotic translation initiation factor 2A [OS=Homo sapiens]	EIF2A	3	24	3	8	11.168	cell organization and biogenesis;metabolic

								process;regulation of biological process;response to stimulus
P19525	interferon-induced, double-stranded RNA-activated protein kinase [OS=Homo sapiens]	EIF2AK2	2	17	2	4	6.913	cell communication;defense response;metabolic process;regulation of biological process;response to stimulus
Q14152	Eukaryotic translation initiation factor 3 subunit A [OS=Homo sapiens]	EIF3A	12	109	12	11	53.257	cell organization and biogenesis;metabolic process;regulation of biological process
P60228	Eukaryotic translation initiation factor 3 subunit E [OS=Homo sapiens]	EIF3E	4	44	4	11	17.353	metabolic process;regulation of biological process
Q9Y262	eukaryotic translation initiation factor 3 subunit L [OS=Homo sapiens]	EIF3L	4	32	4	10	21.31	metabolic process
Q14240-1	eukaryotic initiation factor 4A-II [OS=Homo sapiens]	EIF4A2	10	354	2	27	79.092	metabolic process;regulation of biological process
P38919	Eukaryotic initiation factor 4A-III [OS=Homo sapiens]	EIF4A3	4	60	3	12	22.959	metabolic process;regulation of biological process;response to stimulus;transport
Q04637	eukaryotic translation initiation factor 4 gamma 1 [OS=Homo sapiens]	EIF4G1	3	16	3	2	13.446	cell organization and biogenesis;defense response;metabolic process;regulation of biological process;response to stimulus
Q6IS14	Eukaryotic translation initiation factor 5A-1-like [OS=Homo sapiens]	EIF5AL1	3	13	3	33	8.843	metabolic process;regulation of biological process;transport
P17813	Endoglin [OS=Homo sapiens]	ENG	7	47	7	13	31.57	cell differentiation;cell organization and biogenesis;cellular component movement;regulation of biological process;response to stimulus;transport
P29317	Ephrin type-A receptor 2 [OS=Homo sapiens]	EPHA2	4	27	4	4	15.174	cell death;cell differentiation;cell organization and biogenesis;cell proliferation;cellular component movement;defense response;development;metabolic process;regulation of biological process;response to stimulus

Q9NZ08-1	endoplasmic reticulum aminopeptidase 1 [OS=Homo sapiens]	ERAP1	7	46	7	8	22.243	cell communication;cell differentiation;metabolic process;regulation of biological process;response to stimulus
P84090	Enhancer of rudimentary homolog [OS=Homo sapiens]	ERH	2	28	2	28	11.73	metabolic process;regulation of biological process
Q9BSJ8	Extended synaptotagmin-1 [OS=Homo sapiens]	ESYT1	7	46	7	8	32.169	metabolic process;transport
A0FGR8-1	Extended synaptotagmin-2 [OS=Homo sapiens]	ESYT2	7	48	7	10	28.763	metabolic process;transport
P62495	Eukaryotic peptide chain release factor subunit 1 [OS=Homo sapiens]	ETF1	5	60	5	14	25.537	cell organization and biogenesis;metabolic process;regulation of biological process
P13804-1	Electron transfer flavoprotein subunit alpha, mitochondrial [OS=Homo sapiens]	ETFA	3	41	3	14	14.415	metabolic process;transport
P15311	Ezrin [OS=Homo sapiens]	EZR	12	351	3	19	64.669	cell differentiation;cell organization and biogenesis;cellular component movement;metabolic process;regulation of biological process;response to stimulus;transport
Q9NZB2-1	Constitutive coactivator of PPAR-gamma-like protein 1 [OS=Homo sapiens]	FAM120A	6	38	6	8	16.621	
Q96TA1	Niban-like protein 1 [OS=Homo sapiens]	FAM129B	8	106	8	12	36.864	cell differentiation;cellular component movement;metabolic process;regulation of biological process;transport
Q9BRX8-1	Redox-regulatory protein FAM213A [OS=Homo sapiens]	FAM213A	8	170	8	34	41.341	metabolic process;regulation of biological process
Q9NSD9	Phenylalanine--tRNA ligase beta subunit [OS=Homo sapiens]	FARSB	5	60	5	9	19.341	cell organization and biogenesis;metabolic process
P39748	Flap endonuclease 1 [OS=Homo sapiens]	FEN1	2	25	2	8	9.925	cell organization and biogenesis;cellular homeostasis;metabolic process;regulation of biological process;response to stimulus

P07954	fumarate hydratase, mitochondrial [OS=Homo sapiens]	FH	3	44	3	10	18.554	metabolic process
P51570	galactokinase [OS=Homo sapiens]	GALK1	3	29	3	11	11.034	metabolic process
Q04446	1,4-alpha-glucan-branching enzyme [OS=Homo sapiens]	GBE1	6	47	6	15	27.184	metabolic process
Q06210-1	glutamine--fructose-6-phosphate aminotransferase [isomerizing] 1 [OS=Homo sapiens]	GFPT1	7	48	7	14	24.276	metabolic process;regulation of biological process
P04899-1	guanine nucleotide-binding protein G(i) subunit alpha-2 [OS=Homo sapiens]	GNAI2	7	82	6	30	26.672	cell division;cell proliferation;metabolic process;regulation of biological process;response to stimulus
Q5JWF2-1	Guanine nucleotide-binding protein G(S) subunit alpha isoforms XLas [OS=Homo sapiens]	GNAS	4	80	3	5	21.463	metabolic process;regulation of biological process;response to stimulus
P62873	Guanine nucleotide-binding protein G(I)/G(S)/G(T) subunit beta-1 [OS=Homo sapiens]	GNB1	4	33	4	14	21.165	cell death;cell organization and biogenesis;cell proliferation;cellular homeostasis;metabolic process;regulation of biological process;response to stimulus
P62879	Guanine nucleotide-binding protein G(I)/G(S)/G(T) subunit beta-2 [OS=Homo sapiens]	GNB2	3	42	3	10	21.707	metabolic process;regulation of biological process;response to stimulus
Q9Y2Q3-1	Glutathione S-transferase kappa 1 [OS=Homo sapiens]	GSTK1	3	39	3	20	14.809	cell differentiation;metabolic process
P40939	Trifunctional enzyme subunit alpha, mitochondrial [OS=Homo sapiens]	HADHA	11	180	11	20	71.612	metabolic process;response to stimulus
Q00341-1	Vigilin [OS=Homo sapiens]	HDLBP	11	127	11	12	47.817	metabolic process;transport
Q8TCT9-1	Minor histocompatibility antigen H13 [OS=Homo sapiens]	HM13	3	26	3	12	16.831	cell organization and biogenesis;metabolic process
P51991-1	Heterogeneous nuclear ribonucleoprotein A3 [OS=Homo sapiens]	HNRNPA3	7	95	5	20	39.782	metabolic process;transport
P52597	Heterogeneous nuclear ribonucleoprotein F [OS=Homo sapiens]	HNRNPF	7	181	5	26	47.484	metabolic process;regulation of biological process;response to stimulus

P55795	heterogeneous nuclear ribonucleoprotein H2 [OS=Homo sapiens]	HNRNPH2	12	303	5	34	94.078	metabolic process
P31942	Heterogeneous nuclear ribonucleoprotein H3 [OS=Homo sapiens]	HNRNPH3	4	72	3	17	24.351	cell differentiation;metabolic process
P14866	Heterogeneous nuclear ribonucleoprotein L [OS=Homo sapiens]	HNRNPL	7	43	7	19	21.286	metabolic process;regulation of biological process
O43390-1	heterogeneous nuclear ribonucleoprotein r [OS=Homo sapiens]	HNRNPR	5	67	2	9	20.585	metabolic process;regulation of biological process
Q5SSJ5-1	Heterochromatin protein 1-binding protein 3 [OS=Homo sapiens]	HP1BP3	6	62	6	11	22.697	cell organization and biogenesis;regulation of biological process;response to stimulus
P51659-1	peroxisomal multifunctional enzyme type 2 [OS=Homo sapiens]	HSD17B4	5	34	5	10	22.901	cell differentiation;metabolic process
Q92598	Heat shock protein 105 kDa [OS=Homo sapiens]	HSPH1	12	154	10	19	76.728	metabolic process;regulation of biological process;response to stimulus;transport
Q7Z6Z7	E3 ubiquitin-protein ligase HUWE1 [OS=Homo sapiens]	HUWE1	9	42	9	3	35.443	cell differentiation;cell organization and biogenesis;metabolic process;response to stimulus;transport
Q9NSE4	Isoleucine--tRNA ligase, mitochondrial [OS=Homo sapiens]	IARS2	5	49	5	6	23.511	metabolic process;regulation of biological process
Q16666-1	gamma-interferon-inducible protein 16 [OS=Homo sapiens]	IFI16	5	56	5	8	23.879	cell communication;cell death;cell differentiation;cell proliferation;defense response;metabolic process;regulation of biological process;response to stimulus
Q9NZI8	Insulin-like growth factor 2 mRNA-binding protein 1 [OS=Homo sapiens]	IGF2BP1	6	121	5	14	34.943	cell proliferation;regulation of biological process;transport
Q9Y6M1	Insulin-like growth factor 2 mRNA-binding protein 2 [OS=Homo sapiens]	IGF2BP2	8	102	6	18	40.57	regulation of biological process;transport

Q70UQ0	Inhibitor of nuclear factor kappa-B kinase-interacting protein [OS=Homo sapiens]	IKBIP	7	46	5	20	27.242	response to stimulus
Q70UQ0-4	Isoform 4 of Inhibitor of nuclear factor kappa-B kinase-interacting protein [OS=Homo sapiens]	IKBIP	10	53	8	31	47.702	response to stimulus
Q8TEX9-1	Importin-4 [OS=Homo sapiens]	IPO4	5	28	5	5	21.79	cell organization and biogenesis;transport
O95373	Importin-7 [OS=Homo sapiens]	IPO7	7	51	6	9	30.846	defense response;regulation of biological process;response to stimulus;transport
Q96P70	Importin-9 [OS=Homo sapiens]	IPO9	2	23	2	4	19.99	transport
Q15046	Lysine--tRNA ligase [OS=Homo sapiens]	KARS	10	46	10	19	34.724	metabolic process;regulation of biological process;response to stimulus
O00139	Kinesin-like protein KIF2A [OS=Homo sapiens]	KIF2A	3	20	3	4	9.089	cell differentiation;cell division;cell organization and biogenesis;cellular component movement;development;transpor t
Q14974	Importin subunit beta-1 [OS=Homo sapiens]	KPNB1	6	66	6	9	27.52	cell organization and biogenesis;cellular component movement;metabolic process;regulation of biological process;response to stimulus;transport
Q9P2J5	Leucine--tRNA ligase, cytoplasmic [OS=Homo sapiens]	LARS	9	114	9	10	38.862	cell communication;cell organization and biogenesis;metabolic process;regulation of biological process;response to stimulus;transport
P49257	Protein ERGIC-53 [OS=Homo sapiens]	LMAN1	3	36	3	6	10.963	cell organization and biogenesis;coagulation;metabolic process;regulation of biological process;transport
P42704	Leucine-rich PPR motif-containing protein, mitochondrial [OS=Homo sapiens]	LRPPRC	12	107	12	12	54.149	cellular component movement;metabolic process;regulation of biological process;transport
P09960-1	leukotriene A-4 hydrolase [OS=Homo sapiens]	LTA4H	6	68	6	12	44.086	metabolic process;transport

Q9UPN3	Microtubule-actin cross-linking factor 1, isoforms 1/2/3/5 [OS=Homo sapiens]	MACF1	15	128	4	2	52.118	cell organization and biogenesis;regulation of biological process;response to stimulus;transport
P27816-1	Microtubule-associated protein 4 [OS=Homo sapiens]	MAP4	3	23	3	4	9.727	cell division;cell organization and biogenesis;cellular component movement;regulation of biological process
P56192	Methionine--tRNA ligase, cytoplasmic [OS=Homo sapiens]	MARS	4	45	4	6	14.988	cell communication;metabolic process;regulation of biological process;response to stimulus
P49736	DNA replication licensing factor mcm2 [OS=Homo sapiens]	MCM2	5	53	5	7	22.048	cell death;cell organization and biogenesis;metabolic process;response to stimulus
Q14566	DNA replication licensing factor MCM6 [OS=Homo sapiens]	MCM6	5	52	5	10	25.499	metabolic process
P33993-1	DNA replication licensing factor MCM7 [OS=Homo sapiens]	MCM7	7	47	7	13	22.492	cell proliferation;metabolic process;regulation of biological process;response to stimulus
O60502	protein O-GlcNAcase [OS=Homo sapiens]	MGEA5	3	10	3	4	11.843	metabolic process
Q6UB35-1	Monofunctional C1-tetrahydrofolate synthase, mitochondrial [OS=Homo sapiens]	MTHFD1L	3	33	3	4	13.247	metabolic process
Q9BXJ9	N-alpha-acetyltransferase 15, NatA auxiliary subunit [OS=Homo sapiens]	NAA15	6	55	6	7	24.723	cell differentiation;development;metabolic process;regulation of biological process
O43776	Asparagine--tRNA ligase, cytoplasmic [OS=Homo sapiens]	NARS	5	37	5	9	22.133	metabolic process
Q6PIU2	Neutral cholesterol ester hydrolase 1 [OS=Homo sapiens]	NCEH1	6	123	6	23	41.476	metabolic process
Q9Y2A7-1	Nck-associated protein 1 [OS=Homo sapiens]	NCKAP1	7	43	7	9	22.595	cell death;cell organization and biogenesis;regulation of biological process;response to stimulus
O75251	NADH dehydrogenase [ubiquinone] iron-sulfur protein 7, mitochondrial [OS=Homo sapiens]	NDUFS7	2	27	2	11	13.756	cell organization and biogenesis;metabolic process;transport

Q8TD19	Serine/threonine-protein kinase Nek9 [OS=Homo sapiens]	NEK9	2	20	2	2	10.033	cell division;cell organization and biogenesis;metabolic process
P55769	NHP2-like protein 1 [OS=Homo sapiens]	NHP2L1; SNU13	2	17	2	19	8.397	cell organization and biogenesis;metabolic process
O00567	Nucleolar protein 56 [OS=Homo sapiens]	NOP56	6	49	6	16	24.337	metabolic process
P06748	Nucleophosmin [OS=Homo sapiens]	NPM1	3	42	3	14	13.756	cell growth;cell organization and biogenesis;cellular homeostasis;metabolic process;regulation of biological process;response to stimulus;transport
Q9BSD7	Cancer-related nucleoside-triphosphatase [OS=Homo sapiens]	NTPCR	2	25	2	14	12.919	metabolic process;response to stimulus
Q8N1F7-1	Nuclear pore complex protein Nup93 [OS=Homo sapiens]	NUP93	3	21	3	5	11.066	cell organization and biogenesis;cellular component movement;metabolic process;regulation of biological process;response to stimulus;transport
P04181-1	Ornithine aminotransferase, mitochondrial [OS=Homo sapiens]	OAT	5	48	5	14	21.912	cell organization and biogenesis;metabolic process
Q56VL3	OCIA domain-containing protein 2 [OS=Homo sapiens]	OCIAD2	3	23	3	20	11.771	
Q96FW1	Ubiquitin thioesterase otub1 [OS=Homo sapiens]	OTUB1	3	23	3	13	11.909	metabolic process;regulation of biological process;response to stimulus
P07237	Protein disulfide-isomerase [OS=Homo sapiens]	P4HB	5	82	5	11	26.87	cell organization and biogenesis;cellular homeostasis;metabolic process;regulation of biological process;response to stimulus
Q13177	Serine/threonine-protein kinase PAK 2 [OS=Homo sapiens]	PAK2	5	26	5	14	16.71	cell death;cell organization and biogenesis;cellular component movement;metabolic process;regulation of biological process;response to stimulus
Q99497	protein/nucleic acid deglycase DJ-1 [OS=Homo sapiens]	PARK7	6	49	6	46	21.783	cell communication;cell organization and biogenesis;defense

								response;metabolic process;regulation of biological process;response to stimulus;transport
P09874	Poly [ADP-ribose] polymerase 1 [OS=Homo sapiens]	PARP1	6	51	6	7	24.494	cell death;cell differentiation;cell organization and biogenesis;metabolic process;regulation of biological process;response to stimulus
P08559	Pyruvate dehydrogenase E1 component subunit alpha, somatic form, mitochondrial [OS=Homo sapiens]	PDHA1	4	39	4	11	18.638	metabolic process;regulation of biological process
P11177	Pyruvate dehydrogenase E1 component subunit beta, mitochondrial [OS=Homo sapiens]	PDHB	6	69	6	22	30.202	metabolic process;regulation of biological process
Q14554	Protein disulfide-isomerase A5 [OS=Homo sapiens]	PDIA5	4	23	4	10	13.463	cellular homeostasis;metabolic process;regulation of biological process;response to stimulus
P16284	Platelet endothelial cell adhesion molecule [OS=Homo sapiens]	PECAM1	7	77	7	14	34.047	cell organization and biogenesis;cellular component movement;regulation of biological process;response to stimulus;transport
P12955	xaa-Pro dipeptidase [OS=Homo sapiens]	PEPD	2	35	2	4	8.018	metabolic process
P17858-1	ATP-dependent 6-phosphofructokinase, liver type [OS=Homo sapiens]	PFKL	4	100	2	7	31.944	cell organization and biogenesis;metabolic process;regulation of biological process;response to stimulus;transport
P35080-2	Isoform IIb of Profilin-2 [OS=Homo sapiens]	PFN2	3	50	3	26	19.509	cell organization and biogenesis;regulation of biological process
P18669	Phosphoglycerate mutase 1 [OS=Homo sapiens]	PGAM1; LOC643576	5	58	5	26	26.652	metabolic process;regulation of biological process;transport
Q96G03	Phosphoglucomutase-2 [OS=Homo sapiens]	PGM2	3	36	2	7	14.683	metabolic process;transport
Q6PCE3	glucose 1,6-bisphosphate synthase [OS=Homo sapiens]	PGM2L1	6	51	6	10	18.964	metabolic process

O60664-1	Perilipin-3 [OS=Homo sapiens]	PLIN3	5	31	5	18	26.583	transport
Q02809	Procollagen-lysine,2-oxoglutarate 5-dioxygenase 1 [OS=Homo sapiens]	PLOD1	2	10	2	4	9.28	metabolic process;response to stimulus
O60568	Procollagen-lysine,2-oxoglutarate 5-dioxygenase 3 [OS=Homo sapiens]	PLOD3	2	32	2	4	11.701	cell organization and biogenesis;metabolic process;response to stimulus
P29590	Protein PML [OS=Homo sapiens]	PML	6	27	6	10	26.218	cell death;cell differentiation;cell organization and biogenesis;cellular component movement;cellular homeostasis;defense response;metabolic process;regulation of biological process;response to stimulus;transport
Q8IY17-1	Neuropathy target esterase [OS=Homo sapiens]	PNPLA6	4	47	4	5	18.904	metabolic process
Q9H488-1	GDP-fucose protein O-fucosyltransferase 1 [OS=Homo sapiens]	POFUT1	2	40	2	7	9.933	metabolic process;regulation of biological process;response to stimulus
P36873-1	serine/threonine-protein phosphatase PP1-gamma catalytic subunit [OS=Homo sapiens]	PPP1CC	4	35	2	18	20.548	cell differentiation;cell division;cell organization and biogenesis;metabolic process;regulation of biological process;response to stimulus
P62714	serine/threonine-protein phosphatase 2A catalytic subunit beta isoform [OS=Homo sapiens]	PPP2CB	4	36	4	18	19.018	cell organization and biogenesis;metabolic process;regulation of biological process;response to stimulus
O60831	PRA1 family protein 2 [OS=Homo sapiens]	PRAF2	2	21	2	12	10.283	transport
P30048	Thioredoxin-dependent peroxide reductase, mitochondrial [OS=Homo sapiens]	PRDX3	2	29	2	10	10.072	cell death;cell differentiation;cell organization and biogenesis;cellular homeostasis;metabolic process;regulation of biological process;response to stimulus
P30044-1	Peroxiredoxin-5, mitochondrial [OS=Homo sapiens]	PRDX5	6	76	6	36	23.842	cell death;cellular homeostasis;defense response;metabolic

								process;regulation of biological process;response to stimulus
O43172-1	U4/U6 small nuclear ribonucleoprotein Prp4 [OS=Homo sapiens]	PRPF4	2	12	2	6	9.526	metabolic process
Q6P2Q9	Pre-mRNA-processing-splicing factor 8 [OS=Homo sapiens]	PRPF8	9	88	9	4	39.331	cell organization and biogenesis;metabolic process;response to stimulus
P25789	Proteasome subunit alpha type-4 [OS=Homo sapiens]	PSMA4	4	44	4	18	16.736	metabolic process;regulation of biological process;response to stimulus;transport
P28070	Proteasome subunit beta type-4 [OS=Homo sapiens]	PSMB4	3	27	3	15	10.841	metabolic process;regulation of biological process;response to stimulus;transport
P62191	26S proteasome regulatory subunit 4 [OS=Homo sapiens]	PSMC1	6	80	5	19	27.305	metabolic process;regulation of biological process;response to stimulus;transport
P17980	26S proteasome regulatory subunit 6A [OS=Homo sapiens]	PSMC3	10	102	10	28	41.944	metabolic process;regulation of biological process;response to stimulus;transport
P43686	26S proteasome regulatory subunit 6B [OS=Homo sapiens]	PSMC4	6	48	6	17	25.599	metabolic process;regulation of biological process;response to stimulus;transport
P62333	26S proteasome regulatory subunit 10B [OS=Homo sapiens]	PSMC6	3	38	3	10	14.904	cell organization and biogenesis;metabolic process;regulation of biological process;response to stimulus;transport
O00231	26S proteasome non-ATPase regulatory subunit 11 [OS=Homo sapiens]	PSMD11	5	33	5	15	19.531	cell differentiation;cell organization and biogenesis;metabolic process;regulation of biological process;response to stimulus;transport
Q9UNM6	26S proteasome non-ATPase regulatory subunit 13 [OS=Homo sapiens]	PSMD13	4	34	4	9	11.47	cell organization and biogenesis;metabolic process;regulation of biological process;response to stimulus;transport
O00487	26S proteasome non-ATPase regulatory subunit 14 [OS=Homo sapiens]	PSMD14	4	24	4	20	11.895	metabolic process;regulation of biological process;response to stimulus;transport

Q13200	26S proteasome non-ATPase regulatory subunit 2 [OS=Homo sapiens]	PSMD2	8	67	8	10	39.613	metabolic process;regulation of biological process;response to stimulus;transport
P55036-1	26S proteasome non-ATPase regulatory subunit 4 [OS=Homo sapiens]	PSMD4	3	22	3	13	8.88	cell organization and biogenesis;metabolic process;regulation of biological process;response to stimulus;transport
Q16401	26S proteasome non-ATPase regulatory subunit 5 [OS=Homo sapiens]	PSMD5	2	15	2	5	6.049	cell organization and biogenesis;metabolic process;regulation of biological process;response to stimulus;transport
Q15008	26S proteasome non-ATPase regulatory subunit 6 [OS=Homo sapiens]	PSMD6	3	18	3	9	10.342	metabolic process;regulation of biological process;response to stimulus;transport
Q92530	proteasome inhibitor PI31 subunit [OS=Homo sapiens]	PSMF1	3	12	3	15	9.965	metabolic process;regulation of biological process;response to stimulus;transport
Q6NZI2-1	caveolae-associated protein 1 [OS=Homo sapiens]	PTRF; CAVIN1	4	28	4	16	16.132	metabolic process;regulation of biological process
Q9Y3E5	Peptidyl-tRNA hydrolase 2, mitochondrial [OS=Homo sapiens]	PTRH2	2	27	2	19	11.23	cell death;regulation of biological process
Q9UHX1-1	poly(U)-binding-splicing factor PUF60 [OS=Homo sapiens]	PUF60	2	17	2	8	8.244	cell death;cellular homeostasis;metabolic process;regulation of biological process
P47897	glutamine--tRNA ligase [OS=Homo sapiens]	QARS	7	60	7	12	25.47	metabolic process;regulation of biological process
P61026	ras-related protein rab-10 [OS=Homo sapiens]	RAB10	3	72	1	17	19.93	cell differentiation;cell organization and biogenesis;regulation of biological process;response to stimulus;transport
P61019-1	Ras-related protein Rab-2A [OS=Homo sapiens]	RAB2A	4	55	4	25	21.786	cell organization and biogenesis;metabolic process;regulation of biological process;response to stimulus;transport

P51148	Ras-related protein Rab-5C [OS=Homo sapiens]	RAB5C	5	80	4	35	30.094	regulation of biological process;response to stimulus;transport
Q92878	DNA repair protein Rad50 [OS=Homo sapiens]	RAD50	5	40	5	4	14.101	cell organization and biogenesis;metabolic process;regulation of biological process;response to stimulus
P11233	Ras-related protein Ral-A [OS=Homo sapiens]	RALA	3	33	3	11	13.875	cell division;cell organization and biogenesis;regulation of biological process;response to stimulus;transport
P46060	Ran GTPase-activating protein 1 [OS=Homo sapiens]	RANGAP 1	2	29	2	4	10.147	cell organization and biogenesis;metabolic process;regulation of biological process;response to stimulus
P61225	ras-related protein Rap-2b [OS=Homo sapiens]	RAP2B	2	41	2	13	10.78	cell organization and biogenesis;regulation of biological process;response to stimulus;transport
Q96PK6-1	RNA-binding protein 14 [OS=Homo sapiens]	RBM14; RBM14-RBM4	2	21	2	5	6.87	cell organization and biogenesis;defense response;metabolic process;regulation of biological process;response to stimulus
P84098	60S ribosomal protein L19 [OS=Homo sapiens]	RPL19	2	28	2	13	9.714	cell organization and biogenesis;metabolic process;transport
P46778	60S ribosomal protein L21 [OS=Homo sapiens]	RPL21	3	32	3	28	15.186	cell organization and biogenesis;metabolic process;transport
P62910	60S ribosomal protein L32 [OS=Homo sapiens]	RPL32	5	39	5	42	19.528	cell organization and biogenesis;metabolic process;response to stimulus;transport
P18077	60S ribosomal protein L35a [OS=Homo sapiens]	RPL35A	3	17	3	14	13.336	cell organization and biogenesis;metabolic process;transport
P63173	60s ribosomal protein l38 [OS=Homo sapiens]	RPL38	4	29	4	50	14.067	cell organization and biogenesis;metabolic process;regulation of biological process;transport

Q9NQG5	regulation of nuclear pre-mRNA domain-containing protein 1B [OS=Homo sapiens]	RPRD1B	3	36	3	14	13.411	metabolic process;regulation of biological process
P08708	40S ribosomal protein S17 [OS=Homo sapiens]	RPS17; RPS17L	3	27	3	40	10.947	cell organization and biogenesis;metabolic process;transport
P62979	Ubiquitin-40S ribosomal protein S27a [OS=Homo sapiens]	RPS27A	9	550	2	60	71.201	cell communication;cell organization and biogenesis;cellular homeostasis;defense response;metabolic process;regulation of biological process;response to stimulus;transport
P46782	40S ribosomal protein S5 [OS=Homo sapiens]	RPS5	4	63	4	27	32.472	cell organization and biogenesis;metabolic process;regulation of biological process;transport
O76021	Ribosomal L1 domain-containing protein 1 [OS=Homo sapiens]	RSL1D1	4	28	4	11	13.41	cell differentiation;metabolic process;regulation of biological process
Q9Y265	RuvB-like 1 [OS=Homo sapiens]	RUVBL1	4	35	4	13	24.299	cell division;cell organization and biogenesis;metabolic process;regulation of biological process;response to stimulus
P60903	Protein S100-A10 [OS=Homo sapiens]	S100A10	2	35	2	28	10.82	cell organization and biogenesis;regulation of biological process;response to stimulus
O75396	Vesicle-trafficking protein SEC22b [OS=Homo sapiens]	SEC22B	3	38	3	21	17.174	cell organization and biogenesis;regulation of biological process;transport
Q15436	protein transport protein Sec23A [OS=Homo sapiens]	SEC23A	9	109	9	16	46.715	cell organization and biogenesis;transport
P53992	Protein transport protein sec24c [OS=Homo sapiens]	SEC24C	5	29	5	6	20.303	cell organization and biogenesis;regulation of biological process;transport
P61619	Protein transport protein Sec61 subunit alpha isoform 1 [OS=Homo sapiens]	SEC61A1	3	39	3	5	14.945	cell growth;cell organization and biogenesis;response to stimulus;transport

Q9UHD8-1	Septin-9 [OS=Homo sapiens]	SEPT9	5	55	4	9	24.754	cell division;cell organization and biogenesis;regulation of biological process
O75533-1	splicing factor 3B subunit 1 [OS=Homo sapiens]	SF3B1	5	58	5	4	20.06	cell organization and biogenesis;metabolic process;regulation of biological process;response to stimulus
Q15393-1	Splicing factor 3B subunit 3 [OS=Homo sapiens]	SF3B3	2	27	2	2	10.51	cell organization and biogenesis;metabolic process;regulation of biological process
P23246-1	splicing factor, proline- and glutamine-rich [OS=Homo sapiens]	SFPQ	5	33	5	9	22.56	cell organization and biogenesis;defense response;metabolic process;regulation of biological process;response to stimulus
Q6NUK1	Calcium-binding mitochondrial carrier protein SCaMC-1 [OS=Homo sapiens]	SLC25A24	4	32	4	9	16.206	regulation of biological process;response to stimulus;transport
O95347-1	structural maintenance of chromosomes protein 2 [OS=Homo sapiens]	SMC2	2	16	2	2	6.309	cell division;cell organization and biogenesis;metabolic process;regulation of biological process
O75643-1	U5 small nuclear ribonucleoprotein 200 kDa helicase [OS=Homo sapiens]	SNRNP200	8	94	8	5	40.716	cell differentiation;cell organization and biogenesis;metabolic process
Q13596	Sorting nexin-1 [OS=Homo sapiens]	SNX1	2	26	1	3	10.142	cell organization and biogenesis;metabolic process;regulation of biological process;transport
Q9UNH7-1	Sorting nexin-6 [OS=Homo sapiens]	SNX6	3	17	3	9	11.681	cell organization and biogenesis;regulation of biological process;transport
Q13813	Spectrin alpha chain, non-erythrocytic 1 [OS=Homo sapiens]	SPTAN1	7	44	7	4	32.09	cell organization and biogenesis;cellular component movement;metabolic process;regulation of biological process;response to stimulus;transport
Q9Y6N5	Sulfide:quinone oxidoreductase, mitochondrial [OS=Homo sapiens]	SQRDL; SQOR	6	61	6	14	29.073	metabolic process

P19623	spermidine synthase [OS=Homo sapiens]	SRM	5	60	5	20	19.628	metabolic process
P37108	Signal recognition particle 14 kDa protein [OS=Homo sapiens]	SRP14	4	24	4	40	31.032	cell organization and biogenesis;response to stimulus;transport
Q9UHB9	Signal recognition particle subunit SRP68 [OS=Homo sapiens]	SRP68	3	63	3	6	15.869	cell organization and biogenesis;response to stimulus;transport
Q08945	FACT complex subunit SSRP1 [OS=Homo sapiens]	SSRP1	4	41	4	7	22.872	metabolic process;regulation of biological process;response to stimulus
P31948	stress-induced-phosphoprotein 1 [OS=Homo sapiens]	STIP1	6	61	6	14	27.677	response to stimulus
Q8TCJ2	Dolichyl-diphosphooligosaccharide--protein glycosyltransferase subunit STT3B [OS=Homo sapiens]	STT3B	4	36	4	5	15.307	metabolic process;response to stimulus
P53597	Succinate--CoA ligase [ADP/GDP-forming] subunit alpha, mitochondrial [OS=Homo sapiens]	SUCLG1	2	36	2	9	9.71	metabolic process
Q96I99-1	Succinate--CoA ligase [GDP-forming] subunit beta, mitochondrial [OS=Homo sapiens]	SUCLG2	3	22	3	11	14.595	metabolic process
Q9Y5B9	FACT complex subunit SPT16 [OS=Homo sapiens]	SUPT16H	7	68	7	8	28.818	cell organization and biogenesis;metabolic process;regulation of biological process;response to stimulus
Q9UH65	Switch-associated protein 70 [OS=Homo sapiens]	SWAP70	2	31	2	3	8.855	cell organization and biogenesis;cellular homeostasis;metabolic process;regulation of biological process
O60506	Heterogeneous nuclear ribonucleoprotein Q [OS=Homo sapiens]	SYNCRIP	8	101	5	15	31.724	cell differentiation;metabolic process;regulation of biological process;response to stimulus
P26639-1	Threonine--tRNA ligase, cytoplasmic [OS=Homo sapiens]	TARS	8	71	8	14	35.619	metabolic process
P07996	thrombospondin-1 [OS=Homo sapiens]	THBS1	4	22	4	5	12.22	cell organization and biogenesis;cellular component

								movement;defense response;metabolic process;regulation of biological process;response to stimulus;transport
Q92544	Transmembrane 9 superfamily member 4 [OS=Homo sapiens]	TM9SF4	2	16	2	4	7.315	cell organization and biogenesis;cellular homeostasis;regulation of biological process;response to stimulus;transport
Q13445	Transmembrane emp24 domain-containing protein 1 [OS=Homo sapiens]	TMED1	2	14	2	12	13.983	cell communication;regulation of biological process;response to stimulus;transport
Q92973-1	transportin-1 [OS=Homo sapiens]	TNPO1	9	71	9	12	38.835	cellular component movement;regulation of biological process;transport
P11387	DNA topoisomerase 1 [OS=Homo sapiens]	TOP1	6	92	6	8	31.84	cell death;cell division;cell organization and biogenesis;metabolic process;regulation of biological process;response to stimulus
Q14258	E3 ubiquitin/ISG15 ligase TRIM25 [OS=Homo sapiens]	TRIM25	5	48	5	9	25.203	defense response;metabolic process;regulation of biological process;response to stimulus
Q13263	Transcription intermediary factor 1-beta [OS=Homo sapiens]	TRIM28	8	83	8	15	34.512	cell organization and biogenesis;defense response;metabolic process;regulation of biological process;response to stimulus
Q13509	tubulin beta-3 chain [OS=Homo sapiens]	TUBB3	19	2010	2	45	264.623	cell organization and biogenesis;cellular component movement
P04350	Tubulin beta-4A chain [OS=Homo sapiens]	TUBB4A	26	2013	3	69	293.924	cell organization and biogenesis;regulation of biological process
Q12792-2	Twinfilin-1 [OS=Homo sapiens]	TWF1	3	27	3	11	12.988	cell organization and biogenesis;regulation of biological process
Q6IBS0	Twinfilin-2 [OS=Homo sapiens]	TWF2	2	36	2	10	17.497	cell organization and biogenesis;regulation of biological process;response to stimulus

Q16222-1	UDP-N-acetylhexosamine pyrophosphorylase [OS=Homo sapiens]	UAP1	3	60	3	7	16.691	metabolic process
Q9UBT2	SUMO-activating enzyme subunit 2 [OS=Homo sapiens]	UBA2	6	46	6	12	25.263	metabolic process;regulation of biological process
Q13404	Ubiquitin-conjugating enzyme E2 variant 1 [OS=Homo sapiens]	UBE2V1; TMEM189-UBE2V1	4	20	4	27	12.783	cell differentiation;metabolic process;regulation of biological process;response to stimulus
Q9H3U1	Protein unc-45 homolog A [OS=Homo sapiens]	UNC45A	5	28	5	7	23.91	cell differentiation;development;metabolic process
P22695	Cytochrome b-c1 complex subunit 2, mitochondrial [OS=Homo sapiens]	UQCRC2	9	96	9	26	47.904	metabolic process;transport
O60763-1	General vesicular transport factor p115 [OS=Homo sapiens]	USO1	6	69	6	9	25.172	cell organization and biogenesis;metabolic process;transport
Q14694	Ubiquitin carboxyl-terminal hydrolase 10 [OS=Homo sapiens]	USP10	2	20	2	3	8.064	metabolic process;regulation of biological process;response to stimulus
P45974-1	Ubiquitin carboxyl-terminal hydrolase 5 [OS=Homo sapiens]	USP5	8	51	8	14	33.21	metabolic process;regulation of biological process
Q93008-1	Isoform 2 of Probable ubiquitin carboxyl-terminal hydrolase FAF-X [OS=Homo sapiens]	USP9X	4	18	4	2	11.548	cell division;cell growth;cellular component movement;metabolic process;regulation of biological process;response to stimulus
P26640	Valine--tRNA ligase [OS=Homo sapiens]	VARS; VARS2	6	24	6	7	20.586	metabolic process;regulation of biological process
P18206	Vinculin [OS=Homo sapiens]	VCL	13	111	13	14	61.836	cell growth;cell organization and biogenesis;cellular component movement;regulation of biological process;transport
Q9Y277	Voltage-dependent anion-selective channel protein 3 [OS=Homo sapiens]	VDAC3	3	24	2	12	15.252	cell communication;defense response;regulation of biological process;response to stimulus;transport
O75436-1	Vacuolar protein sorting-associated protein 26A [OS=Homo sapiens]	VPS26A	2	15	2	9	7.735	regulation of biological process;response to stimulus;transport

Q96QK1	Vacuolar protein sorting-associated protein 35 [OS=Homo sapiens]	VPS35	8	83	8	10	30.047	cell organization and biogenesis;regulation of biological process;response to stimulus;transport
P23381	Tryptophan--tRNA ligase, cytoplasmic [OS=Homo sapiens]	WARS	3	28	3	10	11.966	metabolic process;regulation of biological process
O14980	Exportin-1 [OS=Homo sapiens]	XPO1	10	103	10	11	46.747	cell organization and biogenesis;regulation of biological process;response to stimulus;transport
Q9UIA9	exportin-7 [OS=Homo sapiens]	XPO7	4	40	4	4	14.299	transport
O43592	Exportin-T [OS=Homo sapiens]	XPOT	3	20	3	4	11.284	transport
P31946	14-3-3 protein beta/alpha [OS=Homo sapiens]	YWHAB	5	163	2	27	21.855	cell organization and biogenesis;metabolic process;regulation of biological process;response to stimulus;transport
Q04917	14-3-3 protein eta [OS=Homo sapiens]	YWHAH	7	190	5	25	41.44	cell organization and biogenesis;metabolic process;regulation of biological process;response to stimulus;transport

Table S2: List of 394 proteins modified by 2-ClHDyA in EA.hy926 cells including those also modified by HDyA

Accession	Description	Gene Symbol	# Peptides	# PSMs	# Unique Peptides	Coverage [%]	Sum PEP Score	Biological Process
P61221	ATP-binding cassette sub-family E member 1 [OS=Homo sapiens]	ABCE1	6	44	6	13	21.202	cell organization and biogenesis;metabolic process;regulation of biological process;transport
P09110-1	3-ketoacyl-CoA thiolase, peroxisomal [OS=Homo sapiens]	ACAA1	10	131	10	38	60.632	metabolic process;transport
P42765	3-ketoacyl-CoA thiolase, mitochondrial [OS=Homo sapiens]	ACAA2	6	42	6	28	22.725	cell organization and biogenesis;metabolic process;regulation of biological process;response to stimulus
Q13085-1	Acetyl-CoA carboxylase 1 [OS=Homo sapiens]	ACACA	6	41	6	3	24.463	cell organization and biogenesis;metabolic process;regulation of biological process;response to stimulus;transport
P24752	Acetyl-CoA acetyltransferase, mitochondrial [OS=Homo sapiens]	ACAT1	13	130	13	43	61.3	cell organization and biogenesis;metabolic process;response to stimulus
Q99798	Aconitate hydratase, mitochondrial [OS=Homo sapiens]	ACO2	5	18	5	10	17.453	metabolic process;response to stimulus
O00154-4	Isoform 4 of Cytosolic acyl coenzyme A thioester hydrolase [OS=Homo sapiens]	ACOT7	7	102	7	29	32.112	metabolic process
Q9Y305-1	Acyl-coenzyme A thioesterase 9, mitochondrial [OS=Homo sapiens]	ACOT9	4	30	4	10	14.675	metabolic process
O95573	long-chain-fatty-acid--CoA ligase 3 [OS=Homo sapiens]	ACSL3	5	46	3	10	21.683	cell organization and biogenesis;metabolic process;regulation of biological process;response to stimulus;transport
O60488-1	Long-chain-fatty-acid--CoA ligase 4 [OS=Homo sapiens]	ACSL4	6	44	4	12	24.194	cell differentiation;metabolic process;regulation of biological

								process;response to stimulus;transport
P12814-1	Alpha-actinin-1 [OS=Homo sapiens]	ACTN1	30	600	18	42	171.711	cell differentiation;cell organization and biogenesis;regulation of biological process;transport
O43707	Alpha-actinin-4 [OS=Homo sapiens]	ACTN4	27	647	15	38	167.997	cell organization and biogenesis;cellular component movement;regulation of biological process;response to stimulus;transport
P61163	Alpha-centractin [OS=Homo sapiens]	ACTR1 A	5	62	5	24	26.894	regulation of biological process;transport
P61160-1	Actin-related protein 2 [OS=Homo sapiens]	ACTR2	7	79	7	22	39.621	cell division;cell organization and biogenesis;cellular component movement;regulation of biological process;response to stimulus;transport
P61158	actin-related protein 3 [OS=Homo sapiens]	ACTR3	8	81	8	29	45.111	cell division;cell organization and biogenesis;cellular component movement;regulation of biological process;response to stimulus
P37023	Serine/threonine-protein kinase receptor R3 [OS=Homo sapiens]	ACVRL 1	2	16	2	6	8.395	cell differentiation;cell organization and biogenesis;cell proliferation;cellular component movement;metabolic process;regulation of biological process;response to stimulus
P30520	adenylosuccinate synthetase isozyme 2 [OS=Homo sapiens]	ADSS	4	54	4	13	26.475	metabolic process;response to stimulus
O00116	Alkyldihydroxyacetonephosphate synthase, peroxisomal [OS=Homo sapiens]	AGPS	3	47	3	6	16.795	metabolic process
Q13155	aminoacyl tRNA synthase complex-interacting multifunctional protein 2 [OS=Homo sapiens]	AIMP2	3	21	3	17	12.218	cell death;cell differentiation;cell organization and biogenesis;development;metabolic process;regulation of biological process
P00568	Adenylate kinase isoenzyme 1 [OS=Homo sapiens]	AK1	2	31	2	13	7.818	metabolic process
P15121	aldose reductase [OS=Homo sapiens]	AKR1B1	5	92	4	11	25.171	metabolic process;regulation of biological process;response to stimulus

P54886	delta-1-pyrroline-5-carboxylate synthase [OS=Homo sapiens]	ALDH18A1	7	106	7	10	38.77	metabolic process
P00352	Retinal dehydrogenase 1 [OS=Homo sapiens]	ALDH1A1	15	237	14	36	85.926	metabolic process;regulation of biological process
P49419	Alpha-aminoadipic semialdehyde dehydrogenase [OS=Homo sapiens]	ALDH7A1	5	25	5	15	17.78	metabolic process
P04083	annexin A1 [OS=Homo sapiens]	ANXA1	10	69	10	37	41.926	cell differentiation;cell organization and biogenesis;cellular component movement;defense response;metabolic process;regulation of biological process;response to stimulus;transport
P08133-1	annexin A6 [OS=Homo sapiens]	ANXA6	4	31	4	7	13.608	cell organization and biogenesis;cellular homeostasis;regulation of biological process;response to stimulus;transport
P20073-1	Annexin A7 [OS=Homo sapiens]	ANXA7	4	48	4	10	20.365	cell differentiation;cell organization and biogenesis;cell proliferation;cellular homeostasis;metabolic process;regulation of biological process;response to stimulus
Q9BXS5-1	AP-1 complex subunit mu-1 [OS=Homo sapiens]	AP1M1	5	20	5	17	15.492	cell organization and biogenesis;regulation of biological process;transport
O95782	AP-2 complex subunit alpha-1 [OS=Homo sapiens]	AP2A1	8	94	3	11	31.456	cell organization and biogenesis;cellular component movement;metabolic process;regulation of biological process;response to stimulus;transport
O94973	AP-2 complex subunit alpha-2 [OS=Homo sapiens]	AP2A2	9	71	4	13	30.102	cell organization and biogenesis;cellular component movement;metabolic process;regulation of biological process;response to stimulus;transport
P63010-1	AP-2 complex subunit beta [OS=Homo sapiens]	AP2B1	7	101	7	8	30.303	cell organization and biogenesis;cellular component

								movement;metabolic process;regulation of biological process;response to stimulus;transport
Q96CW1	AP-2 complex subunit mu [OS=Homo sapiens]	AP2M1	5	49	5	14	26.689	cell organization and biogenesis;cellular component movement;metabolic process;regulation of biological process;response to stimulus;transport
O00203-1	AP-3 complex subunit beta-1 [OS=Homo sapiens]	AP3B1	2	22	2	3	7.489	cell organization and biogenesis;cellular component movement;coagulation;regulation of biological process;transport
Q9Y2T2	ap-3 complex subunit mu-1 [OS=Homo sapiens]	AP3M1	2	23	2	6	7.702	cellular component movement;transport
P48444	Coatomer subunit delta [OS=Homo sapiens]	ARCN1	6	125	6	11	32.866	response to stimulus;transport
P61204	ADP-ribosylation factor 3 [OS=Homo sapiens]	ARF3	7	86	4	51	42.394	metabolic process;regulation of biological process;response to stimulus;transport
P18085	ADP-ribosylation factor 4 [OS=Homo sapiens]	ARF4	6	49	3	41	27.618	cellular component movement;metabolic process;regulation of biological process;response to stimulus;transport
P62330	ADP-ribosylation factor 6 [OS=Homo sapiens]	ARF6	2	37	2	12	10.531	cell death;cell differentiation;cell division;cell organization and biogenesis;cellular component movement;regulation of biological process;response to stimulus;transport
Q8N392-1	Rho GTPase-activating protein 18 [OS=Homo sapiens]	ARHGAP18	3	17	3	5	11.019	cell organization and biogenesis;regulation of biological process;response to stimulus
P40616	ADP-ribosylation factor-like protein 1 [OS=Homo sapiens]	ARL1	3	46	3	19	16.567	cell organization and biogenesis;metabolic process;regulation of biological process;response to stimulus;transport

O15143	Actin-related protein 2/3 complex subunit 1B [OS=Homo sapiens]	ARPC1B	3	45	3	11	16.043	cell organization and biogenesis;cellular component movement;regulation of biological process;response to stimulus
O15144	Actin-related protein 2/3 complex subunit 2 [OS=Homo sapiens]	ARPC2	4	41	4	12	14.602	cell organization and biogenesis;cellular component movement;regulation of biological process;response to stimulus
Q6DD88	Atlastin-3 [OS=Homo sapiens]	ATL3	4	66	4	11	27.694	cell organization and biogenesis;regulation of biological process;transport
P48047	ATP synthase subunit O, mitochondrial [OS=Homo sapiens]	ATP5O	4	84	4	20	15.389	cell organization and biogenesis;metabolic process;transport
P21281	V-type proton ATPase subunit B, brain isoform [OS=Homo sapiens]	ATP6V1B2	4	47	4	12	14.648	cellular homeostasis;metabolic process;regulation of biological process;response to stimulus;transport
O43684	Mitotic checkpoint protein BUB3 [OS=Homo sapiens]	BUB3	4	39	4	13	17.118	cell division;cell organization and biogenesis;metabolic process;regulation of biological process
Q9NQ88	Fructose-2,6-bisphosphatase TIGAR [OS=Homo sapiens]	C12orf5; TIGAR	2	19	2	10	8.744	cell death;metabolic process;regulation of biological process;response to stimulus
Q9Y3I0	tRNA-splicing ligase RtcB homolog [OS=Homo sapiens]	C22orf28; RTCB	7	78	7	16	39.425	metabolic process
Q13557	Calcium/calmodulin-dependent protein kinase type II subunit delta [OS=Homo sapiens]	CAMK2D	3	41	3	7	14.872	cell differentiation;cell organization and biogenesis;cellular component movement;cellular homeostasis;metabolic process;regulation of biological process;response to stimulus
Q86VP6-1	cullin-associated nedd8-dissociated protein 1 [OS=Homo sapiens]	CAND1	27	356	27	25	145.672	cell differentiation;cell organization and biogenesis;cellular homeostasis;metabolic process;regulation of biological process;transport
P27824	Calnexin [OS=Homo sapiens]	CANX	4	27	4	8	18.981	metabolic process;regulation of biological process;response to stimulus;transport

P07384	Calpain-1 catalytic subunit [OS=Homo sapiens]	CAPN1	4	51	4	7	22.1	cell death;cell organization and biogenesis;metabolic process;regulation of biological process;transport
P52907	F-actin-capping protein subunit alpha-1 [OS=Homo sapiens]	CAPZA 1	3	46	2	14	13.776	cell organization and biogenesis;cellular component movement;coagulation;defense response;regulation of biological process;response to stimulus;transport
P47755	F-actin-capping protein subunit alpha-2 [OS=Homo sapiens]	CAPZA 2	3	53	2	14	18.233	cell organization and biogenesis;cellular component movement;coagulation;defense response;regulation of biological process;response to stimulus;transport
Q86X55	Histone-arginine methyltransferase CARM1 [OS=Homo sapiens]	CARM1	3	39	3	10	15.348	cell organization and biogenesis;metabolic process;regulation of biological process;response to stimulus
P04040	catalase [OS=Homo sapiens]	CAT	3	22	3	9	16.407	cell differentiation;cell organization and biogenesis;metabolic process;regulation of biological process;response to stimulus;transport
Q03135	Caveolin-1 [OS=Homo sapiens]	CAV1	3	157	3	25	22.318	cell communication;cell differentiation;cell organization and biogenesis;cellular component movement;cellular homeostasis;metabolic process;regulation of biological process;response to stimulus;transport
P16152	Carbonyl reductase [NADPH] 1 [OS=Homo sapiens]	CBR1; SETD4	7	133	7	41	39.257	cell differentiation;metabolic process
P48643	T-complex protein 1 subunit epsilon [OS=Homo sapiens]	CCT5	13	181	13	29	56.303	metabolic process;regulation of biological process;response to stimulus;transport
P21926	CD9 antigen [OS=Homo sapiens]	CD9	2	72	2	4	15.598	cell organization and biogenesis;cellular component movement;metabolic process;regulation of biological

								process;response to stimulus;transport
Q16543	Hsp90 co-chaperone Cdc37 [OS=Homo sapiens]	CDC37	4	42	4	14	19.443	cell division;metabolic process;regulation of biological process;response to stimulus;transport
Q00535	Cyclin-dependent-like kinase 5 [OS=Homo sapiens]	CDK5	3	18	3	9	12.872	cell communication;cell death;cell differentiation;cell division;cell growth;cell organization and biogenesis;cell proliferation;cellular component movement;metabolic process;regulation of biological process;response to stimulus;transport
O00299	chloride intracellular channel protein 1 [OS=Homo sapiens]	CLIC1	7	124	7	45	47.443	metabolic process;regulation of biological process;response to stimulus;transport
Q9Y696	Chloride intracellular channel protein 4 [OS=Homo sapiens]	CLIC4	8	46	8	43	27.706	cell differentiation;cell organization and biogenesis;cellular homeostasis;metabolic process;regulation of biological process;response to stimulus;transport
Q96KP4	cytosolic non-specific dipeptidase [OS=Homo sapiens]	CNDP2	3	15	3	10	8.701	metabolic process
Q8NBJ5	Procollagen galactosyltransferase 1 [OS=Homo sapiens]	COLGALT1; GLT25D1	5	38	5	9	21.911	
P21964-1	Catechol O-methyltransferase [OS=Homo sapiens]	COMT	4	35	4	20	22.626	cell communication;metabolic process;regulation of biological process;response to stimulus
P53618	Coatomer subunit beta [OS=Homo sapiens]	COPB1	14	133	14	19	65.74	transport
P35606	Coatomer subunit beta' [OS=Homo sapiens]	COPB2	11	127	11	16	49.446	transport
Q9Y678	Coatomer subunit gamma-1 [OS=Homo sapiens]	COPG1	17	285	17	28	108.994	cellular component movement;transport
P61923	Coatomer subunit zeta-1 [OS=Homo sapiens]	COPZ1	3	30	3	27	14.817	transport

Q99829	Copine-1 [OS=Homo sapiens]	CPNE1	4	42	4	7	17.563	cell differentiation;cell growth;metabolic process;regulation of biological process;response to stimulus;transport
P36551	Oxygen-dependent coproporphyrinogen-III oxidase, mitochondrial [OS=Homo sapiens]	CPOX	4	36	4	12	18.099	metabolic process;response to stimulus
P52943	Cysteine-rich protein 2 [OS=Homo sapiens]	CRIP2	4	62	4	32	23.822	regulation of biological process
P41240	tyrosine-protein kinase CSK [OS=Homo sapiens]	CSK	3	14	3	8	9.862	cell differentiation;cell organization and biogenesis;defense response;metabolic process;regulation of biological process;response to stimulus
P56545	c-terminal-binding protein 2 [OS=Homo sapiens]	CTBP2	2	25	1	6	9.605	cell differentiation;metabolic process;regulation of biological process
P35222	Catenin beta-1 [OS=Homo sapiens]	CTNNB 1	9	43	6	17	44.974	cell communication;cell differentiation;cell growth;cell organization and biogenesis;cell proliferation;cellular component movement;metabolic process;regulation of biological process;response to stimulus;transport
O60716-1	Catenin delta-1 [OS=Homo sapiens]	CTNND 1	12	141	12	18	56.114	cell organization and biogenesis;metabolic process;regulation of biological process;response to stimulus
O43169	Cytochrome b5 type B [OS=Homo sapiens]	CYB5B	3	49	3	45	24.678	metabolic process;transport
Q7L576-1	cytoplasmic FMR1-interacting protein 1 [OS=Homo sapiens]	CYFIP1	8	85	8	7	43.014	cell differentiation;cell growth;cell organization and biogenesis;development;regulation of biological process;response to stimulus;transport
P61803	Dolichyl-diphosphooligosaccharide--protein glycosyltransferase	DAD1	3	43	3	29	15.652	cell death;metabolic process;regulation of biological process;response to stimulus

	subunit DAD1 [OS=Homo sapiens]							
P14868	Aspartate--tRNA ligase, cytoplasmic [OS=Homo sapiens]	DARS	12	114	12	28	45.131	cell organization and biogenesis;metabolic process
Q6PI48	Aspartate--tRNA ligase, mitochondrial [OS=Homo sapiens]	DARS2	3	37	3	6	13.475	metabolic process
Q92499	ATP-dependent RNA helicase DDX1 [OS=Homo sapiens]	DDX1	7	92	7	10	34.476	cell organization and biogenesis;development;metabolic process;regulation of biological process;response to stimulus
Q92841-1	Isoform 2 of Probable ATP-dependent RNA helicase DDX17 [OS=Homo sapiens]	DDX17	18	394	12	27	104.908	cell differentiation;cell organization and biogenesis;defense response;metabolic process;regulation of biological process;response to stimulus
Q9NR30-1	Nucleolar RNA helicase 2 [OS=Homo sapiens]	DDX21	6	42	6	9	21.475	cell differentiation;metabolic process;regulation of biological process;response to stimulus
O00148	ATP-dependent RNA helicase DDX39A [OS=Homo sapiens]	DDX39A	9	184	2	23	48.632	metabolic process;regulation of biological process;response to stimulus;transport
Q13838-1	spliceosome RNA helicase DDX39B [OS=Homo sapiens]	DDX39B	10	183	3	23	51.709	cell organization and biogenesis;metabolic process;regulation of biological process;transport
O00571	ATP-dependent RNA helicase DDX3X [OS=Homo sapiens]	DDX3X	10	210	8	16	62.417	cell death;cell organization and biogenesis;defense response;metabolic process;regulation of biological process;response to stimulus;transport
Q86XP3-1	ATP-dependent RNA helicase DDX42 [OS=Homo sapiens]	DDX42	3	22	3	3	10.88	metabolic process;regulation of biological process
Q7L014	probable ATP-dependent RNA helicase DDX46 [OS=Homo sapiens]	DDX46	8	106	8	10	36.291	metabolic process;regulation of biological process
P26196	Probable ATP-dependent RNA helicase DDX6 [OS=Homo sapiens]	DDX6	7	72	7	20	32.456	cell organization and biogenesis;metabolic process;regulation of biological process

P23743-1	diacylglycerol kinase alpha [OS=Homo sapiens]	DGKA	3	12	3	6	9.986	metabolic process;regulation of biological process;response to stimulus
O43143	Pre-mRNA-splicing factor ATP-dependent RNA helicase DHX15 [OS=Homo sapiens]	DHX15	8	82	8	11	33.94	metabolic process;response to stimulus
P09622	Dihydrolipoyl dehydrogenase, mitochondrial [OS=Homo sapiens]	DLD	4	42	4	13	15.742	cellular homeostasis;metabolic process;regulation of biological process
P36957	Dihydrolipoyllysine-residue succinyltransferase component of 2-oxoglutarate dehydrogenase complex, mitochondrial [OS=Homo sapiens]	DLST	3	56	3	8	22.62	metabolic process
Q9UDY4	DnaJ homolog subfamily B member 4 [OS=Homo sapiens]	DNAJB4	4	44	3	17	14.628	metabolic process;regulation of biological process;response to stimulus
Q8IXB1-1	DnaJ homolog subfamily C member 10 [OS=Homo sapiens]	DNAJC 10	5	38	4	6	15.207	cellular homeostasis;metabolic process;regulation of biological process;response to stimulus
O00429	Dynamin-1-like protein [OS=Homo sapiens]	DNM1L	9	68	9	16	31.972	cell death;cell organization and biogenesis;regulation of biological process;response to stimulus;transport
P50570-1	Dynamin-2 [OS=Homo sapiens]	DNM2	7	115	7	9	26.088	cell organization and biogenesis;metabolic process;regulation of biological process;response to stimulus;transport
Q9Y295	developmentally-regulated GTP-binding protein 1 [OS=Homo sapiens]	DRG1	6	65	6	23	29.759	development;metabolic process
O75923-1	dysferlin [OS=Homo sapiens]	DYSF	3	20	3	2	13.4	cell organization and biogenesis
Q12805	EGF-containing fibulin-like extracellular matrix protein 1 [OS=Homo sapiens]	EFEMP 1	7	74	7	17	26.956	metabolic process;regulation of biological process;response to stimulus
Q15029	116 kDa U5 small nuclear ribonucleoprotein component [OS=Homo sapiens]	EFTUD 2	2	55	1	2	9.595	cell organization and biogenesis;metabolic process;response to stimulus
Q9H4M9	EH domain-containing protein 1 [OS=Homo sapiens]	EHD1	14	212	13	41	81.472	cell organization and biogenesis;coagulation;regulation of

								biological process;response to stimulus;transport
Q9NZN4	EH domain-containing protein 2 [OS=Homo sapiens]	EHD2	6	49	5	12	28.195	cell organization and biogenesis;coagulation;regulation of biological process;transport
Q9H223	EH domain-containing protein 4 [OS=Homo sapiens]	EHD4	7	99	7	16	33.887	cell organization and biogenesis;regulation of biological process;response to stimulus;transport
Q9BY44-1	Eukaryotic translation initiation factor 2A [OS=Homo sapiens]	EIF2A	3	24	3	8	11.168	cell organization and biogenesis;metabolic process;regulation of biological process;response to stimulus
P19525	interferon-induced, double-stranded RNA-activated protein kinase [OS=Homo sapiens]	EIF2AK 2	2	17	2	4	6.913	cell communication;defense response;metabolic process;regulation of biological process;response to stimulus
P41091	eukaryotic translation initiation factor 2 subunit 3 [OS=Homo sapiens]	EIF2S3	8	94	8	26	39.684	metabolic process;transport
Q14152	Eukaryotic translation initiation factor 3 subunit A [OS=Homo sapiens]	EIF3A	12	109	12	11	53.257	cell organization and biogenesis;metabolic process;regulation of biological process
B5ME19	eukaryotic translation initiation factor 3 subunit C-like protein [OS=Homo sapiens]	EIF3CL	6	113	6	7	35.695	metabolic process
P60228	Eukaryotic translation initiation factor 3 subunit E [OS=Homo sapiens]	EIF3E	4	44	4	11	17.353	metabolic process;regulation of biological process
O00303	Eukaryotic translation initiation factor 3 subunit F [OS=Homo sapiens]	EIF3F	4	57	4	13	24.747	metabolic process
Q9Y262	eukaryotic translation initiation factor 3 subunit L [OS=Homo sapiens]	EIF3L	4	32	4	10	21.31	metabolic process
Q14240-1	eukaryotic initiation factor 4A-II [OS=Homo sapiens]	EIF4A2	10	354	2	27	79.092	metabolic process;regulation of biological process
P38919	Eukaryotic initiation factor 4A-III [OS=Homo sapiens]	EIF4A3	4	60	3	12	22.959	metabolic process;regulation of biological process;response to stimulus;transport

Q04637	eukaryotic translation initiation factor 4 gamma 1 [OS=Homo sapiens]	EIF4G1	3	16	3	2	13.446	cell organization and biogenesis;defense response;metabolic process;regulation of biological process;response to stimulus
Q6IS14	Eukaryotic translation initiation factor 5A-1-like [OS=Homo sapiens]	EIF5AL1	3	13	3	33	8.843	metabolic process;regulation of biological process;transport
P17813	Endoglin [OS=Homo sapiens]	ENG	7	47	7	13	31.57	cell differentiation;cell organization and biogenesis;cellular component movement;regulation of biological process;response to stimulus;transport
P29317	Ephrin type-A receptor 2 [OS=Homo sapiens]	EPHA2	4	27	4	4	15.174	cell death;cell differentiation;cell organization and biogenesis;cell proliferation;cellular component movement;defense response;development;metabolic process;regulation of biological process;response to stimulus
P07814	Bifunctional glutamate/proline--tRNA ligase [OS=Homo sapiens]	EPRS	14	113	14	13	51.704	cell organization and biogenesis;metabolic process;regulation of biological process;response to stimulus
Q9NZ08-1	endoplasmic reticulum aminopeptidase 1 [OS=Homo sapiens]	ERAP1	7	46	7	8	22.243	cell communication;cell differentiation;metabolic process;regulation of biological process;response to stimulus
P84090	Enhancer of rudimentary homolog [OS=Homo sapiens]	ERH	2	28	2	28	11.73	metabolic process;regulation of biological process
Q9BSJ8	Extended synaptotagmin-1 [OS=Homo sapiens]	ESYT1	7	46	7	8	32.169	metabolic process;transport
A0FGR8-1	Extended synaptotagmin-2 [OS=Homo sapiens]	ESYT2	7	48	7	10	28.763	metabolic process;transport
P62495	Eukaryotic peptide chain release factor subunit 1 [OS=Homo sapiens]	ETF1	5	60	5	14	25.537	cell organization and biogenesis;metabolic process;regulation of biological process
P13804-1	Electron transfer flavoprotein subunit alpha, mitochondrial [OS=Homo sapiens]	ETFA	3	41	3	14	14.415	metabolic process;transport

O95571	Persulfide dioxygenase ETHE1, mitochondrial [OS=Homo sapiens]	ETHE1	2	43	2	12	10.45	metabolic process
P15311	Ezrin [OS=Homo sapiens]	EZR	12	351	3	19	64.669	cell differentiation;cell organization and biogenesis;cellular component movement;metabolic process;regulation of biological process;response to stimulus;transport
Q9NZB2-1	Constitutive coactivator of PPAR-gamma-like protein 1 [OS=Homo sapiens]	FAM120A	6	38	6	8	16.621	
Q96TA1	Niban-like protein 1 [OS=Homo sapiens]	FAM129B	8	106	8	12	36.864	cell differentiation;cellular component movement;metabolic process;regulation of biological process;transport
Q9BRX8-1	Redox-regulatory protein FAM213A [OS=Homo sapiens]	FAM213A	8	170	8	34	41.341	metabolic process;regulation of biological process
Q9NSD9	Phenylalanine--tRNA ligase beta subunit [OS=Homo sapiens]	FARSB	5	60	5	9	19.341	cell organization and biogenesis;metabolic process
P14324	Farnesyl pyrophosphate synthase [OS=Homo sapiens]	FDPS	2	40	2	7	9.005	metabolic process;regulation of biological process
P22570	NADPH:adrenodoxin oxidoreductase, mitochondrial [OS=Homo sapiens]	FDXR	8	82	8	18	37.195	metabolic process;transport
P39748	Flap endonuclease 1 [OS=Homo sapiens]	FEN1	2	25	2	8	9.925	cell organization and biogenesis;cellular homeostasis;metabolic process;regulation of biological process;response to stimulus
P07954	fumarate hydratase, mitochondrial [OS=Homo sapiens]	FH	3	44	3	10	18.554	metabolic process
Q13045-1	protein flightless-1 homolog [OS=Homo sapiens]	FLII	3	50	3	2	15.731	cell organization and biogenesis;development;metabolic process;regulation of biological process
P51570	galactokinase [OS=Homo sapiens]	GALK1	3	29	3	11	11.034	metabolic process
Q14697-1	Neutral alpha-glucosidase AB [OS=Homo sapiens]	GANAB	13	384	13	15	90.669	metabolic process

P22102-1	trifunctional purine biosynthetic protein adenosine-3 [OS=Homo sapiens]	GART	7	63	7	10	32.785	metabolic process;response to stimulus
Q04446	1,4-alpha-glucan-branching enzyme [OS=Homo sapiens]	GBE1	6	47	6	15	27.184	metabolic process
Q92616	eIF-2-alpha kinase activator GCN1 [OS=Homo sapiens]	GCN1L 1; GCN1	15	116	15	6	55.413	cell communication;metabolic process;regulation of biological process;response to stimulus
P50395-1	Rab GDP dissociation inhibitor beta [OS=Homo sapiens]	GDI2	16	187	13	51	67.241	metabolic process;regulation of biological process;response to stimulus;transport
Q06210-1	glutamine--fructose-6-phosphate aminotransferase [isomerizing] 1 [OS=Homo sapiens]	GFPT1	7	48	7	14	24.276	metabolic process;regulation of biological process
P04899-1	guanine nucleotide-binding protein G(i) subunit alpha-2 [OS=Homo sapiens]	GNAI2	7	82	6	30	26.672	cell division;cell proliferation;metabolic process;regulation of biological process;response to stimulus
Q5JWF2-1	Guanine nucleotide-binding protein G(S) subunit alpha isoforms XLas [OS=Homo sapiens]	GNAS	4	80	3	5	21.463	metabolic process;regulation of biological process;response to stimulus
P62873	Guanine nucleotide-binding protein G(I)/G(S)/G(T) subunit beta-1 [OS=Homo sapiens]	GNB1	4	33	4	14	21.165	cell death;cell organization and biogenesis;cell proliferation;cellular homeostasis;metabolic process;regulation of biological process;response to stimulus
P62879	Guanine nucleotide-binding protein G(I)/G(S)/G(T) subunit beta-2 [OS=Homo sapiens]	GNB2	3	42	3	10	21.707	metabolic process;regulation of biological process;response to stimulus
Q9Y2Q3-1	Glutathione S-transferase kappa 1 [OS=Homo sapiens]	GSTK1	3	39	3	20	14.809	cell differentiation;metabolic process
P40939	Trifunctional enzyme subunit alpha, mitochondrial [OS=Homo sapiens]	HADHA	11	180	11	20	71.612	metabolic process;response to stimulus
P55084	Trifunctional enzyme subunit beta, mitochondrial [OS=Homo sapiens]	HADHB	8	69	8	19	29.141	metabolic process
Q00341-1	Vigilin [OS=Homo sapiens]	HDLBP	11	127	11	12	47.817	metabolic process;transport

P19367-1	Hexokinase-1 [OS=Homo sapiens]	HK1	14	226	14	16	61.779	cellular homeostasis;metabolic process;regulation of biological process
Q8TCT9-1	Minor histocompatibility antigen H13 [OS=Homo sapiens]	HM13	3	26	3	12	16.831	cell organization and biogenesis;metabolic process
Q13151	Heterogeneous nuclear ribonucleoprotein A0 [OS=Homo sapiens]	HNRNP A0	4	64	3	19	23.039	defense response;metabolic process;regulation of biological process;response to stimulus
P51991-1	Heterogeneous nuclear ribonucleoprotein A3 [OS=Homo sapiens]	HNRNP A3	7	95	5	20	39.782	metabolic process;transport
P52597	Heterogeneous nuclear ribonucleoprotein F [OS=Homo sapiens]	HNRNP F	7	181	5	26	47.484	metabolic process;regulation of biological process;response to stimulus
P31943	Heterogeneous nuclear ribonucleoprotein H [OS=Homo sapiens]	HNRNP H1	12	402	4	34	103.361	metabolic process;regulation of biological process;response to stimulus
P55795	heterogeneous nuclear ribonucleoprotein H2 [OS=Homo sapiens]	HNRNP H2	12	303	5	34	94.078	metabolic process
P31942	Heterogeneous nuclear ribonucleoprotein H3 [OS=Homo sapiens]	HNRNP H3	4	72	3	17	24.351	cell differentiation;metabolic process
P14866	Heterogeneous nuclear ribonucleoprotein L [OS=Homo sapiens]	HNRNP L	7	43	7	19	21.286	metabolic process;regulation of biological process
O43390-1	heterogeneous nuclear ribonucleoprotein r [OS=Homo sapiens]	HNRNP R	5	67	2	9	20.585	metabolic process;regulation of biological process
Q1KMD3	heterogeneous nuclear ribonucleoprotein U-like protein 2 [OS=Homo sapiens]	HNRNP UL2	9	140	9	15	50.875	
Q5SSJ5-1	Heterochromatin protein 1-binding protein 3 [OS=Homo sapiens]	HP1BP3	6	62	6	11	22.697	cell organization and biogenesis;regulation of biological process;response to stimulus
Q99714-1	3-hydroxyacyl-CoA dehydrogenase type-2 [OS=Homo sapiens]	HSD17 B10	4	50	4	24	21.153	cell organization and biogenesis;metabolic process
P51659-1	peroxisomal multifunctional enzyme type 2 [OS=Homo sapiens]	HSD17 B4	5	34	5	10	22.901	cell differentiation;metabolic process

Q92598	Heat shock protein 105 kDa [OS=Homo sapiens]	HSPH1	12	154	10	19	76.728	metabolic process;regulation of biological process;response to stimulus;transport
Q7Z6Z7	E3 ubiquitin-protein ligase HUWE1 [OS=Homo sapiens]	HUWE1	9	42	9	3	35.443	cell differentiation;cell organization and biogenesis;metabolic process;response to stimulus;transport
P41252	isoleucine--tRNA ligase, cytoplasmic [OS=Homo sapiens]	IARS	12	110	12	11	51.576	cell differentiation;metabolic process;regulation of biological process
Q9NSE4	Isoleucine--tRNA ligase, mitochondrial [OS=Homo sapiens]	IARS2	5	49	5	6	23.511	metabolic process;regulation of biological process
Q16666-1	gamma-interferon-inducible protein 16 [OS=Homo sapiens]	IFI16	5	56	5	8	23.879	cell communication;cell death;cell differentiation;cell proliferation;defense response;metabolic process;regulation of biological process;response to stimulus
Q9NZI8	Insulin-like growth factor 2 mRNA-binding protein 1 [OS=Homo sapiens]	IGF2BP 1	6	121	5	14	34.943	cell proliferation;regulation of biological process;transport
Q9Y6M1	Insulin-like growth factor 2 mRNA-binding protein 2 [OS=Homo sapiens]	IGF2BP 2	8	102	6	18	40.57	regulation of biological process;transport
O00425	Insulin-like growth factor 2 mRNA-binding protein 3 [OS=Homo sapiens]	IGF2BP 3	8	184	6	20	55.213	metabolic process;regulation of biological process;transport
Q70UQ0	Inhibitor of nuclear factor kappa-B kinase-interacting protein [OS=Homo sapiens]	IKBIP	7	46	5	20	27.242	response to stimulus
Q70UQ0-4	Isoform 4 of Inhibitor of nuclear factor kappa-B kinase-interacting protein [OS=Homo sapiens]	IKBIP	10	53	8	31	47.702	response to stimulus
Q8TEX9-1	Importin-4 [OS=Homo sapiens]	IPO4	5	28	5	5	21.79	cell organization and biogenesis;transport
O00410	Importin-5 [OS=Homo sapiens]	IPO5	7	71	7	7	39.1	regulation of biological process;response to stimulus;transport

O95373	Importin-7 [OS=Homo sapiens]	IPO7	7	51	6	9	30.846	defense response;regulation of biological process;response to stimulus;transport
Q96P70	Importin-9 [OS=Homo sapiens]	IPO9	2	23	2	4	19.99	transport
P46940	Ras GTPase-activating-like protein IQGAP1 [OS=Homo sapiens]	IQGAP1	42	822	42	33	290.073	cell growth;regulation of biological process;response to stimulus;transport
P16144-1	Integrin beta-4 [OS=Homo sapiens]	ITGB4	6	64	6	4	27.067	cell communication;cell differentiation;cell organization and biogenesis;cellular component movement;metabolic process;regulation of biological process;response to stimulus
Q15046	Lysine--tRNA ligase [OS=Homo sapiens]	KARS	10	46	10	19	34.724	metabolic process;regulation of biological process;response to stimulus
O00139	Kinesin-like protein KIF2A [OS=Homo sapiens]	KIF2A	3	20	3	4	9.089	cell differentiation;cell division;cell organization and biogenesis;cellular component movement;development;transport
Q14974	Importin subunit beta-1 [OS=Homo sapiens]	KPNB1	6	66	6	9	27.52	cell organization and biogenesis;cellular component movement;metabolic process;regulation of biological process;response to stimulus;transport
Q9P2J5	Leucine--tRNA ligase, cytoplasmic [OS=Homo sapiens]	LARS	9	114	9	10	38.862	cell communication;cell organization and biogenesis;metabolic process;regulation of biological process;response to stimulus;transport
P49257	Protein ERGIC-53 [OS=Homo sapiens]	LMAN1	3	36	3	6	10.963	cell organization and biogenesis;coagulation;metabolic process;regulation of biological process;transport
Q7L5N7	Lysophosphatidylcholine acyltransferase 2 [OS=Homo sapiens]	LPCAT2	8	122	8	25	48.991	cell organization and biogenesis;metabolic process
P42704	Leucine-rich PPR motif-containing protein, mitochondrial [OS=Homo sapiens]	LRPPR C	12	107	12	12	54.149	cellular component movement;metabolic process;regulation of biological process;transport

P09960-1	leukotriene A-4 hydrolase [OS=Homo sapiens]	LTA4H	6	68	6	12	44.086	metabolic process;transport
Q9UPN3	Microtubule-actin cross-linking factor 1, isoforms 1/2/3/5 [OS=Homo sapiens]	MACF1	15	128	4	2	52.118	cell organization and biogenesis;regulation of biological process;response to stimulus;transport
P46821	microtubule-associated protein 1B [OS=Homo sapiens]	MAP1B	2	32	2	2	11.527	cell growth;cell organization and biogenesis;cellular component movement;regulation of biological process;transport
P27816-1	Microtubule-associated protein 4 [OS=Homo sapiens]	MAP4	3	23	3	4	9.727	cell division;cell organization and biogenesis;cellular component movement;regulation of biological process
P56192	Methionine--tRNA ligase, cytoplasmic [OS=Homo sapiens]	MARS	4	45	4	6	14.988	cell communication;metabolic process;regulation of biological process;response to stimulus
P43243	Matrin-3 [OS=Homo sapiens]	MATR3	10	129	10	18	52.57	defense response;regulation of biological process;response to stimulus
P49736	DNA replication licensing factor mcm2 [OS=Homo sapiens]	MCM2	5	53	5	7	22.048	cell death;cell organization and biogenesis;metabolic process;response to stimulus
P25205	DNA replication licensing factor mcm3 [OS=Homo sapiens]	MCM3	9	84	9	14	41.663	metabolic process
P33992	DNA replication licensing factor mcm5 [OS=Homo sapiens]	MCM5	6	66	6	11	22.777	cell division;metabolic process
Q14566	DNA replication licensing factor MCM6 [OS=Homo sapiens]	MCM6	5	52	5	10	25.499	metabolic process
P33993-1	DNA replication licensing factor MCM7 [OS=Homo sapiens]	MCM7	7	47	7	13	22.492	cell proliferation;metabolic process;regulation of biological process;response to stimulus
O60502	protein O-GlcNAcase [OS=Homo sapiens]	MGEA5	3	10	3	4	11.843	metabolic process
P11586	C-1-tetrahydrofolate synthase, cytoplasmic [OS=Homo sapiens]	MTHFD 1	13	185	13	17	62.894	metabolic process
Q6UB35-1	Monofunctional C1-tetrahydrofolate synthase, mitochondrial [OS=Homo sapiens]	MTHFD 1L	3	33	3	4	13.247	metabolic process

Q96S97	myeloid-associated differentiation marker [OS=Homo sapiens]	MYADM	2	48	2	10	12.224	cell organization and biogenesis;regulation of biological process
P35579-1	Myosin-9 [OS=Homo sapiens]	MYH9	19	121	15	13	84.955	cell differentiation;cell organization and biogenesis;cellular component movement;metabolic process;regulation of biological process;response to stimulus;transport
Q9BXJ9	N-alpha-acetyltransferase 15, NatA auxiliary subunit [OS=Homo sapiens]	NAA15	6	55	6	7	24.723	cell differentiation;development;metabolic process;regulation of biological process
P43490	nicotinamide phosphoribosyltransferase [OS=Homo sapiens]	NAMPT	8	81	8	20	32.365	cell communication;metabolic process;regulation of biological process;response to stimulus
P55209	Nucleosome assembly protein 1-like 1 [OS=Homo sapiens]	NAP1L1	3	65	2	12	21.537	cell organization and biogenesis;metabolic process;regulation of biological process
O43776	Asparagine--tRNA ligase, cytoplasmic [OS=Homo sapiens]	NARS	5	37	5	9	22.133	metabolic process
Q6PIU2	Neutral cholesterol ester hydrolase 1 [OS=Homo sapiens]	NCEH1	6	123	6	23	41.476	metabolic process
Q9Y2A7-1	Nck-associated protein 1 [OS=Homo sapiens]	NCKAP 1	7	43	7	9	22.595	cell death;cell organization and biogenesis;regulation of biological process;response to stimulus
O75251	NADH dehydrogenase [ubiquinone] iron-sulfur protein 7, mitochondrial [OS=Homo sapiens]	NDUFS 7	2	27	2	11	13.756	cell organization and biogenesis;metabolic process;transport
Q8TD19	Serine/threonine-protein kinase Nek9 [OS=Homo sapiens]	NEK9	2	20	2	2	10.033	cell division;cell organization and biogenesis;metabolic process
P55769	NHP2-like protein 1 [OS=Homo sapiens]	NHP2L1 ; SNU13	2	17	2	19	8.397	cell organization and biogenesis;metabolic process
Q15233	Non-POU domain-containing octamer-binding protein [OS=Homo sapiens]	NONO	8	139	8	30	41.014	defense response;metabolic process;regulation of biological process;response to stimulus
O00567	Nucleolar protein 56 [OS=Homo sapiens]	NOP56	6	49	6	16	24.337	metabolic process

P29474	Nitric oxide synthase, endothelial [OS=Homo sapiens]	NOS3	21	199	21	23	111.995	cell organization and biogenesis;cellular component movement;cellular homeostasis;metabolic process;regulation of biological process;response to stimulus
P55786	puromycin-sensitive aminopeptidase [OS=Homo sapiens]	NPEPPS	17	241	17	21	82.959	metabolic process;response to stimulus
P06748	Nucleophosmin [OS=Homo sapiens]	NPM1	3	42	3	14	13.756	cell growth;cell organization and biogenesis;cellular homeostasis;metabolic process;regulation of biological process;response to stimulus;transport
P15559-1	NAD(P)H dehydrogenase [quinone] 1 [OS=Homo sapiens]	NQO1	3	67	3	15	19.779	cell communication;metabolic process;regulation of biological process;response to stimulus
P46459	Vesicle-fusing ATPase [OS=Homo sapiens]	NSF	8	66	8	12	36.537	cell organization and biogenesis;metabolic process;regulation of biological process;response to stimulus;transport
Q9BSD7	Cancer-related nucleoside-triphosphatase [OS=Homo sapiens]	NTPCR	2	25	2	14	12.919	metabolic process;response to stimulus
Q8N1F7-1	Nuclear pore complex protein Nup93 [OS=Homo sapiens]	NUP93	3	21	3	5	11.066	cell organization and biogenesis;cellular component movement;metabolic process;regulation of biological process;response to stimulus;transport
P04181-1	Ornithine aminotransferase, mitochondrial [OS=Homo sapiens]	OAT	5	48	5	14	21.912	cell organization and biogenesis;metabolic process
Q56VL3	OCIA domain-containing protein 2 [OS=Homo sapiens]	OCIAD2	3	23	3	20	11.771	
Q96FW1	Ubiquitin thioesterase otub1 [OS=Homo sapiens]	OTUB1	3	23	3	13	11.909	metabolic process;regulation of biological process;response to stimulus
P07237	Protein disulfide-isomerase [OS=Homo sapiens]	P4HB	5	82	5	11	26.87	cell organization and biogenesis;cellular

								homeostasis;metabolic process;regulation of biological process;response to stimulus
Q9UQ80	proliferation-associated protein 2G4 [OS=Homo sapiens]	PA2G4	6	45	6	18	18.858	cell proliferation;metabolic process;regulation of biological process;transport
Q13177	Serine/threonine-protein kinase PAK 2 [OS=Homo sapiens]	PAK2	5	26	5	14	16.71	cell death;cell organization and biogenesis;cellular component movement;metabolic process;regulation of biological process;response to stimulus
O95340	bifunctional 3'-phosphoadenosine 5'-phosphosulfate synthase 2 [OS=Homo sapiens]	PAPSS 2	7	85	7	14	32.162	coagulation;metabolic process
Q99497	protein/nucleic acid deglycase DJ-1 [OS=Homo sapiens]	PARK7	6	49	6	46	21.783	cell communication;cell organization and biogenesis;defense response;metabolic process;regulation of biological process;response to stimulus;transport
P09874	Poly [ADP-ribose] polymerase 1 [OS=Homo sapiens]	PARP1	6	51	6	7	24.494	cell death;cell differentiation;cell organization and biogenesis;metabolic process;regulation of biological process;response to stimulus
P08559	Pyruvate dehydrogenase E1 component subunit alpha, somatic form, mitochondrial [OS=Homo sapiens]	PDHA1	4	39	4	11	18.638	metabolic process;regulation of biological process
P11177	Pyruvate dehydrogenase E1 component subunit beta, mitochondrial [OS=Homo sapiens]	PDHB	6	69	6	22	30.202	metabolic process;regulation of biological process
Q14554	Protein disulfide-isomerase A5 [OS=Homo sapiens]	PDIA5	4	23	4	10	13.463	cellular homeostasis;metabolic process;regulation of biological process;response to stimulus
P16284	Platelet endothelial cell adhesion molecule [OS=Homo sapiens]	PECAM 1	7	77	7	14	34.047	cell organization and biogenesis;cellular component movement;regulation of biological process;response to stimulus;transport

P12955	xaa-Pro dipeptidase [OS=Homo sapiens]	PEPD	2	35	2	4	8.018	metabolic process
P17858-1	ATP-dependent 6-phosphofructokinase, liver type [OS=Homo sapiens]	PFKL	4	100	2	7	31.944	cell organization and biogenesis;metabolic process;regulation of biological process;response to stimulus;transport
Q01813	ATP-dependent 6-phosphofructokinase, platelet type [OS=Homo sapiens]	PFKP	19	321	17	29	119.994	metabolic process
P35080-2	Isoform IIb of Profilin-2 [OS=Homo sapiens]	PFN2	3	50	3	26	19.509	cell organization and biogenesis;regulation of biological process
P18669	Phosphoglycerate mutase 1 [OS=Homo sapiens]	PGAM1; LOC643576	5	58	5	26	26.652	metabolic process;regulation of biological process;transport
O95336	6-phosphogluconolactonase [OS=Homo sapiens]	PGLS	6	68	6	34	25.338	metabolic process
Q96G03	Phosphoglucomutase-2 [OS=Homo sapiens]	PGM2	3	36	2	7	14.683	metabolic process;transport
Q6PCE3	glucose 1,6-bisphosphate synthase [OS=Homo sapiens]	PGM2L1	6	51	6	10	18.964	metabolic process
Q99623	Prohibitin-2 [OS=Homo sapiens]	PHB2	11	164	11	42	74.149	cell organization and biogenesis;metabolic process;regulation of biological process;response to stimulus;transport
O60664-1	Perilipin-3 [OS=Homo sapiens]	PLIN3	5	31	5	18	26.583	transport
Q02809	Procollagen-lysine,2-oxoglutarate 5-dioxygenase 1 [OS=Homo sapiens]	PLOD1	2	10	2	4	9.28	metabolic process;response to stimulus
O60568	Procollagen-lysine,2-oxoglutarate 5-dioxygenase 3 [OS=Homo sapiens]	PLOD3	2	32	2	4	11.701	cell organization and biogenesis;metabolic process;response to stimulus
P29590	Protein PML [OS=Homo sapiens]	PML	6	27	6	10	26.218	cell death;cell differentiation;cell organization and biogenesis;cellular component movement;cellular homeostasis;defense response;metabolic process;regulation of biological process;response to stimulus;transport

P00491	purine nucleoside phosphorylase [OS=Homo sapiens]	PNP	5	103	5	21	29.711	metabolic process;regulation of biological process;response to stimulus;transport
Q8IY17-1	Neuropathy target esterase [OS=Homo sapiens]	PNPLA6	4	47	4	5	18.904	metabolic process
Q9H488-1	GDP-fucose protein O-fucosyltransferase 1 [OS=Homo sapiens]	POFUT 1	2	40	2	7	9.933	metabolic process;regulation of biological process;response to stimulus
P49593	Protein phosphatase 1F [OS=Homo sapiens]	PPM1F	4	98	4	14	24.272	cell death;cell organization and biogenesis;metabolic process;regulation of biological process;response to stimulus
P36873-1	serine/threonine-protein phosphatase PP1-gamma catalytic subunit [OS=Homo sapiens]	PPP1C C	4	35	2	18	20.548	cell differentiation;cell division;cell organization and biogenesis;metabolic process;regulation of biological process;response to stimulus
P62714	serine/threonine-protein phosphatase 2A catalytic subunit beta isoform [OS=Homo sapiens]	PPP2C B	4	36	4	18	19.018	cell organization and biogenesis;metabolic process;regulation of biological process;response to stimulus
P30153	serine/threonine-protein phosphatase 2A 65 kDa regulatory subunit A alpha isoform [OS=Homo sapiens]	PPP2R1 A	9	87	9	20	50.644	cell death;cell organization and biogenesis;metabolic process;regulation of biological process;response to stimulus
O60831	PRA1 family protein 2 [OS=Homo sapiens]	PRAF2	2	21	2	12	10.283	transport
P30048	Thioredoxin-dependent peroxide reductase, mitochondrial [OS=Homo sapiens]	PRDX3	2	29	2	10	10.072	cell death;cell differentiation;cell organization and biogenesis;cellular homeostasis;metabolic process;regulation of biological process;response to stimulus
P30044-1	Peroxiredoxin-5, mitochondrial [OS=Homo sapiens]	PRDX5	6	76	6	36	23.842	cell death;cellular homeostasis;defense response;metabolic process;regulation of biological process;response to stimulus
Q9UMS4	Pre-mRNA-processing factor 19 [OS=Homo sapiens]	PRPF19	2	34	2	5	11.004	cell organization and biogenesis;cell proliferation;metabolic process;regulation of biological process;response to stimulus

O43172-1	U4/U6 small nuclear ribonucleoprotein Prp4 [OS=Homo sapiens]	PRPF4	2	12	2	6	9.526	metabolic process
O94906-1	Pre-mRNA-processing factor 6 [OS=Homo sapiens]	PRPF6	5	46	5	6	18.624	cell organization and biogenesis;metabolic process;regulation of biological process
Q6P2Q9	Pre-mRNA-processing-splicing factor 8 [OS=Homo sapiens]	PRPF8	9	88	9	4	39.331	cell organization and biogenesis;metabolic process;response to stimulus
P25789	Proteasome subunit alpha type-4 [OS=Homo sapiens]	PSMA4	4	44	4	18	16.736	metabolic process;regulation of biological process;response to stimulus;transport
O14818-1	Proteasome subunit alpha type-7 [OS=Homo sapiens]	PSMA7	3	58	3	14	17.679	metabolic process;regulation of biological process;response to stimulus;transport
P28070	Proteasome subunit beta type-4 [OS=Homo sapiens]	PSMB4	3	27	3	15	10.841	metabolic process;regulation of biological process;response to stimulus;transport
P62191	26S proteasome regulatory subunit 4 [OS=Homo sapiens]	PSMC1	6	80	5	19	27.305	metabolic process;regulation of biological process;response to stimulus;transport
P17980	26S proteasome regulatory subunit 6A [OS=Homo sapiens]	PSMC3	10	102	10	28	41.944	metabolic process;regulation of biological process;response to stimulus;transport
P43686	26S proteasome regulatory subunit 6B [OS=Homo sapiens]	PSMC4	6	48	6	17	25.599	metabolic process;regulation of biological process;response to stimulus;transport
P62333	26S proteasome regulatory subunit 10B [OS=Homo sapiens]	PSMC6	3	38	3	10	14.904	cell organization and biogenesis;metabolic process;regulation of biological process;response to stimulus;transport
O00231	26S proteasome non-ATPase regulatory subunit 11 [OS=Homo sapiens]	PSMD11	5	33	5	15	19.531	cell differentiation;cell organization and biogenesis;metabolic process;regulation of biological process;response to stimulus;transport
Q9UNM6	26S proteasome non-ATPase regulatory subunit 13 [OS=Homo sapiens]	PSMD13	4	34	4	9	11.47	cell organization and biogenesis;metabolic process;regulation of biological

								process;response to stimulus;transport
O00487	26S proteasome non-ATPase regulatory subunit 14 [OS=Homo sapiens]	PSMD14	4	24	4	20	11.895	metabolic process;regulation of biological process;response to stimulus;transport
Q13200	26S proteasome non-ATPase regulatory subunit 2 [OS=Homo sapiens]	PSMD2	8	67	8	10	39.613	metabolic process;regulation of biological process;response to stimulus;transport
O43242	26S proteasome non-ATPase regulatory subunit 3 [OS=Homo sapiens]	PSMD3	7	70	7	16	27.884	metabolic process;regulation of biological process;response to stimulus;transport
P55036-1	26S proteasome non-ATPase regulatory subunit 4 [OS=Homo sapiens]	PSMD4	3	22	3	13	8.88	cell organization and biogenesis;metabolic process;regulation of biological process;response to stimulus;transport
Q16401	26S proteasome non-ATPase regulatory subunit 5 [OS=Homo sapiens]	PSMD5	2	15	2	5	6.049	cell organization and biogenesis;metabolic process;regulation of biological process;response to stimulus;transport
Q15008	26S proteasome non-ATPase regulatory subunit 6 [OS=Homo sapiens]	PSMD6	3	18	3	9	10.342	metabolic process;regulation of biological process;response to stimulus;transport
Q92530	proteasome inhibitor PI31 subunit [OS=Homo sapiens]	PSMF1	3	12	3	15	9.965	metabolic process;regulation of biological process;response to stimulus;transport
Q6NZI2-1	caveolae-associated protein 1 [OS=Homo sapiens]	PTRF; CAVIN1	4	28	4	16	16.132	metabolic process;regulation of biological process
Q9Y3E5	Peptidyl-tRNA hydrolase 2, mitochondrial [OS=Homo sapiens]	PTRH2	2	27	2	19	11.23	cell death;regulation of biological process
Q9UHX1-1	poly(U)-binding-splicing factor PUF60 [OS=Homo sapiens]	PUF60	2	17	2	8	8.244	cell death;cellular homeostasis;metabolic process;regulation of biological process
P47897	glutamine--tRNA ligase [OS=Homo sapiens]	QARS	7	60	7	12	25.47	metabolic process;regulation of biological process
P61026	ras-related protein rab-10 [OS=Homo sapiens]	RAB10	3	72	1	17	19.93	cell differentiation;cell organization and biogenesis;regulation of biological process;response to stimulus;transport

P62491-1	Ras-related protein Rab-11A [OS=Homo sapiens]	RAB11A	2	44	2	10	10.353	cell organization and biogenesis;metabolic process;regulation of biological process;response to stimulus;transport
P61019-1	Ras-related protein Rab-2A [OS=Homo sapiens]	RAB2A	4	55	4	25	21.786	cell organization and biogenesis;metabolic process;regulation of biological process;response to stimulus;transport
P51148	Ras-related protein Rab-5C [OS=Homo sapiens]	RAB5C	5	80	4	35	30.094	regulation of biological process;response to stimulus;transport
P51149	ras-related protein Rab-7a [OS=Homo sapiens]	RAB7A	4	81	4	22	28.085	cell organization and biogenesis;cellular homeostasis;metabolic process;regulation of biological process;response to stimulus;transport
Q92878	DNA repair protein Rad50 [OS=Homo sapiens]	RAD50	5	40	5	4	14.101	cell organization and biogenesis;metabolic process;regulation of biological process;response to stimulus
P11233	Ras-related protein Ral-A [OS=Homo sapiens]	RALA	3	33	3	11	13.875	cell division;cell organization and biogenesis;regulation of biological process;response to stimulus;transport
Q9UKM9-1	RNA-binding protein Raly [OS=Homo sapiens]	RALY	3	46	3	9	12.838	metabolic process;regulation of biological process
P46060	Ran GTPase-activating protein 1 [OS=Homo sapiens]	RANGAP1	2	29	2	4	10.147	cell organization and biogenesis;metabolic process;regulation of biological process;response to stimulus
P61224-1	Ras-related protein Rap-1b [OS=Homo sapiens]	RAP1B	4	68	3	19	21.985	cell organization and biogenesis;cell proliferation;regulation of biological process;response to stimulus;transport
P61225	ras-related protein Rap-2b [OS=Homo sapiens]	RAP2B	2	41	2	13	10.78	cell organization and biogenesis;regulation of biological process;response to stimulus;transport

Q96PK6-1	RNA-binding protein 14 [OS=Homo sapiens]	RBM14; RBM14-RBM4	2	21	2	5	6.87	cell organization and biogenesis;defense response;metabolic process;regulation of biological process;response to stimulus
P84098	60S ribosomal protein L19 [OS=Homo sapiens]	RPL19	2	28	2	13	9.714	cell organization and biogenesis;metabolic process;transport
P46778	60S ribosomal protein L21 [OS=Homo sapiens]	RPL21	3	32	3	28	15.186	cell organization and biogenesis;metabolic process;transport
P46779	60S ribosomal protein L28 [OS=Homo sapiens]	RPL28	3	37	3	27	11.605	cell organization and biogenesis;metabolic process;regulation of biological process;transport
P39023	60S ribosomal protein L3 [OS=Homo sapiens]	RPL3	6	105	6	18	36.542	cell organization and biogenesis;metabolic process;response to stimulus;transport
P62910	60S ribosomal protein L32 [OS=Homo sapiens]	RPL32	5	39	5	42	19.528	cell organization and biogenesis;metabolic process;response to stimulus;transport
P49207	60S ribosomal protein L34 [OS=Homo sapiens]	RPL34	2	59	2	13	10.187	cell organization and biogenesis;metabolic process;transport
P18077	60S ribosomal protein L35a [OS=Homo sapiens]	RPL35A	3	17	3	14	13.336	cell organization and biogenesis;metabolic process;transport
P63173	60s ribosomal protein l38 [OS=Homo sapiens]	RPL38	4	29	4	50	14.067	cell organization and biogenesis;metabolic process;regulation of biological process;transport
P46777	60S ribosomal protein L5 [OS=Homo sapiens]	RPL5	7	150	7	29	44.343	cell organization and biogenesis;metabolic process;regulation of biological process;transport
P62917	60S ribosomal protein L8 [OS=Homo sapiens]	RPL8	5	71	5	22	25.443	cell organization and biogenesis;metabolic process;response to stimulus;transport

P32969	60S ribosomal protein L9 [OS=Homo sapiens]	RPL9	5	124	5	40	24.721	cell organization and biogenesis;metabolic process;transport
P04844-1	Dolichyl-diphosphooligosaccharide--protein glycosyltransferase subunit 2 [OS=Homo sapiens]	RPN2	5	102	5	11	26.222	metabolic process;response to stimulus
Q9NQG5	regulation of nuclear pre-mRNA domain-containing protein 1B [OS=Homo sapiens]	RPRD1B	3	36	3	14	13.411	metabolic process;regulation of biological process
P62277	40S ribosomal protein S13 [OS=Homo sapiens]	RPS13	5	67	5	30	18.254	cell organization and biogenesis;metabolic process;regulation of biological process;transport
P08708	40S ribosomal protein S17 [OS=Homo sapiens]	RPS17; RPS17L	3	27	3	40	10.947	cell organization and biogenesis;metabolic process;transport
P62269	40S ribosomal protein S18 [OS=Homo sapiens]	RPS18	2	36	2	11	10.496	cell organization and biogenesis;metabolic process;transport
P60866	40S ribosomal protein S20 [OS=Homo sapiens]	RPS20	4	66	4	29	23.442	cell organization and biogenesis;metabolic process;transport
P62847-1	40S ribosomal protein S24 [OS=Homo sapiens]	RPS24	4	63	4	35	22.684	cell organization and biogenesis;metabolic process;transport
P62854	40S ribosomal protein S26 [OS=Homo sapiens]	RPS26; LOC101929876; RPS26P25	3	66	3	34	16.111	cell organization and biogenesis;metabolic process;regulation of biological process;transport
P62979	Ubiquitin-40S ribosomal protein S27a [OS=Homo sapiens]	RPS27A	9	550	2	60	71.201	cell communication;cell organization and biogenesis;cellular homeostasis;defense response;metabolic process;regulation of biological process;response to stimulus;transport
P46782	40S ribosomal protein S5 [OS=Homo sapiens]	RPS5	4	63	4	27	32.472	cell organization and biogenesis;metabolic process;regulation of biological process;transport

O76021	Ribosomal L1 domain-containing protein 1 [OS=Homo sapiens]	RSL1D1	4	28	4	11	13.41	cell differentiation;metabolic process;regulation of biological process
Q9Y265	RuvB-like 1 [OS=Homo sapiens]	RUVBL 1	4	35	4	13	24.299	cell division;cell organization and biogenesis;metabolic process;regulation of biological process;response to stimulus
Q9Y230	RuvB-like 2 [OS=Homo sapiens]	RUVBL 2	9	156	9	24	48.597	cell organization and biogenesis;metabolic process;regulation of biological process;response to stimulus
P60903	Protein S100-A10 [OS=Homo sapiens]	S100A1 0	2	35	2	28	10.82	cell organization and biogenesis;regulation of biological process;response to stimulus
O75396	Vesicle-trafficking protein SEC22b [OS=Homo sapiens]	SEC22B	3	38	3	21	17.174	cell organization and biogenesis;regulation of biological process;transport
Q15436	protein transport protein Sec23A [OS=Homo sapiens]	SEC23A	9	109	9	16	46.715	cell organization and biogenesis;transport
P53992	Protein transport protein sec24c [OS=Homo sapiens]	SEC24 C	5	29	5	6	20.303	cell organization and biogenesis;regulation of biological process;transport
P61619	Protein transport protein Sec61 subunit alpha isoform 1 [OS=Homo sapiens]	SEC61A 1	3	39	3	5	14.945	cell growth;cell organization and biogenesis;response to stimulus;transport
Q15019	septin-2 [OS=Homo sapiens]	SEPT2	6	128	5	22	37.314	cell differentiation;cell division;cell organization and biogenesis;regulation of biological process;response to stimulus
Q9UHD8-1	Septin-9 [OS=Homo sapiens]	SEPT9	5	55	4	9	24.754	cell division;cell organization and biogenesis;regulation of biological process
O75533-1	splicing factor 3B subunit 1 [OS=Homo sapiens]	SF3B1	5	58	5	4	20.06	cell organization and biogenesis;metabolic process;regulation of biological process;response to stimulus
Q15393-1	Splicing factor 3B subunit 3 [OS=Homo sapiens]	SF3B3	2	27	2	2	10.51	cell organization and biogenesis;metabolic process;regulation of biological process

P23246-1	splicing factor, proline- and glutamine-rich [OS=Homo sapiens]	SFPQ	5	33	5	9	22.56	cell organization and biogenesis;defense response;metabolic process;regulation of biological process;response to stimulus
Q9H9B4	Sideroflexin-1 [OS=Homo sapiens]	SFXN1	3	63	3	11	21.712	cell differentiation;transport
Q9BWM7	Sideroflexin-3 [OS=Homo sapiens]	SFXN3	4	71	4	18	20.592	transport
Q02978	Mitochondrial 2-oxoglutarate/malate carrier protein [OS=Homo sapiens]	SLC25A11	3	44	3	15	16.51	metabolic process;transport
Q6NUK1	Calcium-binding mitochondrial carrier protein SCaMC-1 [OS=Homo sapiens]	SLC25A24	4	32	4	9	16.206	regulation of biological process;response to stimulus;transport
P12236	ADP/ATP translocase 3 [OS=Homo sapiens]	SLC25A6	9	321	2	35	53.298	cell death;cell organization and biogenesis;regulation of biological process;response to stimulus;transport
O95347-1	structural maintenance of chromosomes protein 2 [OS=Homo sapiens]	SMC2	2	16	2	2	6.309	cell division;cell organization and biogenesis;metabolic process;regulation of biological process
O75643-1	U5 small nuclear ribonucleoprotein 200 kDa helicase [OS=Homo sapiens]	SNRNP200	8	94	8	5	40.716	cell differentiation;cell organization and biogenesis;metabolic process
Q13596	Sorting nexin-1 [OS=Homo sapiens]	SNX1	2	26	1	3	10.142	cell organization and biogenesis;metabolic process;regulation of biological process;transport
Q9UNH7-1	Sorting nexin-6 [OS=Homo sapiens]	SNX6	3	17	3	9	11.681	cell organization and biogenesis;regulation of biological process;transport
Q13813	Spectrin alpha chain, non-erythrocytic 1 [OS=Homo sapiens]	SPTAN1	7	44	7	4	32.09	cell organization and biogenesis;cellular component movement;metabolic process;regulation of biological process;response to stimulus;transport
Q01082-1	Spectrin beta chain, non-erythrocytic 1 [OS=Homo sapiens]	SPTBN1	22	218	22	13	101.245	cell organization and biogenesis;cellular component movement;metabolic

								process;regulation of biological process;response to stimulus;transport
Q9Y6N5	Sulfide:quinone oxidoreductase, mitochondrial [OS=Homo sapiens]	SQRDL; SQOR	6	61	6	14	29.073	metabolic process
P19623	spermidine synthase [OS=Homo sapiens]	SRM	5	60	5	20	19.628	metabolic process
P37108	Signal recognition particle 14 kDa protein [OS=Homo sapiens]	SRP14	4	24	4	40	31.032	cell organization and biogenesis;response to stimulus;transport
Q9UHB9	Signal recognition particle subunit SRP68 [OS=Homo sapiens]	SRP68	3	63	3	6	15.869	cell organization and biogenesis;response to stimulus;transport
Q08945	FACT complex subunit SSRP1 [OS=Homo sapiens]	SSRP1	4	41	4	7	22.872	metabolic process;regulation of biological process;response to stimulus
P42224-1	Signal transducer and activator of transcription 1-alpha/beta [OS=Homo sapiens]	STAT1	5	63	5	10	27.011	cell death;cell differentiation;cell proliferation;cellular component movement;defense response;metabolic process;regulation of biological process;response to stimulus
P31948	stress-induced-phosphoprotein 1 [OS=Homo sapiens]	STIP1	6	61	6	14	27.677	response to stimulus
Q8TCJ2	Dolichyl-diphosphooligosaccharide--protein glycosyltransferase subunit STT3B [OS=Homo sapiens]	STT3B	4	36	4	5	15.307	metabolic process;response to stimulus
P53597	Succinate--CoA ligase [ADP/GDP-forming] subunit alpha, mitochondrial [OS=Homo sapiens]	SUCLG 1	2	36	2	9	9.71	metabolic process
Q96I99-1	Succinate--CoA ligase [GDP-forming] subunit beta, mitochondrial [OS=Homo sapiens]	SUCLG 2	3	22	3	11	14.595	metabolic process
Q9Y5B9	FACT complex subunit SPT16 [OS=Homo sapiens]	SUPT16 H	7	68	7	8	28.818	cell organization and biogenesis;metabolic process;regulation of biological process;response to stimulus

Q9UH65	Switch-associated protein 70 [OS=Homo sapiens]	SWAP70	2	31	2	3	8.855	cell organization and biogenesis;cellular homeostasis;metabolic process;regulation of biological process
O60506	Heterogeneous nuclear ribonucleoprotein Q [OS=Homo sapiens]	SYNCRIP	8	101	5	15	31.724	cell differentiation;metabolic process;regulation of biological process;response to stimulus
P26639-1	Threonine--tRNA ligase, cytoplasmic [OS=Homo sapiens]	TARS	8	71	8	14	35.619	metabolic process
P07996	thrombospondin-1 [OS=Homo sapiens]	THBS1	4	22	4	5	12.22	cell organization and biogenesis;cellular component movement;defense response;metabolic process;regulation of biological process;response to stimulus;transport
P29401	Transketolase [OS=Homo sapiens]	TKT	3	49	3	8	20.505	metabolic process;regulation of biological process
Q92544	Transmembrane 9 superfamily member 4 [OS=Homo sapiens]	TM9SF4	2	16	2	4	7.315	cell organization and biogenesis;cellular homeostasis;regulation of biological process;response to stimulus;transport
Q13445	Transmembrane emp24 domain-containing protein 1 [OS=Homo sapiens]	TMED1	2	14	2	12	13.983	cell communication;regulation of biological process;response to stimulus;transport
Q86WV6	Stimulator of interferon genes protein [OS=Homo sapiens]	TMEM173	5	111	5	20	48.171	cell death;defense response;regulation of biological process;response to stimulus;transport
Q92973-1	transportin-1 [OS=Homo sapiens]	TNPO1	9	71	9	12	38.835	cellular component movement;regulation of biological process;transport
P11387	DNA topoisomerase 1 [OS=Homo sapiens]	TOP1	6	92	6	8	31.84	cell death;cell division;cell organization and biogenesis;metabolic process;regulation of biological process;response to stimulus
Q02880	DNA topoisomerase 2-beta [OS=Homo sapiens]	TOP2B	8	95	8	6	40.381	cell organization and biogenesis;cellular component

								movement;metabolic process;regulation of biological process
Q14258	E3 ubiquitin/ISG15 ligase TRIM25 [OS=Homo sapiens]	TRIM25	5	48	5	9	25.203	defense response;metabolic process;regulation of biological process;response to stimulus
Q13263	Transcription intermediary factor 1-beta [OS=Homo sapiens]	TRIM28	8	83	8	15	34.512	cell organization and biogenesis;defense response;metabolic process;regulation of biological process;response to stimulus
Q9BQE3	Tubulin alpha-1C chain [OS=Homo sapiens]	TUBA1C	20	2147	3	62	253.702	cell division;cell organization and biogenesis;transport
Q13509	tubulin beta-3 chain [OS=Homo sapiens]	TUBB3	19	2010	2	45	264.623	cell organization and biogenesis;cellular component movement
P04350	Tubulin beta-4A chain [OS=Homo sapiens]	TUBB4A	26	2013	3	69	293.924	cell organization and biogenesis;regulation of biological process
Q12792-2	Twinfilin-1 [OS=Homo sapiens]	TWF1	3	27	3	11	12.988	cell organization and biogenesis;regulation of biological process
Q6IBS0	Twinfilin-2 [OS=Homo sapiens]	TWF2	2	36	2	10	17.497	cell organization and biogenesis;regulation of biological process;response to stimulus
Q16222-1	UDP-N-acetylhexosamine pyrophosphorylase [OS=Homo sapiens]	UAP1	3	60	3	7	16.691	metabolic process
Q9UBT2	SUMO-activating enzyme subunit 2 [OS=Homo sapiens]	UBA2	6	46	6	12	25.263	metabolic process;regulation of biological process
Q13404	Ubiquitin-conjugating enzyme E2 variant 1 [OS=Homo sapiens]	UBE2V1; TMEM189-UBE2V1	4	20	4	27	12.783	cell differentiation;metabolic process;regulation of biological process;response to stimulus
Q9H3U1	Protein unc-45 homolog A [OS=Homo sapiens]	UNC45A	5	28	5	7	23.91	cell differentiation;development;metabolic process
P22695	Cytochrome b-c1 complex subunit 2, mitochondrial [OS=Homo sapiens]	UQCRC2	9	96	9	26	47.904	metabolic process;transport

O60763-1	General vesicular transport factor p115 [OS=Homo sapiens]	USO1	6	69	6	9	25.172	cell organization and biogenesis;metabolic process;transport
Q14694	Ubiquitin carboxyl-terminal hydrolase 10 [OS=Homo sapiens]	USP10	2	20	2	3	8.064	metabolic process;regulation of biological process;response to stimulus
P45974-1	Ubiquitin carboxyl-terminal hydrolase 5 [OS=Homo sapiens]	USP5	8	51	8	14	33.21	metabolic process;regulation of biological process
Q93008-1	Isoform 2 of Probable ubiquitin carboxyl-terminal hydrolase FAF-X [OS=Homo sapiens]	USP9X	4	18	4	2	11.548	cell division;cell growth;cellular component movement;metabolic process;regulation of biological process;response to stimulus
P46939	Utrophin [OS=Homo sapiens]	UTRN	10	74	10	4	35.721	cell organization and biogenesis;regulation of biological process;response to stimulus
P26640	Valine--tRNA ligase [OS=Homo sapiens]	VARS; VARS2	6	24	6	7	20.586	metabolic process;regulation of biological process
P18206	Vinculin [OS=Homo sapiens]	VCL	13	111	13	14	61.836	cell growth;cell organization and biogenesis;cellular component movement;regulation of biological process;transport
Q9Y277	Voltage-dependent anion-selective channel protein 3 [OS=Homo sapiens]	VDAC3	3	24	2	12	15.252	cell communication;defense response;regulation of biological process;response to stimulus;transport
O75436-1	Vacuolar protein sorting-associated protein 26A [OS=Homo sapiens]	VPS26A	2	15	2	9	7.735	regulation of biological process;response to stimulus;transport
Q96QK1	Vacuolar protein sorting-associated protein 35 [OS=Homo sapiens]	VPS35	8	83	8	10	30.047	cell organization and biogenesis;regulation of biological process;response to stimulus;transport
P23381	Tryptophan--tRNA ligase, cytoplasmic [OS=Homo sapiens]	WARS	3	28	3	10	11.966	metabolic process;regulation of biological process
O14980	Exportin-1 [OS=Homo sapiens]	XPO1	10	103	10	11	46.747	cell organization and biogenesis;regulation of biological process;response to stimulus;transport
Q9UIA9	exportin-7 [OS=Homo sapiens]	XPO7	4	40	4	4	14.299	transport
O43592	Exportin-T [OS=Homo sapiens]	XPOT	3	20	3	4	11.284	transport

P12956	X-ray repair cross-complementing protein 6 [OS=Homo sapiens]	XRCC6	15	173	15	31	70.545	cell organization and biogenesis;defense response;metabolic process;regulation of biological process;response to stimulus;transport
P31946	14-3-3 protein beta/alpha [OS=Homo sapiens]	YWHAB	5	163	2	27	21.855	cell organization and biogenesis;metabolic process;regulation of biological process;response to stimulus;transport
Q04917	14-3-3 protein eta [OS=Homo sapiens]	YWHAH	7	190	5	25	41.44	cell organization and biogenesis;metabolic process;regulation of biological process;response to stimulus;transport
P63104-1	14-3-3 protein zeta/delta [OS=Homo sapiens]	YWHAZ	7	207	5	29	57.138	cell organization and biogenesis;metabolic process;regulation of biological process;response to stimulus;transport

Table S3: List of 131 proteins modified by only 2-ClHDyA in HLMVEC

Accession	Description	Gene Symbol	# Peptides	# PSMs	# Unique Peptides	Coverage [%]	Sum PEP Score	Biological Process
P09110	3-ketoacyl-CoA thiolase, peroxisomal [OS=Homo sapiens]	ACAA1	3	22	3	13	22.974	other metabolic processes
O00154	Isoform 4 of Cytosolic acyl coenzyme A thioester hydrolase [OS=Homo sapiens]	ACOT7	6	75	6	23	28.271	
O95573	long-chain-fatty-acid--CoA ligase 3 [OS=Homo sapiens]	ACSL3	6	27	6	9	21.406	cell organization and biogenesis;other metabolic processes;transport;developmental processes;other biological processes
P35611	Alpha-adducin [OS=Homo sapiens]	ADD1	5	52	5	10	18.85	cell organization and biogenesis;other biological processes
P11766	alcohol dehydrogenase class-3 [OS=Homo sapiens]	ADH5	2	19	2	7	5.625	protein metabolism;other metabolic processes;stress response;other biological processes
P30566	adenylosuccinate lyase [OS=Homo sapiens]	ADSL	2	48	2	4	11.027	other metabolic processes;stress response;other biological processes
Q09666	Neuroblast differentiation-associated protein AHNAK [OS=Homo sapiens]	AHNAK	6	94	6	3	27.183	other biological processes
P00568	Adenylate kinase isoenzyme 1 [OS=Homo sapiens]	AK1	2	27	2	12	10.902	other metabolic processes
P54886	delta-1-pyrroline-5-carboxylate synthase [OS=Homo sapiens]	ALDH18A1	6	92	6	8	31.567	other metabolic processes
P09525	annexin A4 [OS=Homo sapiens]	ANXA4	4	28	3	13	15.159	signal transduction;other biological processes
P20073	Annexin A7 [OS=Homo sapiens]	ANXA7	2	22	2	4	6.166	cell organization and biogenesis;other metabolic processes;other biological processes
O43747	AP-1 complex subunit gamma-1 [OS=Homo sapiens]	AP1G1	3	22	3	3	8.547	cell organization and biogenesis;transport;other biological processes
O94973	AP-2 complex subunit alpha-2 [OS=Homo sapiens]	AP2A2	6	64	3	6	26.076	transport
O00203	AP-3 complex subunit beta-1 [OS=Homo sapiens]	AP3B1	3	23	3	3	15.351	cell organization and biogenesis;protein metabolism;RNA metabolism OR transcription;other

								metabolic processes;stress response;transport;developmental processes;signal transduction;other biological processes
Q9HDC9	Adipocyte plasma membrane-associated protein [OS=Homo sapiens]	APMAP	2	42	2	6	10.787	other metabolic processes
P40616	ADP-ribosylation factor-like protein 1 [OS=Homo sapiens]	ARL1	3	37	3	19	13.498	cell organization and biogenesis;other metabolic processes;transport;other biological processes
Q96BM9	ADP-ribosylation factor-like protein 8A [OS=Homo sapiens]	ARL8A	4	64	1	20	18.099	cell cycle OR cell proliferation;transport;other biological processes
Q92747	Actin-related protein 2/3 complex subunit 1A [OS=Homo sapiens]	ARPC1A	2	55	1	4	6.947	cell organization and biogenesis
O15145	Actin-related protein 2/3 complex subunit 3 [OS=Homo sapiens]	ARPC3	3	53	3	16	17.182	cell organization and biogenesis;other biological processes
Q6DD88	Atlastin-3 [OS=Homo sapiens]	ATL3	3	50	3	9	24.845	cell organization and biogenesis;other biological processes
Q9HD20	manganese-transporting ATPase 13A1 [OS=Homo sapiens]	ATP13A1	3	29	3	3	12.426	transport;other biological processes
P23634	Plasma membrane calcium-transporting ATPase 4 [OS=Homo sapiens]	ATP2B4	7	48	7	7	29.056	stress response;transport;developmental processes;other biological processes
P36542	ATP synthase subunit gamma, mitochondrial [OS=Homo sapiens]	ATP5F1C	4	29	4	13	16.924	other metabolic processes;transport
P48047	ATP synthase subunit O, mitochondrial [OS=Homo sapiens]	ATP5PO	4	65	4	21	17.526	other metabolic processes;transport
P51572	B-cell receptor-associated protein 31 [OS=Homo sapiens]	BCAP31	4	35	4	14	15.543	cell organization and biogenesis;stress response;transport;signal transduction;other biological processes
P53004	Biliverdin reductase A [OS=Homo sapiens]	BLVRA	4	42	4	16	19.327	other metabolic processes

P27824	Calnexin [OS=Homo sapiens]	CANX	3	26	3	6	13.919	stress response;transport;signal transduction;other biological processes
P04040	catalase [OS=Homo sapiens]	CAT	4	34	4	9	23.855	protein metabolism;other metabolic processes;stress response;developmental processes;other biological processes
Q6NZI2	caveolae-associated protein 1 [OS=Homo sapiens]	CAVIN1	4	19	4	11	16.343	RNA metabolism OR transcription;other metabolic processes;transport;other biological processes
P16152	Carbonyl reductase [NADPH] 1 [OS=Homo sapiens]	CBR1	6	51	6	30	28.538	other metabolic processes;other biological processes
P21964	Catechol O-methyltransferase [OS=Homo sapiens]	COMT	2	13	2	7	7.85	other metabolic processes;stress response;other biological processes
Q9Y678	Coatomer subunit gamma-1 [OS=Homo sapiens]	COPG1	9	98	9	16	42.333	transport;other biological processes
P19784	casein kinase II subunit alpha' [OS=Homo sapiens]	CSNK2A2	2	34	1	4	7.308	cell-cell signaling;cell cycle OR cell proliferation;protein metabolism;other metabolic processes;developmental processes;signal transduction;other biological processes
P56545	c-terminal-binding protein 2 [OS=Homo sapiens]	CTBP2	2	16	2	4	6.604	cell-cell signaling;cell organization and biogenesis;transport;other biological processes
P35222	Catenin beta-1 [OS=Homo sapiens]	CTNNB1	5	52	3	7	21.891	cell adhesion;cell-cell signaling;cell cycle OR cell proliferation;cell organization and biogenesis;protein metabolism;other metabolic processes;transport;developmental processes;signal transduction;other biological processes
O60716	Catenin delta-1 [OS=Homo sapiens]	CTNND1	6	71	6	7	24.429	cell adhesion;cell-cell signaling;cell organization and biogenesis;developmental processes;signal transduction;other biological processes
Q9UHQ9	NADH-cytochrome b5 reductase 1 [OS=Homo sapiens]	CYB5R1	3	71	3	9	11.109	other metabolic processes;transport

Q9UBM7	7-dehydrocholesterol reductase [OS=Homo sapiens]	DHCR7	3	79	3	6	14.359	other metabolic processes;other biological processes
Q14195	Isoform LCRMP-4 of Dihydropyrimidinase-related protein 3 [OS=Homo sapiens]	DPYSL3	6	152	5	10	27.19	
P63167	Dynein light chain 1, cytoplasmic [OS=Homo sapiens]	DYNLL1	2	18	2	33	6.839	cell organization and biogenesis;other biological processes
Q14152	Eukaryotic translation initiation factor 3 subunit A [OS=Homo sapiens]	EIF3A	8	98	8	5	32.857	cell organization and biogenesis;protein metabolism;other metabolic processes;other biological processes
O15371	Eukaryotic translation initiation factor 3 subunit D [OS=Homo sapiens]	EIF3D	3	51	3	6	15.679	cell organization and biogenesis;protein metabolism;other metabolic processes;other biological processes
Q6P179	Endoplasmic reticulum aminopeptidase 2 [OS=Homo sapiens]	ERAP2	4	53	4	4	16.112	protein metabolism;other metabolic processes;signal transduction;other biological processes
O75477	erlin-1 [OS=Homo sapiens]	ERLIN1	5	96	2	16	25.842	
Q9BS26	Endoplasmic reticulum resident protein 44 [OS=Homo sapiens]	ERP44	4	37	3	9	14.152	protein metabolism;other metabolic processes;stress response;other biological processes
P62495	Eukaryotic peptide chain release factor subunit 1 [OS=Homo sapiens]	ETF1	4	61	4	9	22.653	cell organization and biogenesis;protein metabolism;RNA metabolism OR transcription;other metabolic processes;other biological processes
P38117	electron transfer flavoprotein subunit beta [OS=Homo sapiens]	ETFB	3	45	3	12	9.761	other metabolic processes
P22570	NADPH:adrenodoxin oxidoreductase, mitochondrial [OS=Homo sapiens]	FDXR	4	34	4	9	15.181	other metabolic processes
O75955	Flotillin-1 [OS=Homo sapiens]	FLOT1	2	27	2	5	6.764	cell organization and biogenesis;stress response;transport;developmental processes;signal transduction;other biological processes
P41250	Glycine--tRNA ligase [OS=Homo sapiens]	GARS1	4	29	4	5	14.713	protein metabolism;RNA metabolism OR transcription;other metabolic processes

Q04446	1,4-alpha-glucan-branching enzyme [OS=Homo sapiens]	GBE1	3	35	3	5	8.395	other metabolic processes;other biological processes
P57678	Gem-associated protein 4 [OS=Homo sapiens]	GEMIN4	2	17	2	1	8.142	cell organization and biogenesis;RNA metabolism OR transcription;other metabolic processes
Q06210	glutamine--fructose-6-phosphate aminotransferase [isomerizing] 1 [OS=Homo sapiens]	GFPT1	7	79	7	13	24.928	protein metabolism;other metabolic processes;other biological processes
Q8ND71	GTPase IMAP family member 8 [OS=Homo sapiens]	GIMAP8	2	19	2	4	7.168	other biological processes
P07203	Glutathione peroxidase 1 [OS=Homo sapiens]	GPX1	4	42	4	25	17.016	cell cycle OR cell proliferation;cell organization and biogenesis;protein metabolism;other metabolic processes;stress response;developmental processes;signal transduction;other biological processes
P35914	Hydroxymethylglutaryl-CoA lyase, mitochondrial [OS=Homo sapiens]	HMGCL	2	29	2	6	7.69	cell organization and biogenesis;other metabolic processes;stress response;developmental processes;other biological processes
Q99729	Isoform 2 of Heterogeneous nuclear ribonucleoprotein A/B [OS=Homo sapiens]	HNRNPAB	2	33	1	7	11.462	
P07910	Heterogeneous nuclear ribonucleoproteins C1/C2 [OS=Homo sapiens]	HNRNPC	3	14	3	11	7.94	RNA metabolism OR transcription;other metabolic processes;other biological processes
P14866	Heterogeneous nuclear ribonucleoprotein L [OS=Homo sapiens]	HNRNPL	2	34	2	5	9.834	RNA metabolism OR transcription;other metabolic processes;other biological processes
Q1KMD3	heterogeneous nuclear ribonucleoprotein U-like protein 2 [OS=Homo sapiens]	HNRNPUL2	5	61	5	7	25.467	
Q16666	gamma-interferon-inducible protein 16 [OS=Homo sapiens]	IFI16	3	39	3	4	12.22	cell organization and biogenesis;protein metabolism;other metabolic processes;stress response;developmental

								processes;signal transduction;other biological processes
Q70UQ0	Inhibitor of nuclear factor kappa-B kinase-interacting protein [OS=Homo sapiens]	IKBIP	3	39	3	8	13.348	other biological processes
Q96P70	Importin-9 [OS=Homo sapiens]	IPO9	3	23	3	4	13.692	transport
Q14573	Inositol 1,4,5-trisphosphate receptor type 3 [OS=Homo sapiens]	ITPR3	3	28	3	1	9.312	transport;signal transduction;other biological processes
Q15046	Lysine--tRNA ligase [OS=Homo sapiens]	KARS1	5	48	5	8	19.229	protein metabolism;RNA metabolism OR transcription;other metabolic processes;signal transduction;other biological processes
P09960	leukotriene A-4 hydrolase [OS=Homo sapiens]	LTA4H	5	51	5	8	29.019	protein metabolism;other metabolic processes
P23368	NAD-dependent malic enzyme, mitochondrial [OS=Homo sapiens]	ME2	2	26	2	4	9.83	other metabolic processes;other biological processes
Q14764	major vault protein [OS=Homo sapiens]	MVP	11	75	11	15	47.226	transport;signal transduction;other biological processes
O94832	Unconventional myosin-Id [OS=Homo sapiens]	MYO1D	8	55	7	8	24.3	cell organization and biogenesis;transport;developmental processes;other biological processes
Q9UM54	Isoform 1 of Unconventional myosin-VI [OS=Homo sapiens]	MYO6	4	26	4	4	15.719	
P40261	Nicotinamide N-methyltransferase [OS=Homo sapiens]	NNMT	2	29	2	7	8.883	other metabolic processes;developmental processes;other biological processes
P46459	Vesicle-fusing ATPase [OS=Homo sapiens]	NSF	4	38	4	6	14.502	cell organization and biogenesis;transport;other biological processes
Q8N1F7	Nuclear pore complex protein Nup93 [OS=Homo sapiens]	NUP93	5	26	5	6	12.593	cell organization and biogenesis;other metabolic processes;transport;signal transduction;other biological processes
P22059	Oxysterol-binding protein 1 [OS=Homo sapiens]	OSBP	3	25	3	5	13.585	cell organization and biogenesis;other metabolic

								processes;transport;other biological processes
Q13177	Serine/threonine-protein kinase PAK 2 [OS=Homo sapiens]	PAK2	3	28	3	7	10.556	cell organization and biogenesis;protein metabolism;other metabolic processes;developmental processes;signal transduction;other biological processes
O00408	cGMP-dependent 3',5'-cyclic phosphodiesterase [OS=Homo sapiens]	PDE2A	4	50	4	5	18.11	other metabolic processes;developmental processes;signal transduction;other biological processes
Q14554	Protein disulfide-isomerase A5 [OS=Homo sapiens]	PDIA5	4	82	4	7	17.309	other metabolic processes;other biological processes
P17858	ATP-dependent 6-phosphofructokinase, liver type [OS=Homo sapiens]	PFKL	5	126	3	9	37.601	other metabolic processes;other biological processes
Q6PCE3	glucose 1,6-bisphosphate synthase [OS=Homo sapiens]	PGM2L1	5	51	5	9	15.279	other metabolic processes
Q92508	Piezo-type mechanosensitive ion channel component 1 [OS=Homo sapiens]	PIEZO1	7	33	7	4	26.49	transport;other biological processes
O60568	Procollagen-lysine,2-oxoglutarate 5-dioxygenase 3 [OS=Homo sapiens]	PLOD3	4	40	4	5	18.912	cell organization and biogenesis;protein metabolism;other metabolic processes;developmental processes;other biological processes
Q13131	5'-AMP-activated protein kinase catalytic subunit alpha-1 [OS=Homo sapiens]	PRKAA1	2	54	2	5	12.361	cell-cell signaling;protein metabolism;other metabolic processes;stress response;transport;signal transduction;other biological processes
O14818	Proteasome subunit alpha type-7 [OS=Homo sapiens]	PSMA7	3	53	3	14	16.388	protein metabolism;other metabolic processes
P62191	26S proteasome regulatory subunit 4 [OS=Homo sapiens]	PSMC1	4	50	3	13	19.785	protein metabolism;other metabolic processes;other biological processes
P43686	26S proteasome regulatory subunit 6B [OS=Homo sapiens]	PSMC4	4	41	4	13	16.955	protein metabolism;other metabolic processes;developmental processes;other biological processes

P62333	26S proteasome regulatory subunit 10B [OS=Homo sapiens]	PSMC6	4	27	4	12	15.029	protein metabolism;other metabolic processes;stress response;other biological processes
O00232	26s proteasome non-atpase regulatory subunit 12 [OS=Homo sapiens]	PSMD12	4	26	4	10	11.278	protein metabolism;other metabolic processes
O00487	26S proteasome non-ATPase regulatory subunit 14 [OS=Homo sapiens]	PSMD14	3	20	3	9	7.666	protein metabolism;DNA metabolism;other metabolic processes;stress response;other biological processes
Q13200	26S proteasome non-ATPase regulatory subunit 2 [OS=Homo sapiens]	PSMD2	8	113	8	8	36.21	protein metabolism;other metabolic processes;other biological processes
P26599	Polypyrimidine tract-binding protein 1 [OS=Homo sapiens]	PTBP1	4	58	4	9	17.976	RNA metabolism OR transcription;other metabolic processes;other biological processes
P18031	Tyrosine-protein phosphatase non-receptor type 1 [OS=Homo sapiens]	PTPN1	3	33	3	7	15.74	cell organization and biogenesis;protein metabolism;other metabolic processes;stress response;signal transduction;other biological processes
O95716	ras-related protein Rab-3D [OS=Homo sapiens]	RAB3D	4	34	3	16	16.261	protein metabolism;other metabolic processes;transport;other biological processes
P15153	Ras-related C3 botulinum toxin substrate 2 [OS=Homo sapiens]	RAC2	5	181	2	27	27.539	cell adhesion;cell organization and biogenesis;transport;developmental processes;signal transduction;other biological processes
P0DJD0	RANBP2-like and GRIP domain-containing protein 1 [OS=Homo sapiens]	RGPD1	2	21	2	1	7.395	transport;other biological processes
P62847	40S ribosomal protein S24 [OS=Homo sapiens]	RPS24	3	48	3	26	18.627	protein metabolism;RNA metabolism OR transcription;other metabolic processes;developmental processes;other biological processes
P62081	40S ribosomal protein S7 [OS=Homo sapiens]	RPS7	4	35	4	19	12.753	protein metabolism;RNA metabolism OR transcription;other metabolic processes;developmental processes;other biological processes

P10301	Ras-related protein R-Ras [OS=Homo sapiens]	RRAS	2	41	1	10	7.281	developmental processes;signal transduction;other biological processes
Q9P2E9	Ribosome-binding protein 1 [OS=Homo sapiens]	RRBP1	3	22	3	3	13.588	
O95197	Reticulon-3 [OS=Homo sapiens]	RTN3	2	37	2	2	8.876	cell organization and biogenesis;transport;other biological processes
Q9Y230	RuvB-like 2 [OS=Homo sapiens]	RUVBL2	5	36	4	11	23.121	cell organization and biogenesis;protein metabolism;DNA metabolism;other metabolic processes;stress response;other biological processes
P49591	Serine--tRNA ligase, cytoplasmic [OS=Homo sapiens]	SARS1	6	45	6	10	19.271	protein metabolism;RNA metabolism OR transcription;other metabolic processes;other biological processes
Q9Y3A5	Ribosome maturation protein SBDS [OS=Homo sapiens]	SBDS	2	44	2	7	7.425	cell cycle OR cell proliferation;cell organization and biogenesis;RNA metabolism OR transcription;other metabolic processes;developmental processes;other biological processes
P67812	Signal peptidase complex catalytic subunit SEC11A [OS=Homo sapiens]	SEC11A	2	54	2	9	8.084	protein metabolism;other metabolic processes
P53992	Protein transport protein sec24c [OS=Homo sapiens]	SEC24C	6	38	6	6	21.984	cell organization and biogenesis;transport;developmental processes
O94979	Protein transport protein Sec31A [OS=Homo sapiens]	SEC31A	6	21	6	5	18.636	cell organization and biogenesis;transport;other biological processes
Q6NUK1	Calcium-binding mitochondrial carrier protein SCaMC-1 [OS=Homo sapiens]	SLC25A24	3	40	3	7	11.446	stress response;transport;other biological processes
Q8NEW0	Zinc transporter 7 [OS=Homo sapiens]	SLC30A7	3	37	3	8	14.44	transport;other biological processes
Q15005	Signal peptidase complex subunit 2 [OS=Homo sapiens]	SPCS2	2	19	2	7	7.274	protein metabolism;other metabolic processes;transport
O15270	Serine palmitoyltransferase 2 [OS=Homo sapiens]	SPTLC2	4	17	4	7	15.178	other metabolic processes;developmental

								processes;other biological processes
P30626	Sorcin [OS=Homo sapiens]	SRI	2	41	2	11	9.89	transport;developmental processes;signal transduction;other biological processes
P19623	spermidine synthase [OS=Homo sapiens]	SRM	6	53	6	23	19.942	other metabolic processes;other biological processes
Q13247	Serine/arginine-rich splicing factor 6 [OS=Homo sapiens]	SRSF6	2	32	2	5	7.952	cell organization and biogenesis;RNA metabolism OR transcription;other metabolic processes;other biological processes
Q86WV6	Stimulator of interferon genes protein [OS=Homo sapiens]	STING1	2	29	2	7	12.262	cell organization and biogenesis;other metabolic processes;stress response;other biological processes
P31948	stress-induced-phosphoprotein 1 [OS=Homo sapiens]	STIP1	2	47	2	4	10.909	other biological processes
Q8TCJ2	Dolichyl-diphosphooligosaccharide--protein glycosyltransferase subunit STT3B [OS=Homo sapiens]	STT3B	4	54	4	5	15.004	protein metabolism;other metabolic processes;stress response
P53597	Succinate--CoA ligase [ADP/GDP-forming] subunit alpha, mitochondrial [OS=Homo sapiens]	SUCLG1	2	21	2	7	9.703	other metabolic processes
Q03518	Antigen peptide transporter 1 [OS=Homo sapiens]	TAP1	3	39	3	5	16.089	stress response;transport;other biological processes
Q03519	antigen peptide transporter 2 [OS=Homo sapiens]	TAP2	3	24	3	4	11.299	transport;other biological processes
Q13488	V-type proton ATPase 116 kDa subunit a isoform 3 [OS=Homo sapiens]	TCIRG1	3	34	3	4	15.268	cell cycle OR cell proliferation;cell organization and biogenesis;protein metabolism;other metabolic processes;stress response;transport;developmental processes;other biological processes
P67936	Tropomyosin alpha-4 chain [OS=Homo sapiens]	TPM4	4	27	4	15	19.12	cell organization and biogenesis;other biological processes

P10155	60 kDa SS-A/Ro ribonucleoprotein [OS=Homo sapiens]	TROVE2	2	27	2	4	9.206	cell organization and biogenesis;RNA metabolism OR transcription;other metabolic processes;developmental processes;signal transduction;other biological processes
Q9Y5S1	Transient receptor potential cation channel subfamily V member 2 [OS=Homo sapiens]	TRPV2	7	50	7	11	29.879	cell organization and biogenesis;stress response;transport;developmental processes;other biological processes
Q3KQV9	UDP-N-acetylhexosamine pyrophosphorylase-like protein 1 [OS=Homo sapiens]	UAP1L1	2	38	2	5	7.659	other metabolic processes
Q9H3U1	Isoform 2 of Protein unc-45 homolog A [OS=Homo sapiens]	UNC45A	3	49	3	3	13.425	
O60763	General vesicular transport factor p115 [OS=Homo sapiens]	USO1	9	100	9	11	38.484	cell organization and biogenesis;transport;signal transduction;other biological processes
Q93008	Isoform 2 of Probable ubiquitin carboxyl-terminal hydrolase FAF-X [OS=Homo sapiens]	USP9X	7	56	7	3	23.73	cell cycle OR cell proliferation;cell organization and biogenesis;protein metabolism;other metabolic processes;developmental processes;signal transduction;other biological processes
Q709C8	Vacuolar protein sorting-associated protein 13C [OS=Homo sapiens]	VPS13C	7	43	7	3	25.686	cell organization and biogenesis;transport;other biological processes
O75436	Vacuolar protein sorting-associated protein 26A [OS=Homo sapiens]	VPS26A	2	30	2	9	7.773	transport;other biological processes
P07947	tyrosine-protein kinase Yes [OS=Homo sapiens]	YES1	3	72	1	5	14.658	protein metabolism;other metabolic processes;stress response;transport;signal transduction;other biological processes
P31946	14-3-3 protein beta/alpha [OS=Homo sapiens]	YWHAB	8	364	5	30	46.815	transport;other biological processes
P27348	14-3-3 protein theta [OS=Homo sapiens]	YWHAQ	4	319	2	21	27.469	transport;signal transduction;other biological processes

Table S4. Proteins belonging to the enriched biological process "neutrophil-mediated immunity" as determined by over-representation analysis using WebGestalt.

Gene symbol	Accession	Gene Name	Entrez Gene ID
ACAA1	P09110	acetyl-CoA acyltransferase 1	30
ACLY	P53396	ATP citrate lyase	47
AP2A2	O94973	adaptor related protein complex 2 subunit alpha 2	161
APRT	P07741	adenine phosphoribosyltransferase	353
ARL8A	Q96BM9	ADP ribosylation factor like GTPase 8A	127829
ATAD3B	Q5T9A4	ATPase family, AAA domain containing 3B	83858
CAND1	Q86VP6	cullin associated and neddylation dissociated 1	55832
CAPN1	P07384	calpain 1	823
CAT	P04040	catalase	847
CCT2	P78371	chaperonin containing TCP1 subunit 2	10576
CCT8	P50990	chaperonin containing TCP1 subunit 8	10694
COPB1	P53618	coatomer protein complex subunit beta 1	1315
CYFIP1	Q7L576	cytoplasmic FMR1 interacting protein 1	23191
DNAJC3	Q13217	DnaJ heat shock protein family (Hsp40) member C3	5611
DYNLL1	P63167	dynein light chain LC8-type 1	8655
ERP44	Q9BS26	endoplasmic reticulum protein 44	23071
GDI2	P50395	GDP dissociation inhibitor 2	2665
JUP	P14923	junction plakoglobin	3728
KPNB1	Q14974	karyopherin subunit beta 1	3837
LTA4H	P09960	leukotriene A4 hydrolase	4048
MAGT1	Q9H0U3	magnesium transporter 1	84061
MVP	Q14764	major vault protein	9961
PFKL	P17858	phosphofructokinase, liver type	5211
PGM2	Q96G03	phosphoglucomutase 2	55276
PSMC2	P35998	proteasome 26S subunit, ATPase 2	5701
PSMC3	P17980	proteasome 26S subunit, ATPase 3	5702
PSMD1	Q99460	proteasome 26S subunit, non-ATPase 1	5707
PSMD12	O00232	proteasome 26S subunit, non-ATPase 12	5718
PSMD13	Q9UNM6	proteasome 26S subunit, non-ATPase 13	5719
PSMD14	O00487	proteasome 26S subunit, non-ATPase 14	10213
PSMD2	Q13200	proteasome 26S subunit, non-ATPase 2	5708
PSMD3	O43242	proteasome 26S subunit, non-ATPase 3	5709
PYGB	P11216	glycogen phosphorylase B	5834
RAB3D	O95716	RAB3D, member RAS oncogene family	9545
SERPINB6	P35237	serpin family B member 6	5269
SPTAN1	Q13813	spectrin alpha, non-erythrocytic 1	6709
SURF4	O15260	surfeit 4	6836

TCIRG1	Q13488	T cell immune regulator 1, ATPase H+ transporting V0 subunit a3	10312
TMEM173	Q86WV6	transmembrane protein 173	340061
VCL	P18206	vinculin	7414
XRCC6	P12956	X-ray repair cross complementing 6	2547

Table S5. Proteins belonging to the enriched biological process "cell redox homeostasis" as determined by over-representation analysis using WebGestalt.

Gene symbol	Accession	Gene Name	Entrez Gene ID
DNAJC10	Q8IXB1	DnaJ heat shock protein family (Hsp40) member C10	54431
ERP44	Q9BS26	endoplasmic reticulum protein 44	23071
GPX1	P07203	glutathione peroxidase 1	2876
NNT	Q13423	nicotinamide nucleotide transhydrogenase	23530
P4HB	P07237	prolyl 4-hydroxylase subunit beta	5034
PDIA4	P13667	protein disulfide isomerase family A member 4	9601
PDIA5	Q14554	protein disulfide isomerase family A member 5	10954
PRDX2	P32119	peroxiredoxin 2	7001
PRDX5	P30044	peroxiredoxin 5	25824

Table S6. Proteins belonging to the enriched KEGG pathway "adherens junction" as determined by over-representation analysis using WebGestalt.

Gene symbol	Accession	Gene Name	Entrez Gene ID
ACTN1	P12814	actinin alpha 1	87
ACTN4	O43707	actinin alpha 4	81
CDC42	P60953	cell division cycle 42	998
CSNK2A2	P19784	casein kinase 2 alpha 2	1459
CTNNA1	P35221	catenin alpha 1	1495
CTNNB1	P35222	catenin beta 1	1499
CTNND1	O60716	catenin delta 1	1500
PTPN1	P18031	protein tyrosine phosphatase, non-receptor type 1	5770
RAC2	P15153	Rac family small GTPase 2	5880
VCL	P18206	vinculin	7414
YES1	P07947	YES proto-oncogene 1, Src family tyrosine kinase	7525

Table S7: List of 327 proteins modified by 2-ClHDyA in HLMVEC including those also modified by HDyA

Accession	Description	Gene Symbol	# Peptides	# PSMs	# Unique Peptides	Coverage [%]	Sum PEP Score	Biological Process
P09110-1	3-ketoacyl-CoA thiolase, peroxisomal [OS=Homo sapiens]	ACAA1	3	22	3	13	22.974	other metabolic processes
P49748-1	Very long-chain specific acyl-CoA dehydrogenase, mitochondrial [OS=Homo sapiens]	ACADVL	5	92	5	8	20.94	other metabolic processes;stress response;other biological processes
P53396-1	ATP-citrate synthase [OS=Homo sapiens]	ACLY	20	631	20	21	135.424	other metabolic processes
Q99798	Aconitate hydratase, mitochondrial [OS=Homo sapiens]	ACO2	7	69	7	11	33.222	other metabolic processes;stress response;developmental processes
O00154-4	Isoform 4 of Cytosolic acyl coenzyme A thioester hydrolase [OS=Homo sapiens]	ACOT7	6	75	6	23	28.271	
Q9Y305-1	Acyl-coenzyme A thioesterase 9, mitochondrial [OS=Homo sapiens]	ACOT9	5	124	5	12	27.019	other metabolic processes
O95573	long-chain-fatty-acid--CoA ligase 3 [OS=Homo sapiens]	ACSL3	6	27	6	9	21.406	cell organization and biogenesis;other metabolic processes;transport;developmental processes;other biological processes
P12814-1	Alpha-actinin-1 [OS=Homo sapiens]	ACTN1	33	1554	21	42	254.602	cell adhesion;cell organization and biogenesis;developmental processes;other biological processes
O43707	Alpha-actinin-4 [OS=Homo sapiens]	ACTN4	28	1102	16	39	205.348	transport;signal transduction;other biological processes
P35611-1	Alpha-adducin [OS=Homo sapiens]	ADD1	5	52	5	10	18.85	cell organization and biogenesis;other biological processes
P11766	alcohol dehydrogenase class-3 [OS=Homo sapiens]	ADH5	2	19	2	7	5.625	protein metabolism;other metabolic processes;stress response;other biological processes
P30566	adenylosuccinate lyase [OS=Homo sapiens]	ADSL	2	48	2	4	11.027	other metabolic processes;stress response;other biological processes

P23526-1	Adenosylhomocysteinase [OS=Homo sapiens]	AHCY	8	65	8	21	32.366	other metabolic processes;stress response;other biological processes
Q09666-1	Neuroblast differentiation-associated protein AHNAK [OS=Homo sapiens]	AHNAK	6	94	6	3	27.183	other biological processes
Q13155	aminoacyl tRNA synthase complex-interacting multifunctional protein 2 [OS=Homo sapiens]	AIMP2	2	60	2	7	12.606	cell organization and biogenesis;protein metabolism;other metabolic processes;developmental processes;other biological processes
P00568	Adenylate kinase isoenzyme 1 [OS=Homo sapiens]	AK1	2	27	2	12	10.902	other metabolic processes
P54886	delta-1-pyrroline-5-carboxylate synthase [OS=Homo sapiens]	ALDH18A1	6	92	6	8	31.567	other metabolic processes
P05091	Aldehyde dehydrogenase, mitochondrial [OS=Homo sapiens]	ALDH2	8	238	6	17	40.781	other metabolic processes
P09525	annexin A4 [OS=Homo sapiens]	ANXA4	4	28	3	13	15.159	signal transduction;other biological processes
P20073-1	Annexin A7 [OS=Homo sapiens]	ANXA7	2	22	2	4	6.166	cell organization and biogenesis;other metabolic processes;other biological processes
O43747-1	AP-1 complex subunit gamma-1 [OS=Homo sapiens]	AP1G1	3	22	3	3	8.547	cell organization and biogenesis;transport;other biological processes
O95782	AP-2 complex subunit alpha-1 [OS=Homo sapiens]	AP2A1	7	117	4	7	30.12	transport;other biological processes
O94973	AP-2 complex subunit alpha-2 [OS=Homo sapiens]	AP2A2	6	64	3	6	26.076	transport
P63010-1	AP-2 complex subunit beta [OS=Homo sapiens]	Ap2b1; AP2B1; LOC101351101; LOC111151383	9	149	5	9	33.512	cell organization and biogenesis;other metabolic processes;transport;developmental processes;other biological processes
Q96CW1	AP-2 complex subunit mu [OS=Homo sapiens]	AP2M1	6	62	6	13	25.612	cell organization and biogenesis;other metabolic processes;transport;other biological processes
O00203-1	AP-3 complex subunit beta-1 [OS=Homo sapiens]	AP3B1	3	23	3	3	15.351	cell organization and biogenesis;protein metabolism;RNA

								metabolism OR transcription;other metabolic processes;stress response;transport;developmental processes;signal transduction;other biological processes
O14617-1	AP-3 complex subunit delta-1 [OS=Homo sapiens]	AP3D1	2	41	2	2	7.67	cell organization and biogenesis;transport;other biological processes
Q9HDC9	Adipocyte plasma membrane-associated protein [OS=Homo sapiens]	APMAP	2	42	2	6	10.787	other metabolic processes
P07741-1	Adenine phosphoribosyltransferase [OS=Homo sapiens]	APRT	4	85	4	24	21.026	other metabolic processes;transport;developmental processes;other biological processes
P48444	Coatomer subunit delta [OS=Homo sapiens]	ARCN1	7	103	7	13	35.53	transport;developmental processes;other biological processes
P61204	ADP-ribosylation factor 3 [OS=Homo sapiens]	ARF3	5	185	3	30	40.7	transport
Q07960	rho GTPase-activating protein 1 [OS=Homo sapiens]	ARHGAP1	3	42	3	6	9.008	transport;signal transduction;other biological processes
P40616	ADP-ribosylation factor-like protein 1 [OS=Homo sapiens]	ARL1	3	37	3	19	13.498	cell organization and biogenesis;other metabolic processes;transport;other biological processes
Q96BM9	ADP-ribosylation factor-like protein 8A [OS=Homo sapiens]	ARL8A	4	64	1	20	18.099	cell cycle OR cell proliferation;transport;other biological processes
Q92747	Actin-related protein 2/3 complex subunit 1A [OS=Homo sapiens]	ARPC1A	2	55	1	4	6.947	cell organization and biogenesis
O15143	Actin-related protein 2/3 complex subunit 1B [OS=Homo sapiens]	ARPC1B	6	162	5	17	28.939	cell organization and biogenesis;other biological processes
O15145	Actin-related protein 2/3 complex subunit 3 [OS=Homo sapiens]	ARPC3	3	53	3	16	17.182	cell organization and biogenesis;other biological processes
Q5T9A4	ATPase family AAA domain-containing protein 3B [OS=Homo sapiens]	ATAD3B	3	57	3	4	16.992	cell organization and biogenesis

Q6DD88	Atlastin-3 [OS=Homo sapiens]	ATL3	3	50	3	9	24.845	cell organization and biogenesis;other biological processes
Q9HD20	manganese-transporting ATPase 13A1 [OS=Homo sapiens]	ATP13A1	3	29	3	3	12.426	transport;other biological processes
P16615	Sarcoplasmic/endoplasmic reticulum calcium ATPase 2 [OS=Homo sapiens]	ATP2A2	12	167	12	15	75.944	cell adhesion;cell organization and biogenesis;other metabolic processes;stress response;transport;signal transduction;other biological processes
P23634-1	Plasma membrane calcium-transporting ATPase 4 [OS=Homo sapiens]	ATP2B4	7	48	7	7	29.056	stress response;transport;developmental processes;other biological processes
P36542-1	ATP synthase subunit gamma, mitochondrial [OS=Homo sapiens]	ATP5F1C	4	29	4	13	16.924	other metabolic processes;transport
P48047	ATP synthase subunit O, mitochondrial [OS=Homo sapiens]	ATP5PO	4	65	4	21	17.526	other metabolic processes;transport
P61421	V-type proton ATPase subunit d 1 [OS=Homo sapiens]	ATP6V0D1	2	47	2	5	9.875	cell organization and biogenesis;transport;developmental processes;other biological processes
P21281	V-type proton ATPase subunit B, brain isoform [OS=Homo sapiens]	ATP6V1B2	4	77	4	10	21.052	other metabolic processes;transport;other biological processes
Q13838-1	spliceosome RNA helicase DDX39B [OS=Homo sapiens]	BAT1; DDX39B; LOC101341139	8	192	1	17	41.394	cell organization and biogenesis;RNA metabolism OR transcription;other metabolic processes;transport;developmental processes;other biological processes
P51572	B-cell receptor-associated protein 31 [OS=Homo sapiens]	BCAP31	4	35	4	14	15.543	cell organization and biogenesis;stress response;transport;signal transduction;other biological processes
P53004	Biliverdin reductase A [OS=Homo sapiens]	BLVRA	4	42	4	16	19.327	other metabolic processes

O43684	Mitotic checkpoint protein BUB3 [OS=Homo sapiens]	BUB3	3	41	3	11	13.755	cell cycle OR cell proliferation;cell organization and biogenesis;signal transduction;other biological processes
P27708	CAD protein [OS=Homo sapiens]	CAD	9	85	9	5	32.583	protein metabolism;other metabolic processes;stress response;transport;developmental processes;other biological processes
Q86VP6-1	cullin-associated nedd8-dissociated protein 1 [OS=Homo sapiens]	CAND1; LOC111142147	17	198	17	15	79.588	cell organization and biogenesis;protein metabolism;other metabolic processes;other biological processes
P27824	Calnexin [OS=Homo sapiens]	CANX	3	26	3	6	13.919	stress response;transport;signal transduction;other biological processes
P40121	Macrophage-capping protein [OS=Homo sapiens]	CAPG	2	24	2	6	10.047	cell organization and biogenesis;developmental processes;other biological processes
P07384	Calpain-1 catalytic subunit [OS=Homo sapiens]	CAPN1	11	229	11	14	49.108	protein metabolism;other metabolic processes;developmental processes;other biological processes
P04040	catalase [OS=Homo sapiens]	CAT	4	34	4	9	23.855	protein metabolism;other metabolic processes;stress response;developmental processes;other biological processes
Q03135	Caveolin-1 [OS=Homo sapiens]	CAV1	5	623	5	23	41.252	cell-cell signaling;cell organization and biogenesis;other metabolic processes;stress response;transport;developmental processes;signal transduction;other biological processes
Q6NZI2-1	caveolae-associated protein 1 [OS=Homo sapiens]	CAVIN1	4	19	4	11	16.343	RNA metabolism OR transcription;other metabolic processes;transport;other biological processes
P16152	Carbonyl reductase [NADPH] 1 [OS=Homo sapiens]	CBR1	6	51	6	30	28.538	other metabolic processes;other biological processes

P78371-1	T-complex protein 1 subunit beta [OS=Homo sapiens]	CCT2; HEL-S-100n	10	111	10	20	51.862	cell organization and biogenesis;transport;other biological processes
P49368-1	T-complex protein 1 subunit gamma [OS=Homo sapiens]	CCT3	15	178	15	28	71.947	cell organization and biogenesis;transport;other biological processes
P50991	T-complex protein 1 subunit delta [OS=Homo sapiens]	CCT4	11	219	10	23	54.922	transport;other biological processes
P48643	T-complex protein 1 subunit epsilon [OS=Homo sapiens]	CCT5	7	109	7	11	24.647	transport;other biological processes
P40227-1	T-complex protein 1 subunit zeta [OS=Homo sapiens]	CCT6A	14	219	11	25	65.266	other biological processes
P50990	T-complex protein 1 subunit theta [OS=Homo sapiens]	CCT8	13	191	13	28	59.893	cell organization and biogenesis;transport;other biological processes
P60953	Cell division control protein 42 homolog [OS=Homo sapiens]	CDC42	2	105	1	11	15.258	cell-cell signaling;cell organization and biogenesis;protein metabolism;other metabolic processes;stress response;transport;developmental processes;signal transduction;other biological processes
O00299	chloride intracellular channel protein 1 [OS=Homo sapiens]	CLIC1	6	115	5	29	33.4	other metabolic processes;transport;signal transduction;other biological processes
Q9Y696	Chloride intracellular channel protein 4 [OS=Homo sapiens]	CLIC4	6	87	5	19	20.354	transport;developmental processes;other biological processes
P10909-1	Clusterin [OS=Homo sapiens]	CLU	5	76	5	15	29.634	cell cycle OR cell proliferation;cell organization and biogenesis;protein metabolism;other metabolic processes;stress response;transport;developmental processes;signal transduction;other biological processes
P21964-1	Catechol O-methyltransferase [OS=Homo sapiens]	COMT	2	13	2	7	7.85	other metabolic processes;stress response;other biological processes
P53621-1	coatomer subunit alpha [OS=Homo sapiens]	COPA	22	498	22	20	119.74	transport;signal transduction
P53618	Coatomer subunit beta [OS=Homo sapiens]	COPB1	12	129	12	15	46.234	transport

P35606	Coatomer subunit beta' [OS=Homo sapiens]	COPB2	9	196	9	9	36.43	transport
Q9Y678	Coatomer subunit gamma-1 [OS=Homo sapiens]	COPG1	9	98	9	16	42.333	transport;other biological processes
P50416	Carnitine O-palmitoyltransferase 1, liver isoform [OS=Homo sapiens]	CPT1A	4	48	4	6	14.406	other metabolic processes;transport;developmental processes;other biological processes
P19784	casein kinase II subunit alpha' [OS=Homo sapiens]	CSNK2A2	2	34	1	4	7.308	cell-cell signaling;cell cycle OR cell proliferation;protein metabolism;other metabolic processes;developmental processes;signal transduction;other biological processes
P56545	c-terminal-binding protein 2 [OS=Homo sapiens]	CTBP2	2	16	2	4	6.604	cell-cell signaling;cell organization and biogenesis;transport;other biological processes
P35221	Catenin alpha-1 [OS=Homo sapiens]	CTNNA1	3	33	3	5	11.952	cell adhesion;cell organization and biogenesis;stress response;developmental processes;other biological processes
P35222	Catenin beta-1 [OS=Homo sapiens]	CTNNB1	5	52	3	7	21.891	cell adhesion;cell-cell signaling;cell cycle OR cell proliferation;cell organization and biogenesis;protein metabolism;other metabolic processes;transport;developmental processes;signal transduction;other biological processes
O60716-1	Catenin delta-1 [OS=Homo sapiens]	CTNND1	6	71	6	7	24.429	cell adhesion;cell-cell signaling;cell organization and biogenesis;developmental processes;signal transduction;other biological processes
Q9UHQ9	NADH-cytochrome b5 reductase 1 [OS=Homo sapiens]	CYB5R1	3	71	3	9	11.109	other metabolic processes;transport
Q7L576-1	cytoplasmic FMR1-interacting protein 1 [OS=Homo sapiens]	CYFIP1	9	221	9	7	46.861	cell organization and biogenesis;developmental processes;signal transduction;other biological processes

Q14203	Dynactin subunit 1 [OS=Homo sapiens]	DCTN1	3	57	3	2	13.904	cell cycle OR cell proliferation;cell organization and biogenesis;transport;developmental processes;other biological processes
Q92499	ATP-dependent RNA helicase DDX1 [OS=Homo sapiens]	DDX1	6	54	6	9	28.532	cell organization and biogenesis;DNA metabolism;RNA metabolism OR transcription;other metabolic processes;stress response;developmental processes;other biological processes
Q92841-1	Isoform 2 of Probable ATP-dependent RNA helicase DDX17 [OS=Homo sapiens]	DDX17	16	312	10	25	80.604	
P17844	probable ATP-dependent RNA helicase DDX5 [OS=Homo sapiens]	DDX5	12	315	6	18	71.19	RNA metabolism OR transcription;other metabolic processes;developmental processes;signal transduction;other biological processes
Q9UBM7	7-dehydrocholesterol reductase [OS=Homo sapiens]	DHCR7	3	79	3	6	14.359	other metabolic processes;other biological processes
O43143	Pre-mRNA-splicing factor ATP-dependent RNA helicase DHX15 [OS=Homo sapiens]	DHX15	4	63	4	5	14.244	RNA metabolism OR transcription;other metabolic processes;other biological processes
Q9UDY4	DnaJ homolog subfamily B member 4 [OS=Homo sapiens]	DNAJB4	9	124	9	34	39.654	stress response;other biological processes
Q8IXB1-1	DnaJ homolog subfamily C member 10 [OS=Homo sapiens]	DNAJC 10	4	68	3	4	15.54	protein metabolism;other metabolic processes;stress response;signal transduction;other biological processes
Q13217	DnaJ homolog subfamily C member 3 [OS=Homo sapiens]	DNAJC 3	4	60	4	9	19.322	protein metabolism;other metabolic processes;stress response;other biological processes
P50570-1	Dynamin-2 [OS=Homo sapiens]	DNM2	6	104	6	7	26.752	cell cycle OR cell proliferation;cell organization and biogenesis;other metabolic processes;transport;developmental processes;signal transduction;other biological processes

Q9BZ29-1	Dedicator of cytokinesis protein 9 [OS=Homo sapiens]	DOCK9	7	60	7	4	28.607	signal transduction;other biological processes
Q16555-1	Dihydropyrimidinase-related protein 2 [OS=Homo sapiens]	DPYSL2	10	296	9	22	54.802	cell organization and biogenesis;other metabolic processes;transport;developmental processes;signal transduction;other biological processes
Q14195-2	Isoform LCRMP-4 of Dihydropyrimidinase-related protein 3 [OS=Homo sapiens]	DPYSL3	6	152	5	10	27.19	
P63167	Dynein light chain 1, cytoplasmic [OS=Homo sapiens]	DYNLL1	2	18	2	33	6.839	cell organization and biogenesis;other biological processes
Q15029	116 kDa U5 small nuclear ribonucleoprotein component [OS=Homo sapiens]	EFTUD 2	3	180	2	3	16.668	RNA metabolism OR transcription;other metabolic processes;other biological processes
Q9H4M9	EH domain-containing protein 1 [OS=Homo sapiens]	EHD1	5	130	3	9	22.893	cell organization and biogenesis;transport;developmental processes;other biological processes
Q9NZN4	EH domain-containing protein 2 [OS=Homo sapiens]	EHD2	10	187	8	16	33.539	cell organization and biogenesis;transport;other biological processes
Q9H223	EH domain-containing protein 4 [OS=Homo sapiens]	EHD4	13	285	11	25	72.884	cell organization and biogenesis;transport;other biological processes
P05198	Eukaryotic translation initiation factor 2 subunit 1 [OS=Homo sapiens]	EIF2S1	4	74	4	13	18.144	cell organization and biogenesis;protein metabolism;other metabolic processes;stress response;signal transduction;other biological processes
P41091	eukaryotic translation initiation factor 2 subunit 3 [OS=Homo sapiens]	EIF2S3	7	126	7	20	36.636	cell organization and biogenesis;protein metabolism;other metabolic processes;other biological processes
Q14152	Eukaryotic translation initiation factor 3 subunit A [OS=Homo sapiens]	EIF3A	8	98	8	5	32.857	cell organization and biogenesis;protein metabolism;other metabolic processes;other biological processes

B5ME19	eukaryotic translation initiation factor 3 subunit C-like protein [OS=Homo sapiens]	EIF3CL	5	157	5	6	28.244	cell organization and biogenesis;protein metabolism;other metabolic processes
O15371	Eukaryotic translation initiation factor 3 subunit D [OS=Homo sapiens]	EIF3D	3	51	3	6	15.679	cell organization and biogenesis;protein metabolism;other metabolic processes;other biological processes
P60228	Eukaryotic translation initiation factor 3 subunit E [OS=Homo sapiens]	EIF3E	4	84	4	8	14.787	cell organization and biogenesis;protein metabolism;RNA metabolism OR transcription;other metabolic processes;other biological processes
Q15717	ELAV-like protein 1 [OS=Homo sapiens]	ELAVL1	5	88	5	16	24.902	cell organization and biogenesis;transport;other biological processes
P29317	Ephrin type-A receptor 2 [OS=Homo sapiens]	EPHA2	2	44	2	2	8.495	cell adhesion;cell cycle OR cell proliferation;cell organization and biogenesis;protein metabolism;other metabolic processes;stress response;developmental processes;signal transduction;other biological processes
P07814	Bifunctional glutamate/proline--tRNA ligase [OS=Homo sapiens]	EPRS1	17	302	17	14	74.503	cell organization and biogenesis;protein metabolism;RNA metabolism OR transcription;other metabolic processes;stress response;other biological processes
Q9NZ08-1	endoplasmic reticulum aminopeptidase 1 [OS=Homo sapiens]	ERAP1	5	86	5	5	24.398	protein metabolism;other metabolic processes;developmental processes;signal transduction;other biological processes
Q6P179	Endoplasmic reticulum aminopeptidase 2 [OS=Homo sapiens]	ERAP2	4	53	4	4	16.112	protein metabolism;other metabolic processes;signal transduction;other biological processes
Q969X5	Endoplasmic reticulum-Golgi intermediate compartment protein 1 [OS=Homo sapiens]	ERGIC1	3	64	3	11	11.349	transport
O75477	erlin-1 [OS=Homo sapiens]	ERLIN1	5	96	2	16	25.842	
Q9BS26	Endoplasmic reticulum resident protein 44 [OS=Homo sapiens]	ERP44	4	37	3	9	14.152	protein metabolism;other metabolic processes;stress response;other biological processes

Q9BSJ8	Extended synaptotagmin-1 [OS=Homo sapiens]	ESYT1	8	112	8	8	34.977	cell organization and biogenesis;transport
P62495	Eukaryotic peptide chain release factor subunit 1 [OS=Homo sapiens]	ETF1	4	61	4	9	22.653	cell organization and biogenesis;protein metabolism;RNA metabolism OR transcription;other metabolic processes;other biological processes
P13804-1	Electron transfer flavoprotein subunit alpha, mitochondrial [OS=Homo sapiens]	ETFA	3	64	3	13	12.476	other metabolic processes
P38117	electron transfer flavoprotein subunit beta [OS=Homo sapiens]	ETFB	3	45	3	12	9.761	other metabolic processes
Q9NZB2-1	Constitutive coactivator of PPAR-gamma-like protein 1 [OS=Homo sapiens]	FAM120A	4	66	4	4	14.434	
P22570	NADPH:adrenodoxin oxidoreductase, mitochondrial [OS=Homo sapiens]	FDXR	4	34	4	9	15.181	other metabolic processes
Q96AC1	Fermitin family homolog 2 [OS=Homo sapiens]	FERMT2	2	37	2	5	8.746	cell adhesion;cell-cell signaling;cell organization and biogenesis;developmental processes;signal transduction;other biological processes
Q86UX7	Fermitin family homolog 3 [OS=Homo sapiens]	FERMT3	9	159	9	18	48.181	cell adhesion;cell organization and biogenesis;signal transduction;other biological processes
P07954	fumarate hydratase, mitochondrial [OS=Homo sapiens]	FH	4	55	4	10	17.535	DNA metabolism;other metabolic processes;stress response;other biological processes
Q14315	Filamin-C [OS=Homo sapiens]	FLNC	28	392	21	13	150.434	cell organization and biogenesis;other biological processes
O75955	Flotillin-1 [OS=Homo sapiens]	FLOT1	2	27	2	5	6.764	cell organization and biogenesis;stress response;transport;developmental processes;signal transduction;other biological processes
Q14697-1	Neutral alpha-glucosidase AB [OS=Homo sapiens]	GANAB; HEL-S-164nA	16	455	16	18	85.53	protein metabolism;other metabolic processes

P41250	Glycine--tRNA ligase [OS=Homo sapiens]	GARS1	4	29	4	5	14.713	protein metabolism;RNA metabolism OR transcription;other metabolic processes
Q04446	1,4-alpha-glucan-branching enzyme [OS=Homo sapiens]	GBE1	3	35	3	5	8.395	other metabolic processes;other biological processes
Q92616	eIF-2-alpha kinase activator GCN1 [OS=Homo sapiens]	GCN1	21	233	21	8	92.42	protein metabolism;other metabolic processes;stress response;other biological processes
P50395-1	Rab GDP dissociation inhibitor beta [OS=Homo sapiens]	GDI2	13	264	10	34	68.152	transport;signal transduction
P57678	Gem-associated protein 4 [OS=Homo sapiens]	GEMIN4	2	17	2	1	8.142	cell organization and biogenesis;RNA metabolism OR transcription;other metabolic processes
Q06210-1	glutamine--fructose-6-phosphate aminotransferase [isomerizing] 1 [OS=Homo sapiens]	GFPT1	7	79	7	13	24.928	protein metabolism;other metabolic processes;other biological processes
Q13630	GDP-L-fucose synthase [OS=Homo sapiens]	GFUS	2	58	2	6	8.602	cell adhesion;other metabolic processes;other biological processes
Q96F15	GTPase IMAP family member 5 [OS=Homo sapiens]	GIMAP5	3	83	3	13	18.453	
Q8ND71	GTPase IMAP family member 8 [OS=Homo sapiens]	GIMAP8	2	19	2	4	7.168	other biological processes
P00367	Glutamate dehydrogenase 1, mitochondrial [OS=Homo sapiens]	GLUD1	7	112	7	17	33.848	other metabolic processes;other biological processes
P04899-1	guanine nucleotide-binding protein G(i) subunit alpha-2 [OS=Homo sapiens]	GNAI2	8	150	3	26	33.789	cell-cell signaling;cell cycle OR cell proliferation;signal transduction;other biological processes
P07203	Glutathione peroxidase 1 [OS=Homo sapiens]	GPX1	4	42	4	25	17.016	cell cycle OR cell proliferation;cell organization and biogenesis;protein metabolism;other metabolic processes;stress response;developmental processes;signal transduction;other biological processes
Q9Y2Q3-1	Glutathione S-transferase kappa 1 [OS=Homo sapiens]	GSTK1; LOC51064	2	47	2	13	14.913	other metabolic processes;other biological processes

P69905	Hemoglobin subunit alpha [OS=Homo sapiens]	HBA1; HBA2	5	227	5	32	32.704	other metabolic processes;stress response;transport;other biological processes
Q00341-1	Vigilin [OS=Homo sapiens]	HDLBP	8	88	8	8	36.179	other metabolic processes;transport
O14818-1	Proteasome subunit alpha type-7 [OS=Homo sapiens]	HEL-S-276; PSMA7	3	53	3	14	16.388	protein metabolism;other metabolic processes
P34897-1	Serine hydroxymethyltransferase, mitochondrial [OS=Homo sapiens]	HEL-S-51e; SHMT2	9	239	9	18	46.964	cell organization and biogenesis;protein metabolism;other metabolic processes;stress response;other biological processes
P35914	Hydroxymethylglutaryl-CoA lyase, mitochondrial [OS=Homo sapiens]	HMGCL	2	29	2	6	7.69	cell organization and biogenesis;other metabolic processes;stress response;developmental processes;other biological processes
Q32P51	Heterogeneous nuclear ribonucleoprotein A1-like 2 [OS=Homo sapiens]	HNRNPA1L2	6	158	3	16	30.306	RNA metabolism OR transcription;other metabolic processes;transport
P51991-1	Heterogeneous nuclear ribonucleoprotein A3 [OS=Homo sapiens]	HNRNPA3; HNRPA3	4	89	1	7	16.674	RNA metabolism OR transcription;other metabolic processes;transport
Q99729-2	Isoform 2 of Heterogeneous nuclear ribonucleoprotein A/B [OS=Homo sapiens]	HNRNPAB	2	33	1	7	11.462	
P07910-1	Heterogeneous nuclear ribonucleoproteins C1/C2 [OS=Homo sapiens]	HNRNPC	3	14	3	11	7.94	RNA metabolism OR transcription;other metabolic processes;other biological processes
P31943	Heterogeneous nuclear ribonucleoprotein H [OS=Homo sapiens]	HNRNPH1	11	237	3	31	58.454	RNA metabolism OR transcription;other metabolic processes;other biological processes
P31942	Heterogeneous nuclear ribonucleoprotein H3 [OS=Homo sapiens]	HNRNPH3	3	54	2	8	13.121	RNA metabolism OR transcription;other metabolic processes;other biological processes
P14866	Heterogeneous nuclear ribonucleoprotein L [OS=Homo sapiens]	HNRNPL	2	34	2	5	9.834	RNA metabolism OR transcription;other metabolic

								processes;other biological processes
P52272	Heterogeneous nuclear ribonucleoprotein M [OS=Homo sapiens]	HNRNPM	13	320	13	23	80.288	RNA metabolism OR transcription;other metabolic processes;stress response
Q1KMD3	heterogeneous nuclear ribonucleoprotein U-like protein 2 [OS=Homo sapiens]	HNRNPUL2	5	61	5	7	25.467	
Q99714-1	3-hydroxyacyl-CoA dehydrogenase type-2 [OS=Homo sapiens]	HSD17B10	5	79	5	29	22.658	cell organization and biogenesis;RNA metabolism OR transcription;other metabolic processes
Q53GQ0	Very-long-chain 3-oxoacyl-CoA reductase [OS=Homo sapiens]	HSD17B12	2	51	2	4	6.883	cell organization and biogenesis;other metabolic processes;other biological processes
P37059	Estradiol 17-beta-dehydrogenase 2 [OS=Homo sapiens]	HSD17B2	5	159	5	16	38.833	other metabolic processes;developmental processes;other biological processes
P34932	Heat shock 70 kDa protein 4 [OS=Homo sapiens]	HSPA4	6	106	5	9	33.809	cell organization and biogenesis;stress response;transport
P41252	isoleucine--tRNA ligase, cytoplasmic [OS=Homo sapiens]	IARS1	10	186	10	8	46.833	protein metabolism;RNA metabolism OR transcription;other metabolic processes
P48735	Isocitrate dehydrogenase [NADP], mitochondrial [OS=Homo sapiens]	IDH2	6	86	5	15	25.938	other metabolic processes;developmental processes;other biological processes
Q16666-1	gamma-interferon-inducible protein 16 [OS=Homo sapiens]	IFI16	3	39	3	4	12.22	cell organization and biogenesis;protein metabolism;other metabolic processes;stress response;developmental processes;signal transduction;other biological processes
Q70UQ0	Inhibitor of nuclear factor kappa-B kinase-interacting protein [OS=Homo sapiens]	IKBIP	3	39	3	8	13.348	other biological processes
O00410	Importin-5 [OS=Homo sapiens]	IPO5	8	96	8	8	41.722	transport;other biological processes
Q96P70	Importin-9 [OS=Homo sapiens]	IPO9	3	23	3	4	13.692	transport

Q14573	Inositol 1,4,5-trisphosphate receptor type 3 [OS=Homo sapiens]	ITPR3	3	28	3	1	9.312	transport;signal transduction;other biological processes
P14923	Junction plakoglobin [OS=Homo sapiens]	JUP	11	107	9	15	44.25	cell adhesion;cell organization and biogenesis;developmental processes;other biological processes
Q15046	Lysine--tRNA ligase [OS=Homo sapiens]	KARS1	5	48	5	8	19.229	protein metabolism;RNA metabolism OR transcription;other metabolic processes;signal transduction;other biological processes
Q07666	KH domain-containing, RNA-binding, signal transduction-associated protein 1 [OS=Homo sapiens]	KHDRBS1	3	49	3	6	10.77	cell cycle OR cell proliferation;RNA metabolism OR transcription;other metabolic processes;signal transduction;other biological processes
P33176	Kinesin-1 heavy chain [OS=Homo sapiens]	KIF5B	5	53	5	7	25.335	cell organization and biogenesis;stress response;transport;developmental processes;other biological processes
Q14974	Importin subunit beta-1 [OS=Homo sapiens]	KPNB1	7	151	7	8	34.649	cell cycle OR cell proliferation;cell organization and biogenesis;transport;signal transduction;other biological processes
Q9P2J5	Leucine--tRNA ligase, cytoplasmic [OS=Homo sapiens]	LARS1	9	111	9	10	32.557	cell organization and biogenesis;protein metabolism;RNA metabolism OR transcription;other metabolic processes;stress response;other biological processes
P09382	Galectin-1 [OS=Homo sapiens]	LGALS1	4	77	4	40	32.869	stress response;developmental processes;other biological processes
P48059-3	Isoform 3 of LIM and senescent cell antigen-like-containing domain protein 1 [OS=Homo sapiens]	LIMS1	6	104	6	18	23.923	
Q12907	Vesicular integral-membrane protein VIP36 [OS=Homo sapiens]	LMAN2	3	61	3	8	10.449	cell organization and biogenesis;transport;other biological processes

P62847-1	40S ribosomal protein S24 [OS=Homo sapiens]	LOC100414020; Rps24; RPS24	3	48	3	26	18.627	protein metabolism;RNA metabolism OR transcription;other metabolic processes;developmental processes;other biological processes
P61019-1	Ras-related protein Rab-2A [OS=Homo sapiens]	LOC111155454; RAB2; Rab2a; RAB2A	5	75	5	31	31.404	cell organization and biogenesis;transport
Q92973-1	transportin-1 [OS=Homo sapiens]	LOC112839541; TNPO1	6	112	6	7	28.684	transport
P42704	Leucine-rich PPR motif-containing protein, mitochondrial [OS=Homo sapiens]	LRPPRC	20	227	20	15	94.596	transport;other biological processes
P09960-1	leukotriene A-4 hydrolase [OS=Homo sapiens]	LTA4H	5	51	5	8	29.019	protein metabolism;other metabolic processes
O95372	acyl-protein thioesterase 2 [OS=Homo sapiens]	LYPLA2	2	86	2	7	9.222	cell organization and biogenesis;protein metabolism;other metabolic processes;developmental processes
Q9H0U3	Magnesium transporter protein 1 [OS=Homo sapiens]	MAGT1	2	59	2	6	6.591	protein metabolism;other metabolic processes;transport;other biological processes
P23368-1	NAD-dependent malic enzyme, mitochondrial [OS=Homo sapiens]	ME2	2	26	2	4	9.83	other metabolic processes;other biological processes
P11586	C-1-tetrahydrofolate synthase, cytoplasmic [OS=Homo sapiens]	MTHFD1	13	232	13	16	60.3	
Q14764	major vault protein [OS=Homo sapiens]	MVP	11	75	11	15	47.226	transport;signal transduction;other biological processes
Q96S97	myeloid-associated differentiation marker [OS=Homo sapiens]	MYADM	2	81	2	10	17.723	cell organization and biogenesis;other biological processes
P35579-1	Myosin-9 [OS=Homo sapiens]	MYH9	29	324	26	16	110.307	cell adhesion;cell cycle OR cell proliferation;cell organization and biogenesis;protein metabolism;stress response;transport;developmental

								processes;signal transduction;other biological processes
O14950	Myosin regulatory light chain 12B [OS=Homo sapiens]	MYL12B	3	111	3	17	13.803	other biological processes
O94832	Unconventional myosin-Id [OS=Homo sapiens]	MYO1D	8	55	7	8	24.3	cell organization and biogenesis;transport;developmental processes;other biological processes
Q9UM54-1	Isoform 1 of Unconventional myosin-VI [OS=Homo sapiens]	MYO6	4	26	4	4	15.719	
Q9UJ70	N-acetyl-D-glucosamine kinase [OS=Homo sapiens]	NAGK	5	59	5	17	24.957	other metabolic processes
P40261	Nicotinamide N-methyltransferase [OS=Homo sapiens]	NNMT	2	29	2	7	8.883	other metabolic processes;developmental processes;other biological processes
Q13423	NAD(P) transhydrogenase, mitochondrial [OS=Homo sapiens]	NNT	10	199	10	11	48.691	other metabolic processes;transport;other biological processes
Q15233	Non-POU domain-containing octamer-binding protein [OS=Homo sapiens]	NONO	3	62	2	7	13.491	DNA metabolism;RNA metabolism OR transcription;other metabolic processes;stress response;other biological processes
P55786	puromycin-sensitive aminopeptidase [OS=Homo sapiens]	NPEPPS	9	146	9	10	42.713	protein metabolism;other metabolic processes;stress response
P46459	Vesicle-fusing ATPase [OS=Homo sapiens]	NSF	4	38	4	6	14.502	cell organization and biogenesis;transport;other biological processes
Q8N1F7-1	Nuclear pore complex protein Nup93 [OS=Homo sapiens]	NUP93	5	26	5	6	12.593	cell organization and biogenesis;other metabolic processes;transport;signal transduction;other biological processes
P22059	Oxysterol-binding protein 1 [OS=Homo sapiens]	OSBP	3	25	3	5	13.585	cell organization and biogenesis;other metabolic processes;transport;other biological processes
P07237	Protein disulfide-isomerase [OS=Homo sapiens]	P4HB	8	143	8	17	49.718	protein metabolism;other metabolic processes;stress response;signal transduction;other biological processes

P43034	Platelet-activating factor acetylhydrolase IB subunit alpha [OS=Homo sapiens]	PAFAH 1B1	4	61	3	13	16.235	cell-cell signaling;cell cycle OR cell proliferation;cell organization and biogenesis;other metabolic processes;transport;developmental processes;signal transduction;other biological processes
Q15102	Platelet-activating factor acetylhydrolase IB subunit gamma [OS=Homo sapiens]	PAFAH 1B3	2	53	2	8	9.352	other metabolic processes;developmental processes;other biological processes
P22234	multifunctional protein ADE2 [OS=Homo sapiens]	PAICS	5	55	5	13	19.613	other metabolic processes
Q13177	Serine/threonine-protein kinase PAK 2 [OS=Homo sapiens]	PAK2	3	28	3	7	10.556	cell organization and biogenesis;protein metabolism;other metabolic processes;developmental processes;signal transduction;other biological processes
O95340	bifunctional 3'-phosphoadenosine 5'-phosphosulfate synthase 2 [OS=Homo sapiens]	PAPSS 2	3	47	3	6	14.532	other metabolic processes;developmental processes
Q8WUM4	Programmed cell death 6-interacting protein [OS=Homo sapiens]	PDCD6I P	19	355	19	25	104.345	cell cycle OR cell proliferation;cell organization and biogenesis;protein metabolism;other metabolic processes;transport;other biological processes
O00408-1	cGMP-dependent 3',5'-cyclic phosphodiesterase [OS=Homo sapiens]	PDE2A	4	50	4	5	18.11	other metabolic processes;developmental processes;signal transduction;other biological processes
P11177	Pyruvate dehydrogenase E1 component subunit beta, mitochondrial [OS=Homo sapiens]	PDHB	4	92	4	11	15.772	other metabolic processes
P13667	Protein disulfide-isomerase A4 [OS=Homo sapiens]	PDIA4	6	65	6	12	28.366	stress response;transport;other biological processes
Q14554	Protein disulfide-isomerase A5 [OS=Homo sapiens]	PDIA5	4	82	4	7	17.309	other metabolic processes;other biological processes
P17858-1	ATP-dependent 6-phosphofructokinase, liver type [OS=Homo sapiens]	PFKL	5	126	3	9	37.601	other metabolic processes;other biological processes

Q01813	ATP-dependent 6-phosphofructokinase, platelet type [OS=Homo sapiens]	PFKP	14	296	12	21	97.938	other metabolic processes;other biological processes
Q96G03	Phosphoglucomutase-2 [OS=Homo sapiens]	PGM2	6	64	6	10	25.959	other metabolic processes
Q6PCE3	glucose 1,6-bisphosphate synthase [OS=Homo sapiens]	PGM2L1	5	51	5	9	15.279	other metabolic processes
P35232	Prohibitin [OS=Homo sapiens]	PHB	8	278	8	29	50.791	cell organization and biogenesis;protein metabolism;DNA metabolism;other metabolic processes;stress response;signal transduction;other biological processes
Q99623	Prohibitin-2 [OS=Homo sapiens]	PHB2	9	147	9	33	47.553	cell cycle OR cell proliferation;cell organization and biogenesis;other metabolic processes;stress response;transport;developmental processes;signal transduction;other biological processes
Q92508	Piezo-type mechanosensitive ion channel component 1 [OS=Homo sapiens]	PIEZO1	7	33	7	4	26.49	transport;other biological processes
Q15149-1	plectin [OS=Homo sapiens]	PLEC	43	556	43	10	187.923	cell cycle OR cell proliferation;cell organization and biogenesis;other metabolic processes;stress response;developmental processes;other biological processes
Q02809	Procollagen-lysine,2-oxoglutarate 5-dioxygenase 1 [OS=Homo sapiens]	PLOD1	6	94	6	11	25.389	protein metabolism;other metabolic processes;stress response;other biological processes
O60568	Procollagen-lysine,2-oxoglutarate 5-dioxygenase 3 [OS=Homo sapiens]	PLOD3	4	40	4	5	18.912	cell organization and biogenesis;protein metabolism;other metabolic processes;developmental processes;other biological processes
P29590	Protein PML [OS=Homo sapiens]	PML	7	129	7	8	31.31	cell cycle OR cell proliferation;cell organization and biogenesis;protein metabolism;other metabolic processes;stress response;transport;developmental

								processes;signal transduction;other biological processes
P49593	Protein phosphatase 1F [OS=Homo sapiens]	PPM1F	3	65	3	9	18.586	protein metabolism;other metabolic processes;signal transduction;other biological processes
P32119	Peroxiredoxin-2 [OS=Homo sapiens]	PRDX2	5	169	4	24	30.373	cell cycle OR cell proliferation;other metabolic processes;stress response;developmental processes;other biological processes
P30044-1	Peroxiredoxin-5, mitochondrial [OS=Homo sapiens]	PRDX5	5	87	5	30	24.002	other metabolic processes;stress response;other biological processes
Q13131	5'-AMP-activated protein kinase catalytic subunit alpha-1 [OS=Homo sapiens]	PRKAA 1	2	54	2	5	12.361	cell-cell signaling;protein metabolism;other metabolic processes;stress response;transport;signal transduction;other biological processes
Q6P2Q9	Pre-mRNA-processing-splicing factor 8 [OS=Homo sapiens]	PRPF8	8	108	8	3	34.662	cell organization and biogenesis;protein metabolism;RNA metabolism OR transcription;other metabolic processes;other biological processes
P25789	Proteasome subunit alpha type-4 [OS=Homo sapiens]	PSMA4	4	80	4	16	21.709	protein metabolism;other metabolic processes
P49721	proteasome subunit beta type-2 [OS=Homo sapiens]	PSMB2	3	88	3	16	17.934	protein metabolism;other metabolic processes;other biological processes
P28070	Proteasome subunit beta type-4 [OS=Homo sapiens]	PSMB4	3	67	3	10	13.308	protein metabolism;other metabolic processes;other biological processes
P28074-1	proteasome subunit beta type-5 [OS=Homo sapiens]	PSMB5	3	47	2	12	13.154	protein metabolism;other metabolic processes;stress response
P62191	26S proteasome regulatory subunit 4 [OS=Homo sapiens]	PSMC1	4	50	3	13	19.785	protein metabolism;other metabolic processes;other biological processes
P35998	26S proteasome regulatory subunit 7 [OS=Homo sapiens]	PSMC2	7	125	6	15	25.783	protein metabolism;other metabolic processes;other biological processes
P17980	26S proteasome regulatory subunit 6A [OS=Homo sapiens]	PSMC3	4	41	4	12	14.271	protein metabolism;other metabolic processes;other biological processes

P43686	26S proteasome regulatory subunit 6B [OS=Homo sapiens]	PSMC4	4	41	4	13	16.955	protein metabolism;other metabolic processes;developmental processes;other biological processes
P62333	26S proteasome regulatory subunit 10B [OS=Homo sapiens]	PSMC6	4	27	4	12	15.029	protein metabolism;other metabolic processes;stress response;other biological processes
Q99460	26S proteasome non-ATPase regulatory subunit 1 [OS=Homo sapiens]	PSMD1	5	69	5	6	15.894	protein metabolism;other metabolic processes;other biological processes
O00232-1	26s proteasome non-atpase regulatory subunit 12 [OS=Homo sapiens]	PSMD12	4	26	4	10	11.278	protein metabolism;other metabolic processes
Q9UNM6	26S proteasome non-ATPase regulatory subunit 13 [OS=Homo sapiens]	PSMD13	8	146	8	18	31.616	cell cycle OR cell proliferation;cell organization and biogenesis;protein metabolism;other metabolic processes
O00487	26S proteasome non-ATPase regulatory subunit 14 [OS=Homo sapiens]	PSMD14	3	20	3	9	7.666	protein metabolism;DNA metabolism;other metabolic processes;stress response;other biological processes
Q13200	26S proteasome non-ATPase regulatory subunit 2 [OS=Homo sapiens]	PSMD2	8	113	8	8	36.21	protein metabolism;other metabolic processes;other biological processes
O43242	26S proteasome non-ATPase regulatory subunit 3 [OS=Homo sapiens]	PSMD3	7	94	7	13	30.829	protein metabolism;other metabolic processes;other biological processes
P26599	Polypyrimidine tract-binding protein 1 [OS=Homo sapiens]	PTBP1	4	58	4	9	17.976	RNA metabolism OR transcription;other metabolic processes;other biological processes
P18031	Tyrosine-protein phosphatase non-receptor type 1 [OS=Homo sapiens]	PTPN1	3	33	3	7	15.74	cell organization and biogenesis;protein metabolism;other metabolic processes;stress response;signal transduction;other biological processes
Q92626	peroxidasin homolog [OS=Homo sapiens]	PXDN	15	344	15	11	84.863	cell organization and biogenesis;other metabolic processes;stress response;other biological processes
P11216	Glycogen phosphorylase, brain form [OS=Homo sapiens]	PYGB	7	228	4	11	38.658	other metabolic processes

P47897	glutamine--tRNA ligase [OS=Homo sapiens]	QARS1	12	210	12	16	48.57	protein metabolism;RNA metabolism OR transcription;other metabolic processes;developmental processes;other biological processes
P62820	Ras-related protein Rab-1A [OS=Homo sapiens]	RAB1A	4	103	2	21	17.886	cell adhesion;cell-cell signaling;cell organization and biogenesis;other metabolic processes;stress response;transport;other biological processes
O95716	ras-related protein Rab-3D [OS=Homo sapiens]	RAB3D	4	34	3	16	16.261	protein metabolism;other metabolic processes;transport;other biological processes
P15153	Ras-related C3 botulinum toxin substrate 2 [OS=Homo sapiens]	RAC2	5	181	2	27	27.539	cell adhesion;cell organization and biogenesis;transport;developmental processes;signal transduction;other biological processes
P54136-1	arginine--tRNA ligase, cytoplasmic [OS=Homo sapiens]	RARS1	13	198	13	25	63.675	protein metabolism;RNA metabolism OR transcription;other metabolic processes
Q8TC12	Retinol dehydrogenase 11 [OS=Homo sapiens]	RDH11	2	33	2	8	7.725	other metabolic processes;other biological processes
P0DJD0	RANBP2-like and GRIP domain-containing protein 1 [OS=Homo sapiens]	RGPD1	2	21	2	1	7.395	transport;other biological processes
Q63HN8-4	Isoform 2 of E3 ubiquitin-protein ligase RNF213 [OS=Homo sapiens]	RNF213	15	128	15	3	54.589	
P10155-1	60 kDa SS-A/Ro ribonucleoprotein [OS=Homo sapiens]	RO60; TROVE 2	2	27	2	4	9.206	cell organization and biogenesis;RNA metabolism OR transcription;other metabolic processes;developmental processes;signal transduction;other biological processes
P62910	60S ribosomal protein L32 [OS=Homo sapiens]	RPL32	3	113	3	28	31.4	protein metabolism;other metabolic processes
P18077	60S ribosomal protein L35a [OS=Homo sapiens]	RPL35A	5	65	5	23	23.109	protein metabolism;RNA metabolism OR transcription;other metabolic processes;other biological processes
P46777	60S ribosomal protein L5 [OS=Homo sapiens]	RPL5	10	281	10	37	67.975	cell organization and biogenesis;protein metabolism;RNA

								metabolism OR transcription;other metabolic processes;other biological processes
P32969	60S ribosomal protein L9 [OS=Homo sapiens]	RPL9; RPL9P7 ; RPL9P8 ; RPL9P9	4	112	4	24	16.942	protein metabolism;other metabolic processes
P04844-1	Dolichyl-diphosphooligosaccharide--protein glycosyltransferase subunit 2 [OS=Homo sapiens]	RPN2	6	125	6	13	33.504	protein metabolism;other metabolic processes;other biological processes
P25398	40S ribosomal protein S12 [OS=Homo sapiens]	RPS12	4	99	4	32	24.652	protein metabolism;other metabolic processes;other biological processes
Q71UM5	40S ribosomal protein S27-like [OS=Homo sapiens]	RPS27L	3	81	3	38	19.153	cell cycle OR cell proliferation;cell organization and biogenesis;protein metabolism;other metabolic processes;stress response;signal transduction;other biological processes
P62081	40S ribosomal protein S7 [OS=Homo sapiens]	RPS7	4	35	4	19	12.753	protein metabolism;RNA metabolism OR transcription;other metabolic processes;developmental processes;other biological processes
P10301	Ras-related protein R-Ras [OS=Homo sapiens]	RRAS	2	41	1	10	7.281	developmental processes;signal transduction;other biological processes
Q9P2E9-1	Ribosome-binding protein 1 [OS=Homo sapiens]	RRBP1	3	22	3	3	13.588	
Q9Y3I0	tRNA-splicing ligase RtcB homolog [OS=Homo sapiens]	RTCB	7	55	7	14	27.406	RNA metabolism OR transcription;other metabolic processes
O95197	Reticulon-3 [OS=Homo sapiens]	RTN3	2	37	2	2	8.876	cell organization and biogenesis;transport;other biological processes
Q9Y230	RuvB-like 2 [OS=Homo sapiens]	RUVBL 2	5	36	4	11	23.121	cell organization and biogenesis;protein metabolism;DNA metabolism;other metabolic

								processes;stress response;other biological processes
P49591	Serine--tRNA ligase, cytoplasmic [OS=Homo sapiens]	SARS1	6	45	6	10	19.271	protein metabolism;RNA metabolism OR transcription;other metabolic processes;other biological processes
Q9Y3A5	Ribosome maturation protein SBDS [OS=Homo sapiens]	SBDS	2	44	2	7	7.425	cell cycle OR cell proliferation;cell organization and biogenesis;RNA metabolism OR transcription;other metabolic processes;developmental processes;other biological processes
P67812	Signal peptidase complex catalytic subunit SEC11A [OS=Homo sapiens]	SEC11A	2	54	2	9	8.084	protein metabolism;other metabolic processes
P55735-1	protein SEC13 homolog [OS=Homo sapiens]	SEC13	2	34	2	7	7.707	cell organization and biogenesis;transport;other biological processes
Q15436	protein transport protein Sec23A [OS=Homo sapiens]	SEC23A	6	126	6	9	32.762	cell organization and biogenesis;transport;other biological processes
P53992	Protein transport protein sec24c [OS=Homo sapiens]	SEC24 C	6	38	6	6	21.984	cell organization and biogenesis;transport;developmental processes
O94979	Protein transport protein Sec31A [OS=Homo sapiens]	SEC31A	6	21	6	5	18.636	cell organization and biogenesis;transport;other biological processes
P61619	Protein transport protein Sec61 subunit alpha isoform 1 [OS=Homo sapiens]	SEC61A 1	5	87	5	9	24.94	cell organization and biogenesis;stress response;transport;developmental processes
Q15019	septin-2 [OS=Homo sapiens]	SEPTIN 2	4	63	3	16	26.681	cell cycle OR cell proliferation;cell organization and biogenesis;developmental processes;signal transduction;other biological processes
P35237	serpin B6 [OS=Homo sapiens]	SERPIN B6	6	119	5	18	32.306	stress response;other biological processes
P50453	Serpin B9 [OS=Homo sapiens]	SERPIN B9	11	396	10	30	64.471	other biological processes
Q9H9B4	Sideroflexin-1 [OS=Homo sapiens]	SFXN1	4	74	3	12	23.46	other metabolic processes;transport

O15427	Monocarboxylate transporter 4 [OS=Homo sapiens]	SLC16A3	3	63	3	6	15.833	transport
P53007	Tricarboxylate transport protein, mitochondrial [OS=Homo sapiens]	SLC25A1	2	54	2	8	11.033	other metabolic processes;transport
Q02978	Mitochondrial 2-oxoglutarate/malate carrier protein [OS=Homo sapiens]	SLC25A11	4	58	4	18	23.558	transport
Q6NUK1	Calcium-binding mitochondrial carrier protein SCaMC-1 [OS=Homo sapiens]	SLC25A24	3	40	3	7	11.446	stress response;transport;other biological processes
Q8NEW0	Zinc transporter 7 [OS=Homo sapiens]	SLC30A7	3	37	3	8	14.44	transport;other biological processes
O75643-1	U5 small nuclear ribonucleoprotein 200 kDa helicase [OS=Homo sapiens]	SNRNP200	3	43	3	1	12.569	cell organization and biogenesis;RNA metabolism OR transcription;other metabolic processes
Q13425-1	Beta-2-syntrophin [OS=Homo sapiens]	SNTB2	3	67	3	7	14.638	
O60749	Sorting nexin-2 [OS=Homo sapiens]	SNX2	3	53	3	7	15.409	cell organization and biogenesis;transport
Q15005	Signal peptidase complex subunit 2 [OS=Homo sapiens]	SPCS2	2	19	2	7	7.274	protein metabolism;other metabolic processes;transport
Q13813	Spectrin alpha chain, non-erythrocytic 1 [OS=Homo sapiens]	SPTAN1	16	156	16	8	67.074	cell organization and biogenesis
O15270	Serine palmitoyltransferase 2 [OS=Homo sapiens]	SPTLC2	4	17	4	7	15.178	other metabolic processes;developmental processes;other biological processes
Q9Y6N5	Sulfide:quinone oxidoreductase, mitochondrial [OS=Homo sapiens]	SQOR	8	114	8	19	37.403	other metabolic processes
P30626-1	Sorcin [OS=Homo sapiens]	SRI	2	41	2	11	9.89	transport;developmental processes;signal transduction;other biological processes
P19623	spermidine synthase [OS=Homo sapiens]	SRM	6	53	6	23	19.942	other metabolic processes;other biological processes
Q13247	Serine/arginine-rich splicing factor 6 [OS=Homo sapiens]	SRSF6	2	32	2	5	7.952	cell organization and biogenesis;RNA metabolism OR transcription;other metabolic

								processes;other biological processes
Q86WV6	Stimulator of interferon genes protein [OS=Homo sapiens]	STING1	2	29	2	7	12.262	cell organization and biogenesis;other metabolic processes;stress response;other biological processes
P31948	stress-induced-phosphoprotein 1 [OS=Homo sapiens]	STIP1	2	47	2	4	10.909	other biological processes
P46977	Dolichyl-diphosphooligosaccharide--protein glycosyltransferase subunit STT3A [OS=Homo sapiens]	STT3A	5	211	5	7	25.658	protein metabolism;other metabolic processes
Q8TCJ2	Dolichyl-diphosphooligosaccharide--protein glycosyltransferase subunit STT3B [OS=Homo sapiens]	STT3B	4	54	4	5	15.004	protein metabolism;other metabolic processes;stress response
P53597	Succinate--CoA ligase [ADP/GDP-forming] subunit alpha, mitochondrial [OS=Homo sapiens]	SUCLG 1	2	21	2	7	9.703	other metabolic processes
O15260-1	surfeit locus protein 4 [OS=Homo sapiens]	SURF4	2	57	2	7	14.061	cell organization and biogenesis;other biological processes
P37802	Transgelin-2 [OS=Homo sapiens]	TAGLN 2	3	69	3	16	16.72	other biological processes
Q03518	Antigen peptide transporter 1 [OS=Homo sapiens]	TAP1	3	39	3	5	16.089	stress response;transport;other biological processes
Q03519-1	antigen peptide transporter 2 [OS=Homo sapiens]	TAP2	3	24	3	4	11.299	transport;other biological processes
P26639-1	Threonine--tRNA ligase, cytoplasmic [OS=Homo sapiens]	TARS1	11	96	11	16	37.105	protein metabolism;RNA metabolism OR transcription;other metabolic processes
Q13488	V-type proton ATPase 116 kDa subunit a isoform 3 [OS=Homo sapiens]	TCIRG1	3	34	3	4	15.268	cell cycle OR cell proliferation;cell organization and biogenesis;protein metabolism;other metabolic processes;stress response;transport;developmental processes;other biological processes

P57088	Transmembrane protein 33 [OS=Homo sapiens]	TMEM33	3	103	3	11	13.351	cell organization and biogenesis;stress response;other biological processes
P67936	Tropomyosin alpha-4 chain [OS=Homo sapiens]	TPM4	4	27	4	15	19.12	cell organization and biogenesis;other biological processes
Q9Y5S1	Transient receptor potential cation channel subfamily V member 2 [OS=Homo sapiens]	TRPV2	7	50	7	11	29.879	cell organization and biogenesis;stress response;transport;developmental processes;other biological processes
P68366	Tubulin alpha-4A chain [OS=Homo sapiens]	TUBA4A	14	2581	5	38	174.698	cell cycle OR cell proliferation;cell organization and biogenesis
Q13885	Tubulin beta-2A chain [OS=Homo sapiens]	TUBB2A	19	2413	3	45	211.734	cell cycle OR cell proliferation;cell organization and biogenesis;developmental processes
Q3KQV9	UDP-N-acetylhexosamine pyrophosphorylase-like protein 1 [OS=Homo sapiens]	UAP1L1	2	38	2	5	7.659	other metabolic processes
Q5JXB2	Putative ubiquitin-conjugating enzyme E2 N-like [OS=Homo sapiens]	UBE2NL	2	65	2	14	11.74	protein metabolism;other metabolic processes
O60701	UDP-glucose 6-dehydrogenase [OS=Homo sapiens]	UGDH	4	75	4	9	26.575	cell organization and biogenesis;protein metabolism;other metabolic processes;developmental processes
Q9H3U1-2	Isoform 2 of Protein unc-45 homolog A [OS=Homo sapiens]	UNC45A	3	49	3	3	13.425	
P22695	Cytochrome b-c1 complex subunit 2, mitochondrial [OS=Homo sapiens]	UQCRC2	6	97	6	19	29.157	protein metabolism;other metabolic processes;other biological processes
O60763-1	General vesicular transport factor p115 [OS=Homo sapiens]	USO1	9	100	9	11	38.484	cell organization and biogenesis;transport;signal transduction;other biological processes
Q93008-1	Isoform 2 of Probable ubiquitin carboxyl-terminal hydrolase FAF-X [OS=Homo sapiens]	USP9X	7	56	7	3	23.73	cell cycle OR cell proliferation;cell organization and biogenesis;protein metabolism;other metabolic processes;developmental processes;signal transduction;other biological processes

P26640	Valine--tRNA ligase [OS=Homo sapiens]	VARS1	8	100	8	8	34.593	protein metabolism;RNA metabolism OR transcription;other metabolic processes
P18206	Vinculin [OS=Homo sapiens]	VCL	10	114	10	10	38.461	cell adhesion;cell organization and biogenesis;developmental processes;other biological processes
Q9Y277	Voltage-dependent anion-selective channel protein 3 [OS=Homo sapiens]	VDAC3	5	88	5	23	22.76	transport;other biological processes
Q709C8	Vacuolar protein sorting-associated protein 13C [OS=Homo sapiens]	VPS13C	7	43	7	3	25.686	cell organization and biogenesis;transport;other biological processes
O75436-1	Vacuolar protein sorting-associated protein 26A [OS=Homo sapiens]	VPS26A	2	30	2	9	7.773	transport;other biological processes
Q96QK1	Vacuolar protein sorting-associated protein 35 [OS=Homo sapiens]	VPS35	6	150	6	8	33.37	cell-cell signaling;cell organization and biogenesis;transport;signal transduction;other biological processes
O76024	Wolframin [OS=Homo sapiens]	WFS1	4	95	4	4	21.135	protein metabolism;other metabolic processes;stress response;developmental processes;signal transduction;other biological processes
O14980	Exportin-1 [OS=Homo sapiens]	XPO1	7	70	7	7	21.401	transport;other biological processes
P12956	X-ray repair cross-complementing protein 6 [OS=Homo sapiens]	XRCC6	7	130	7	14	36.321	cell organization and biogenesis;DNA metabolism;other metabolic processes;stress response;developmental processes;other biological processes
P07947	tyrosine-protein kinase Yes [OS=Homo sapiens]	YES1	3	72	1	5	14.658	protein metabolism;other metabolic processes;stress response;transport;signal transduction;other biological processes
P31946	14-3-3 protein beta/alpha [OS=Homo sapiens]	YWHAB	8	364	5	30	46.815	transport;other biological processes
Q04917	14-3-3 protein eta [OS=Homo sapiens]	YWHAH	6	347	3	25	39.289	other metabolic processes;transport;developmental

								processes;signal transduction;other biological processes
P27348	14-3-3 protein theta [OS=Homo sapiens]	YWHAQ	4	319	2	21	27.469	transport;signal transduction;other biological processes

Table S8. List of 46 proteins modified only by ClHDyA in both HLMVEC and EA.hy926 cells

Accession	Description	Gene Symbol	# Peptides	# PSMs	# Unique Peptides	Coverage [%]	Sum PEP Score	Biological Process
P09110	3-ketoacyl-CoA thiolase, peroxisomal [OS=Homo sapiens]	ACAA1	3	22	3	13	22.974	other metabolic processes
O95573	long-chain-fatty-acid--CoA ligase 3 [OS=Homo sapiens]	ACSL3	6	27	6	9	21.406	cell organization and biogenesis;other metabolic processes;transport;developmental processes;other biological processes
P00568	Adenylate kinase isoenzyme 1 [OS=Homo sapiens]	AK1	2	27	2	12	10.902	other metabolic processes
P20073	Annexin A7 [OS=Homo sapiens]	ANXA7	2	22	2	4	6.166	cell organization and biogenesis;other metabolic processes;other biological processes
O94973	AP-2 complex subunit alpha-2 [OS=Homo sapiens]	AP2A2	6	64	3	6	26.076	transport
O00203	AP-3 complex subunit beta-1 [OS=Homo sapiens]	AP3B1	3	23	3	3	15.351	cell organization and biogenesis;protein metabolism;RNA metabolism OR transcription;other metabolic processes;stress response;transport;developmental processes;signal transduction;other biological processes

P40616	ADP-ribosylation factor-like protein 1 [OS=Homo sapiens]	ARL1	3	37	3	19	13.498	cell organization and biogenesis;other metabolic processes;transport;other biological processes
Q6DD88	Atlastin-3 [OS=Homo sapiens]	ATL3	3	50	3	9	24.845	cell organization and biogenesis;other biological processes
P27824	Calnexin [OS=Homo sapiens]	CANX	3	26	3	6	13.919	stress response;transport;signal transduction;other biological processes
P04040	catalase [OS=Homo sapiens]	CAT	4	34	4	9	23.855	protein metabolism;other metabolic processes;stress response;developmental processes;other biological processes
Q6NZI2	caveolae-associated protein 1 [OS=Homo sapiens]	CAVIN1	4	19	4	11	16.343	RNA metabolism OR transcription;other metabolic processes;transport;other biological processes
P21964	Catechol O-methyltransferase [OS=Homo sapiens]	COMT	2	13	2	7	7.85	other metabolic processes;stress response;other biological processes
P56545	c-terminal-binding protein 2 [OS=Homo sapiens]	CTBP2	2	16	2	4	6.604	cell-cell signaling;cell organization and biogenesis;transport;other biological processes
P35222	Catenin beta-1 [OS=Homo sapiens]	CTNNB1	5	52	3	7	21.891	cell adhesion;cell-cell signaling;cell cycle OR cell proliferation;cell organization and biogenesis;protein metabolism;other metabolic processes;transport;developmental processes;signal transduction;other biological processes
Q14152	Eukaryotic translation initiation factor 3 subunit A [OS=Homo sapiens]	EIF3A	8	98	8	5	32.857	cell organization and biogenesis;protein metabolism;other metabolic processes;other biological processes

P62495	Eukaryotic peptide chain release factor subunit 1 [OS=Homo sapiens]	ETF1	4	61	4	9	22.653	cell organization and biogenesis;protein metabolism;RNA metabolism OR transcription;other metabolic processes;other biological processes
Q04446	1,4-alpha-glucan-branching enzyme [OS=Homo sapiens]	GBE1	3	35	3	5	8.395	other metabolic processes;other biological processes
Q06210	glutamine--fructose-6-phosphate aminotransferase [isomerizing] 1 [OS=Homo sapiens]	GFPT1	7	79	7	13	24.928	protein metabolism;other metabolic processes;other biological processes
P14866	Heterogeneous nuclear ribonucleoprotein L [OS=Homo sapiens]	HNRNPL	2	34	2	5	9.834	RNA metabolism OR transcription;other metabolic processes;other biological processes
Q16666	gamma-interferon-inducible protein 16 [OS=Homo sapiens]	IFI16	3	39	3	4	12.22	cell organization and biogenesis;protein metabolism;other metabolic processes;stress response;developmental processes;signal transduction;other biological processes
Q70UQ0	Inhibitor of nuclear factor kappa-B kinase-interacting protein [OS=Homo sapiens]	IKBIP	3	39	3	8	13.348	other biological processes
Q96P70	Importin-9 [OS=Homo sapiens]	IPO9	3	23	3	4	13.692	transport
Q15046	Lysine--tRNA ligase [OS=Homo sapiens]	KARS1	5	48	5	8	19.229	protein metabolism;RNA metabolism OR transcription;other metabolic processes;signal transduction;other biological processes
P09960	leukotriene A-4 hydrolase [OS=Homo sapiens]	LTA4H	5	51	5	8	29.019	protein metabolism;other metabolic processes
Q8N1F7	Nuclear pore complex protein Nup93 [OS=Homo sapiens]	NUP93	5	26	5	6	12.593	cell organization and biogenesis;other metabolic processes;transport;signal

								transduction;other biological processes
Q13177	Serine/threonine-protein kinase PAK 2 [OS=Homo sapiens]	PAK2	3	28	3	7	10.556	cell organization and biogenesis;protein metabolism;other metabolic processes;developmental processes;signal transduction;other biological processes
Q14554	Protein disulfide-isomerase A5 [OS=Homo sapiens]	PDIA5	4	82	4	7	17.309	other metabolic processes;other biological processes
P17858	ATP-dependent 6-phosphofructokinase, liver type [OS=Homo sapiens]	PFKL	5	126	3	9	37.601	other metabolic processes;other biological processes
Q6PCE3	glucose 1,6-bisphosphate synthase [OS=Homo sapiens]	PGM2L1	5	51	5	9	15.279	other metabolic processes
O60568	Procollagen-lysine,2-oxoglutarate 5-dioxygenase 3 [OS=Homo sapiens]	PLOD3	4	40	4	5	18.912	cell organization and biogenesis;protein metabolism;other metabolic processes;developmental processes;other biological processes
P62191	26S proteasome regulatory subunit 4 [OS=Homo sapiens]	PSMC1	4	50	3	13	19.785	protein metabolism;other metabolic processes;other biological processes
P43686	26S proteasome regulatory subunit 6B [OS=Homo sapiens]	PSMC4	4	41	4	13	16.955	protein metabolism;other metabolic processes;developmental processes;other biological processes
P62333	26S proteasome regulatory subunit 10B [OS=Homo sapiens]	PSMC6	4	27	4	12	15.029	protein metabolism;other metabolic processes;stress response;other biological processes
O00487	26S proteasome non-ATPase regulatory subunit 14 [OS=Homo sapiens]	PSMD14	3	20	3	9	7.666	protein metabolism;DNA metabolism;other metabolic processes;stress response;other biological processes

Q13200	26S proteasome non-ATPase regulatory subunit 2 [OS=Homo sapiens]	PSMD2	8	113	8	8	36.21	protein metabolism;other metabolic processes;other biological processes
P53992	Protein transport protein sec24c [OS=Homo sapiens]	SEC24C	6	38	6	6	21.984	cell organization and biogenesis;transport;developmental processes
Q6NUK1	Calcium-binding mitochondrial carrier protein SCaMC-1 [OS=Homo sapiens]	SLC25A24	3	40	3	7	11.446	stress response;transport;other biological processes
P19623	spermidine synthase [OS=Homo sapiens]	SRM	6	53	6	23	19.942	other metabolic processes;other biological processes
P31948	stress-induced-phosphoprotein 1 [OS=Homo sapiens]	STIP1	2	47	2	4	10.909	other biological processes
Q8TCJ2	Dolichyl-diphosphooligosaccharide--protein glycosyltransferase subunit STT3B [OS=Homo sapiens]	STT3B	4	54	4	5	15.004	protein metabolism;other metabolic processes;stress response
P53597	Succinate--CoA ligase [ADP/GDP-forming] subunit alpha, mitochondrial [OS=Homo sapiens]	SUCLG1	2	21	2	7	9.703	other metabolic processes
Q9H3U1	Isoform 2 of Protein unc-45 homolog A [OS=Homo sapiens]	UNC45A	3	49	3	3	13.425	
O60763	General vesicular transport factor p115 [OS=Homo sapiens]	USO1	9	100	9	11	38.484	cell organization and biogenesis;transport;signal transduction;other biological processes
Q93008	Isoform 2 of Probable ubiquitin carboxyl-terminal hydrolase FAF-X [OS=Homo sapiens]	USP9X	7	56	7	3	23.73	cell cycle OR cell proliferation;cell organization and biogenesis;protein metabolism;other metabolic processes;developmental processes;signal transduction;other biological processes

O75436	Vacuolar protein sorting-associated protein 26A [OS=Homo sapiens]	VPS26A	2	30	2	9	7.773	transport;other biological processes
P31946	14-3-3 protein beta/alpha [OS=Homo sapiens]	YWHAB	8	364	5	30	46.815	transport;other biological processes

Table S9. List of 164 proteins modified by ClHDyA and HDyA in both HLMVEC and EA.hy926 cells

Accession	Description	Gene Symbol	# Peptides	# PSMs	# Unique Peptides	Coverage [%]	Sum PEP Score	Biological Process
P09110-1	3-ketoacyl-CoA thiolase, peroxisomal [OS=Homo sapiens]	ACAA1	10	131	10	38	60.632	metabolic process;transport
Q99798	Aconitate hydratase, mitochondrial [OS=Homo sapiens]	ACO2	5	18	5	10	17.453	metabolic process;response to stimulus
O00154-4	Isoform 4 of Cytosolic acyl coenzyme A thioester hydrolase [OS=Homo sapiens]	ACOT7	7	102	7	29	32.112	metabolic process
O95573	long-chain-fatty-acid--CoA ligase 3 [OS=Homo sapiens]	ACSL3	5	46	3	10	21.683	cell organization and biogenesis;metabolic process;regulation of biological process;response to stimulus;transport
P12814-1	Alpha-actinin-1 [OS=Homo sapiens]	ACTN1	30	600	18	42	171.711	cell differentiation;cell organization and biogenesis;regulation of biological process;transport
P61160-1	Actin-related protein 2 [OS=Homo sapiens]	ACTR2	7	79	7	22	39.621	cell division;cell organization and biogenesis;cellular component movement;regulation of biological process;response to stimulus;transport
P30520	adenylosuccinate synthetase isozyme 2 [OS=Homo sapiens]	ADSS	4	54	4	13	26.475	metabolic process;response to stimulus
O00116	Alkyldihydroxyacetonephosphate synthase, peroxisomal [OS=Homo sapiens]	AGPS	3	47	3	6	16.795	metabolic process
Q13155	aminoacyl tRNA synthase complex-interacting multifunctional protein 2 [OS=Homo sapiens]	AIMP2	3	21	3	17	12.218	cell death;cell differentiation;cell organization and biogenesis;development;metabolic process;regulation of biological process
P15121	aldose reductase [OS=Homo sapiens]	AKR1B1	5	92	4	11	25.171	metabolic process;regulation of biological process;response to stimulus
P49419	Alpha-aminoadipic semialdehyde dehydrogenase [OS=Homo sapiens]	ALDH7A1	5	25	5	15	17.78	metabolic process
P20073-1	Annexin A7 [OS=Homo sapiens]	ANXA7	4	48	4	10	20.365	cell differentiation;cell organization and biogenesis;cell

								proliferation;cellular homeostasis;metabolic process;regulation of biological process;response to stimulus
Q9BXS5-1	AP-1 complex subunit mu-1 [OS=Homo sapiens]	AP1M1	5	20	5	17	15.492	cell organization and biogenesis;regulation of biological process;transport
O00203-1	AP-3 complex subunit beta-1 [OS=Homo sapiens]	AP3B1	2	22	2	3	7.489	cell organization and biogenesis;cellular component movement;coagulation;regulation of biological process;transport
P48444	Coatomer subunit delta [OS=Homo sapiens]	ARCN1	6	125	6	11	32.866	response to stimulus;transport
P61204	ADP-ribosylation factor 3 [OS=Homo sapiens]	ARF3	7	86	4	51	42.394	metabolic process;regulation of biological process;response to stimulus;transport
P40616	ADP-ribosylation factor-like protein 1 [OS=Homo sapiens]	ARL1	3	46	3	19	16.567	cell organization and biogenesis;metabolic process;regulation of biological process;response to stimulus;transport
O15144	Actin-related protein 2/3 complex subunit 2 [OS=Homo sapiens]	ARPC2	4	41	4	12	14.602	cell organization and biogenesis;cellular component movement;regulation of biological process;response to stimulus
O43684	Mitotic checkpoint protein BUB3 [OS=Homo sapiens]	BUB3	4	39	4	13	17.118	cell division;cell organization and biogenesis;metabolic process;regulation of biological process
Q9Y3I0	tRNA-splicing ligase RtcB homolog [OS=Homo sapiens]	C22orf28; RTCB	7	78	7	16	39.425	metabolic process
P07384	Calpain-1 catalytic subunit [OS=Homo sapiens]	CAPN1	4	51	4	7	22.1	cell death;cell organization and biogenesis;metabolic process;regulation of biological process;transport
P52907	F-actin-capping protein subunit alpha-1 [OS=Homo sapiens]	CAPZA1	3	46	2	14	13.776	cell organization and biogenesis;cellular component movement;coagulation;defense response;regulation of biological process;response to stimulus;transport

P04040	catalase [OS=Homo sapiens]	CAT	3	22	3	9	16.407	cell differentiation;cell organization and biogenesis;metabolic process;regulation of biological process;response to stimulus;transport
Q03135	Caveolin-1 [OS=Homo sapiens]	CAV1	3	157	3	25	22.318	cell communication;cell differentiation;cell organization and biogenesis;cellular component movement;cellular homeostasis;metabolic process;regulation of biological process;response to stimulus;transport
Q16543	Hsp90 co-chaperone Cdc37 [OS=Homo sapiens]	CDC37	4	42	4	14	19.443	cell division;metabolic process;regulation of biological process;response to stimulus;transport
Q00535	Cyclin-dependent-like kinase 5 [OS=Homo sapiens]	CDK5	3	18	3	9	12.872	cell communication;cell death;cell differentiation;cell division;cell growth;cell organization and biogenesis;cell proliferation;cellular component movement;metabolic process;regulation of biological process;response to stimulus;transport
Q9Y696	Chloride intracellular channel protein 4 [OS=Homo sapiens]	CLIC4	8	46	8	43	27.706	cell differentiation;cell organization and biogenesis;cellular homeostasis;metabolic process;regulation of biological process;response to stimulus;transport
Q96KP4	cytosolic non-specific dipeptidase [OS=Homo sapiens]	CNDP2	3	15	3	10	8.701	metabolic process
Q8NBJ5	Procollagen galactosyltransferase 1 [OS=Homo sapiens]	COLGALT1; GLT25D1	5	38	5	9	21.911	
P21964-1	Catechol O-methyltransferase [OS=Homo sapiens]	COMT	4	35	4	20	22.626	cell communication;metabolic process;regulation of biological process;response to stimulus
Q99829	Copine-1 [OS=Homo sapiens]	CPNE1	4	42	4	7	17.563	cell differentiation;cell growth;metabolic process;regulation

								of biological process;response to stimulus;transport
P41240	tyrosine-protein kinase CSK [OS=Homo sapiens]	CSK	3	14	3	8	9.862	cell differentiation;cell organization and biogenesis;defense response;metabolic process;regulation of biological process;response to stimulus
P56545	c-terminal-binding protein 2 [OS=Homo sapiens]	CTBP2	2	25	1	6	9.605	cell differentiation;metabolic process;regulation of biological process
O43169	Cytochrome b5 type B [OS=Homo sapiens]	CYB5B	3	49	3	45	24.678	metabolic process;transport
P61803	Dolichyl-diphosphooligosaccharide--protein glycosyltransferase subunit DAD1 [OS=Homo sapiens]	DAD1	3	43	3	29	15.652	cell death;metabolic process;regulation of biological process;response to stimulus
Q6PI48	Aspartate--tRNA ligase, mitochondrial [OS=Homo sapiens]	DARS2	3	37	3	6	13.475	metabolic process
Q92499	ATP-dependent RNA helicase DDX1 [OS=Homo sapiens]	DDX1	7	92	7	10	34.476	cell organization and biogenesis;development;metabolic process;regulation of biological process;response to stimulus
Q9NR30-1	Nucleolar RNA helicase 2 [OS=Homo sapiens]	DDX21	6	42	6	9	21.475	cell differentiation;metabolic process;regulation of biological process;response to stimulus
P26196	Probable ATP-dependent RNA helicase DDX6 [OS=Homo sapiens]	DDX6	7	72	7	20	32.456	cell organization and biogenesis;metabolic process;regulation of biological process
P09622	Dihydrolipoyl dehydrogenase, mitochondrial [OS=Homo sapiens]	DLD	4	42	4	13	15.742	cellular homeostasis;metabolic process;regulation of biological process
P36957	Dihydrolipoyllysine-residue succinyltransferase component of 2-oxoglutarate dehydrogenase complex, mitochondrial [OS=Homo sapiens]	DLST	3	56	3	8	22.62	metabolic process
Q9UDY4	DnaJ homolog subfamily B member 4 [OS=Homo sapiens]	DNAJB4	4	44	3	17	14.628	metabolic process;regulation of biological process;response to stimulus

O00429	Dynamin-1-like protein [OS=Homo sapiens]	DNM1L	9	68	9	16	31.972	cell death;cell organization and biogenesis;regulation of biological process;response to stimulus;transport
Q12805	EGF-containing fibulin-like extracellular matrix protein 1 [OS=Homo sapiens]	EFEMP1	7	74	7	17	26.956	metabolic process;regulation of biological process;response to stimulus
Q15029	116 kDa U5 small nuclear ribonucleoprotein component [OS=Homo sapiens]	EFTUD2	2	55	1	2	9.595	cell organization and biogenesis;metabolic process;response to stimulus
Q9H4M9	EH domain-containing protein 1 [OS=Homo sapiens]	EHD1	14	212	13	41	81.472	cell organization and biogenesis;coagulation;regulation of biological process;response to stimulus;transport
Q9H223	EH domain-containing protein 4 [OS=Homo sapiens]	EHD4	7	99	7	16	33.887	cell organization and biogenesis;regulation of biological process;response to stimulus;transport
P19525	interferon-induced, double-stranded RNA-activated protein kinase [OS=Homo sapiens]	EIF2AK2	2	17	2	4	6.913	cell communication;defense response;metabolic process;regulation of biological process;response to stimulus
P41091	eukaryotic translation initiation factor 2 subunit 3 [OS=Homo sapiens]	EIF2S3	8	94	8	26	39.684	metabolic process;transport
Q14240-1	eukaryotic initiation factor 4A-II [OS=Homo sapiens]	EIF4A2	10	354	2	27	79.092	metabolic process;regulation of biological process
Q04637	eukaryotic translation initiation factor 4 gamma 1 [OS=Homo sapiens]	EIF4G1	3	16	3	2	13.446	cell organization and biogenesis;defense response;metabolic process;regulation of biological process;response to stimulus
Q6IS14	Eukaryotic translation initiation factor 5A-1-like [OS=Homo sapiens]	EIF5AL1	3	13	3	33	8.843	metabolic process;regulation of biological process;transport
P29317	Ephrin type-A receptor 2 [OS=Homo sapiens]	EPHA2	4	27	4	4	15.174	cell death;cell differentiation;cell organization and biogenesis;cell proliferation;cellular component movement;defense response;development;metabolic process;regulation of biological process;response to stimulus

P07814	Bifunctional glutamate/proline--tRNA ligase [OS=Homo sapiens]	EPRS	14	113	14	13	51.704	cell organization and biogenesis;metabolic process;regulation of biological process;response to stimulus
Q9NZ08-1	endoplasmic reticulum aminopeptidase 1 [OS=Homo sapiens]	ERAP1	7	46	7	8	22.243	cell communication;cell differentiation;metabolic process;regulation of biological process;response to stimulus
A0FGR8-1	Extended synaptotagmin-2 [OS=Homo sapiens]	ESYT2	7	48	7	10	28.763	metabolic process;transport
P62495	Eukaryotic peptide chain release factor subunit 1 [OS=Homo sapiens]	ETF1	5	60	5	14	25.537	cell organization and biogenesis;metabolic process;regulation of biological process
Q9NZB2-1	Constitutive coactivator of PPAR-gamma-like protein 1 [OS=Homo sapiens]	FAM120A	6	38	6	8	16.621	
Q96TA1	Niban-like protein 1 [OS=Homo sapiens]	FAM129B	8	106	8	12	36.864	cell differentiation;cellular component movement;metabolic process;regulation of biological process;transport
Q9BRX8-1	Redox-regulatory protein FAM213A [OS=Homo sapiens]	FAM213A	8	170	8	34	41.341	metabolic process;regulation of biological process
Q9NSD9	Phenylalanine--tRNA ligase beta subunit [OS=Homo sapiens]	FARSB	5	60	5	9	19.341	cell organization and biogenesis;metabolic process
P14324	Farnesyl pyrophosphate synthase [OS=Homo sapiens]	FDPS	2	40	2	7	9.005	metabolic process;regulation of biological process
Q13045-1	protein flightless-1 homolog [OS=Homo sapiens]	FLII	3	50	3	2	15.731	cell organization and biogenesis;development;metabolic process;regulation of biological process
P51570	galactokinase [OS=Homo sapiens]	GALK1	3	29	3	11	11.034	metabolic process
Q14697-1	Neutral alpha-glucosidase AB [OS=Homo sapiens]	GANAB	13	384	13	15	90.669	metabolic process
P62879	Guanine nucleotide-binding protein G(I)/G(S)/G(T) subunit beta-2 [OS=Homo sapiens]	GNB2	3	42	3	10	21.707	metabolic process;regulation of biological process;response to stimulus
P40939	Trifunctional enzyme subunit alpha, mitochondrial [OS=Homo sapiens]	HADHA	11	180	11	20	71.612	metabolic process;response to stimulus
Q00341-1	Vigilin [OS=Homo sapiens]	HDLBP	11	127	11	12	47.817	metabolic process;transport

Q8TCT9-1	Minor histocompatibility antigen H13 [OS=Homo sapiens]	HM13	3	26	3	12	16.831	cell organization and biogenesis;metabolic process
P31943	Heterogeneous nuclear ribonucleoprotein H [OS=Homo sapiens]	HNRNPH1	12	402	4	34	103.361	metabolic process;regulation of biological process;response to stimulus
P14866	Heterogeneous nuclear ribonucleoprotein L [OS=Homo sapiens]	HNRNPL	7	43	7	19	21.286	metabolic process;regulation of biological process
O43390-1	heterogeneous nuclear ribonucleoprotein r [OS=Homo sapiens]	HNRNPR	5	67	2	9	20.585	metabolic process;regulation of biological process
Q9NSE4	Isoleucine--tRNA ligase, mitochondrial [OS=Homo sapiens]	IARS2	5	49	5	6	23.511	metabolic process;regulation of biological process
Q16666-1	gamma-interferon-inducible protein 16 [OS=Homo sapiens]	IFI16	5	56	5	8	23.879	cell communication;cell death;cell differentiation;cell proliferation;defense response;metabolic process;regulation of biological process;response to stimulus
Q9NZI8	Insulin-like growth factor 2 mRNA-binding protein 1 [OS=Homo sapiens]	IGF2BP1	6	121	5	14	34.943	cell proliferation;regulation of biological process;transport
Q9Y6M1	Insulin-like growth factor 2 mRNA-binding protein 2 [OS=Homo sapiens]	IGF2BP2	8	102	6	18	40.57	regulation of biological process;transport
Q8TEX9-1	Importin-4 [OS=Homo sapiens]	IPO4	5	28	5	5	21.79	cell organization and biogenesis;transport
O95373	Importin-7 [OS=Homo sapiens]	IPO7	7	51	6	9	30.846	defense response;regulation of biological process;response to stimulus;transport
Q96P70	Importin-9 [OS=Homo sapiens]	IPO9	2	23	2	4	19.99	transport
Q14974	Importin subunit beta-1 [OS=Homo sapiens]	KPNB1	6	66	6	9	27.52	cell organization and biogenesis;cellular component movement;metabolic process;regulation of biological process;response to stimulus;transport
P42704	Leucine-rich PPR motif-containing protein, mitochondrial [OS=Homo sapiens]	LRPPRC	12	107	12	12	54.149	cellular component movement;metabolic process;regulation of biological process;transport

P09960-1	leukotriene A-4 hydrolase [OS=Homo sapiens]	LTA4H	6	68	6	12	44.086	metabolic process;transport
Q9UPN3	Microtubule-actin cross-linking factor 1, isoforms 1/2/3/5 [OS=Homo sapiens]	MACF1	15	128	4	2	52.118	cell organization and biogenesis;regulation of biological process;response to stimulus;transport
P46821	microtubule-associated protein 1B [OS=Homo sapiens]	MAP1B	2	32	2	2	11.527	cell growth;cell organization and biogenesis;cellular component movement;regulation of biological process;transport
P25205	DNA replication licensing factor mcm3 [OS=Homo sapiens]	MCM3	9	84	9	14	41.663	metabolic process
P33993-1	DNA replication licensing factor MCM7 [OS=Homo sapiens]	MCM7	7	47	7	13	22.492	cell proliferation;metabolic process;regulation of biological process;response to stimulus
O60502	protein O-GlcNAcase [OS=Homo sapiens]	MGEA5	3	10	3	4	11.843	metabolic process
Q96S97	myeloid-associated differentiation marker [OS=Homo sapiens]	MYADM	2	48	2	10	12.224	cell organization and biogenesis;regulation of biological process
O43776	Asparagine--tRNA ligase, cytoplasmic [OS=Homo sapiens]	NARS	5	37	5	9	22.133	metabolic process
Q8TD19	Serine/threonine-protein kinase Nek9 [OS=Homo sapiens]	NEK9	2	20	2	2	10.033	cell division;cell organization and biogenesis;metabolic process
O00567	Nucleolar protein 56 [OS=Homo sapiens]	NOP56	6	49	6	16	24.337	metabolic process
P15559-1	NAD(P)H dehydrogenase [quinone] 1 [OS=Homo sapiens]	NQO1	3	67	3	15	19.779	cell communication;metabolic process;regulation of biological process;response to stimulus
Q9BSD7	Cancer-related nucleoside-triphosphatase [OS=Homo sapiens]	NTPCR	2	25	2	14	12.919	metabolic process;response to stimulus
P04181-1	Ornithine aminotransferase, mitochondrial [OS=Homo sapiens]	OAT	5	48	5	14	21.912	cell organization and biogenesis;metabolic process
Q9UQ80	proliferation-associated protein 2G4 [OS=Homo sapiens]	PA2G4	6	45	6	18	18.858	cell proliferation;metabolic process;regulation of biological process;transport
Q99497	protein/nucleic acid deglycase DJ-1 [OS=Homo sapiens]	PARK7	6	49	6	46	21.783	cell communication;cell organization and biogenesis;defense response;metabolic process;regulation of biological

								process;response to stimulus;transport
P16284	Platelet endothelial cell adhesion molecule [OS=Homo sapiens]	PECAM 1	7	77	7	14	34.047	cell organization and biogenesis;cellular component movement;regulation of biological process;response to stimulus;transport
Q01813	ATP-dependent 6-phosphofructokinase, platelet type [OS=Homo sapiens]	PFKP	19	321	17	29	119.994	metabolic process
P35080-2	Isoform IIb of Profilin-2 [OS=Homo sapiens]	PFN2	3	50	3	26	19.509	cell organization and biogenesis;regulation of biological process
O95336	6-phosphogluconolactonase [OS=Homo sapiens]	PGLS	6	68	6	34	25.338	metabolic process
Q96G03	Phosphoglucomutase-2 [OS=Homo sapiens]	PGM2	3	36	2	7	14.683	metabolic process;transport
Q6PCE3	glucose 1,6-bisphosphate synthase [OS=Homo sapiens]	PGM2L 1	6	51	6	10	18.964	metabolic process
O60664-1	Perilipin-3 [OS=Homo sapiens]	PLIN3	5	31	5	18	26.583	transport
Q02809	Procollagen-lysine,2-oxoglutarate 5-dioxygenase 1 [OS=Homo sapiens]	PLOD1	2	10	2	4	9.28	metabolic process;response to stimulus
P36873-1	serine/threonine-protein phosphatase PP1-gamma catalytic subunit [OS=Homo sapiens]	PPP1C C	4	35	2	18	20.548	cell differentiation;cell division;cell organization and biogenesis;metabolic process;regulation of biological process;response to stimulus
P62714	serine/threonine-protein phosphatase 2A catalytic subunit beta isoform [OS=Homo sapiens]	PPP2C B	4	36	4	18	19.018	cell organization and biogenesis;metabolic process;regulation of biological process;response to stimulus
P30153	serine/threonine-protein phosphatase 2A 65 kDa regulatory subunit A alpha isoform [OS=Homo sapiens]	PPP2R1 A	9	87	9	20	50.644	cell death;cell organization and biogenesis;metabolic process;regulation of biological process;response to stimulus
Q6P2Q9	Pre-mRNA-processing-splicing factor 8 [OS=Homo sapiens]	PRPF8	9	88	9	4	39.331	cell organization and biogenesis;metabolic process;response to stimulus
P25789	Proteasome subunit alpha type-4 [OS=Homo sapiens]	PSMA4	4	44	4	18	16.736	metabolic process;regulation of biological process;response to stimulus;transport

O14818-1	Proteasome subunit alpha type-7 [OS=Homo sapiens]	PSMA7	3	58	3	14	17.679	metabolic process;regulation of biological process;response to stimulus;transport
P17980	26S proteasome regulatory subunit 6A [OS=Homo sapiens]	PSMC3	10	102	10	28	41.944	metabolic process;regulation of biological process;response to stimulus;transport
O00231	26S proteasome non-ATPase regulatory subunit 11 [OS=Homo sapiens]	PSMD1 1	5	33	5	15	19.531	cell differentiation;cell organization and biogenesis;metabolic process;regulation of biological process;response to stimulus;transport
Q9UNM6	26S proteasome non-ATPase regulatory subunit 13 [OS=Homo sapiens]	PSMD1 3	4	34	4	9	11.47	cell organization and biogenesis;metabolic process;regulation of biological process;response to stimulus;transport
Q13200	26S proteasome non-ATPase regulatory subunit 2 [OS=Homo sapiens]	PSMD2	8	67	8	10	39.613	metabolic process;regulation of biological process;response to stimulus;transport
O43242	26S proteasome non-ATPase regulatory subunit 3 [OS=Homo sapiens]	PSMD3	7	70	7	16	27.884	metabolic process;regulation of biological process;response to stimulus;transport
P55036-1	26S proteasome non-ATPase regulatory subunit 4 [OS=Homo sapiens]	PSMD4	3	22	3	13	8.88	cell organization and biogenesis;metabolic process;regulation of biological process;response to stimulus;transport
Q16401	26S proteasome non-ATPase regulatory subunit 5 [OS=Homo sapiens]	PSMD5	2	15	2	5	6.049	cell organization and biogenesis;metabolic process;regulation of biological process;response to stimulus;transport
Q92530	proteasome inhibitor PI31 subunit [OS=Homo sapiens]	PSMF1	3	12	3	15	9.965	metabolic process;regulation of biological process;response to stimulus;transport
P47897	glutamine--tRNA ligase [OS=Homo sapiens]	QARS	7	60	7	12	25.47	metabolic process;regulation of biological process
P61026	ras-related protein rab-10 [OS=Homo sapiens]	RAB10	3	72	1	17	19.93	cell differentiation;cell organization and biogenesis;regulation of biological process;response to stimulus;transport

P51149	ras-related protein Rab-7a [OS=Homo sapiens]	RAB7A	4	81	4	22	28.085	cell organization and biogenesis;cellular homeostasis;metabolic process;regulation of biological process;response to stimulus;transport
Q9UKM9-1	RNA-binding protein Raly [OS=Homo sapiens]	RALY	3	46	3	9	12.838	metabolic process;regulation of biological process
P46060	Ran GTPase-activating protein 1 [OS=Homo sapiens]	RANGAP1	2	29	2	4	10.147	cell organization and biogenesis;metabolic process;regulation of biological process;response to stimulus
P61224-1	Ras-related protein Rap-1b [OS=Homo sapiens]	RAP1B	4	68	3	19	21.985	cell organization and biogenesis;cell proliferation;regulation of biological process;response to stimulus;transport
P61225	ras-related protein Rap-2b [OS=Homo sapiens]	RAP2B	2	41	2	13	10.78	cell organization and biogenesis;regulation of biological process;response to stimulus;transport
P46779	60S ribosomal protein L28 [OS=Homo sapiens]	RPL28	3	37	3	27	11.605	cell organization and biogenesis;metabolic process;regulation of biological process;transport
P39023	60S ribosomal protein L3 [OS=Homo sapiens]	RPL3	6	105	6	18	36.542	cell organization and biogenesis;metabolic process;response to stimulus;transport
P49207	60S ribosomal protein L34 [OS=Homo sapiens]	RPL34	2	59	2	13	10.187	cell organization and biogenesis;metabolic process;transport
P46777	60S ribosomal protein L5 [OS=Homo sapiens]	RPL5	7	150	7	29	44.343	cell organization and biogenesis;metabolic process;regulation of biological process;transport
P62917	60S ribosomal protein L8 [OS=Homo sapiens]	RPL8	5	71	5	22	25.443	cell organization and biogenesis;metabolic process;response to stimulus;transport
Q9NQG5	regulation of nuclear pre-mRNA domain-containing protein 1B [OS=Homo sapiens]	RPRD1B	3	36	3	14	13.411	metabolic process;regulation of biological process

P08708	40S ribosomal protein S17 [OS=Homo sapiens]	RPS17; RPS17L	3	27	3	40	10.947	cell organization and biogenesis;metabolic process;transport
P62847-1	40S ribosomal protein S24 [OS=Homo sapiens]	RPS24	4	63	4	35	22.684	cell organization and biogenesis;metabolic process;transport
O76021	Ribosomal L1 domain-containing protein 1 [OS=Homo sapiens]	RSL1D1	4	28	4	11	13.41	cell differentiation;metabolic process;regulation of biological process
O75396	Vesicle-trafficking protein SEC22b [OS=Homo sapiens]	SEC22B	3	38	3	21	17.174	cell organization and biogenesis;regulation of biological process;transport
Q15436	protein transport protein Sec23A [OS=Homo sapiens]	SEC23A	9	109	9	16	46.715	cell organization and biogenesis;transport
P61619	Protein transport protein Sec61 subunit alpha isoform 1 [OS=Homo sapiens]	SEC61A 1	3	39	3	5	14.945	cell growth;cell organization and biogenesis;response to stimulus;transport
Q15019	septin-2 [OS=Homo sapiens]	SEPT2	6	128	5	22	37.314	cell differentiation;cell division;cell organization and biogenesis;regulation of biological process;response to stimulus
Q9UHD8-1	Septin-9 [OS=Homo sapiens]	SEPT9	5	55	4	9	24.754	cell division;cell organization and biogenesis;regulation of biological process
Q15393-1	Splicing factor 3B subunit 3 [OS=Homo sapiens]	SF3B3	2	27	2	2	10.51	cell organization and biogenesis;metabolic process;regulation of biological process
Q9BWM7	Sideroflexin-3 [OS=Homo sapiens]	SFXN3	4	71	4	18	20.592	transport
Q6NUK1	Calcium-binding mitochondrial carrier protein SCaMC-1 [OS=Homo sapiens]	SLC25A 24	4	32	4	9	16.206	regulation of biological process;response to stimulus;transport
P12236	ADP/ATP translocase 3 [OS=Homo sapiens]	SLC25A 6	9	321	2	35	53.298	cell death;cell organization and biogenesis;regulation of biological process;response to stimulus;transport
Q13596	Sorting nexin-1 [OS=Homo sapiens]	SNX1	2	26	1	3	10.142	cell organization and biogenesis;metabolic process;regulation of biological process;transport

Q9UNH7-1	Sorting nexin-6 [OS=Homo sapiens]	SNX6	3	17	3	9	11.681	cell organization and biogenesis;regulation of biological process;transport
Q13813	Spectrin alpha chain, non-erythrocytic 1 [OS=Homo sapiens]	SPTAN1	7	44	7	4	32.09	cell organization and biogenesis;cellular component movement;metabolic process;regulation of biological process;response to stimulus;transport
Q9UHB9	Signal recognition particle subunit SRP68 [OS=Homo sapiens]	SRP68	3	63	3	6	15.869	cell organization and biogenesis;response to stimulus;transport
P53597	Succinate--CoA ligase [ADP/GDP-forming] subunit alpha, mitochondrial [OS=Homo sapiens]	SUCLG1	2	36	2	9	9.71	metabolic process
Q96I99-1	Succinate--CoA ligase [GDP-forming] subunit beta, mitochondrial [OS=Homo sapiens]	SUCLG2	3	22	3	11	14.595	metabolic process
Q9UH65	Switch-associated protein 70 [OS=Homo sapiens]	SWAP70	2	31	2	3	8.855	cell organization and biogenesis;cellular homeostasis;metabolic process;regulation of biological process
O60506	Heterogeneous nuclear ribonucleoprotein Q [OS=Homo sapiens]	SYNCRIP	8	101	5	15	31.724	cell differentiation;metabolic process;regulation of biological process;response to stimulus
Q86WV6	Stimulator of interferon genes protein [OS=Homo sapiens]	TMEM173	5	111	5	20	48.171	cell death;defense response;regulation of biological process;response to stimulus;transport
Q92973-1	transportin-1 [OS=Homo sapiens]	TNPO1	9	71	9	12	38.835	cellular component movement;regulation of biological process;transport
Q02880	DNA topoisomerase 2-beta [OS=Homo sapiens]	TOP2B	8	95	8	6	40.381	cell organization and biogenesis;cellular component movement;metabolic process;regulation of biological process

Q14258	E3 ubiquitin/ISG15 ligase TRIM25 [OS=Homo sapiens]	TRIM25	5	48	5	9	25.203	defense response;metabolic process;regulation of biological process;response to stimulus
Q9BQE3	Tubulin alpha-1C chain [OS=Homo sapiens]	TUBA1C	20	2147	3	62	253.702	cell division;cell organization and biogenesis;transport
P22695	Cytochrome b-c1 complex subunit 2, mitochondrial [OS=Homo sapiens]	UQCRC2	9	96	9	26	47.904	metabolic process;transport
P45974-1	Ubiquitin carboxyl-terminal hydrolase 5 [OS=Homo sapiens]	USP5	8	51	8	14	33.21	metabolic process;regulation of biological process
P26640	Valine--tRNA ligase [OS=Homo sapiens]	VARS; VARS2	6	24	6	7	20.586	metabolic process;regulation of biological process
O75436-1	Vacuolar protein sorting-associated protein 26A [OS=Homo sapiens]	VPS26A	2	15	2	9	7.735	regulation of biological process;response to stimulus;transport
P23381	Tryptophan--tRNA ligase, cytoplasmic [OS=Homo sapiens]	WARS	3	28	3	10	11.966	metabolic process;regulation of biological process
Q9UIA9	exportin-7 [OS=Homo sapiens]	XPO7	4	40	4	4	14.299	transport
P12956	X-ray repair cross-complementing protein 6 [OS=Homo sapiens]	XRCC6	15	173	15	31	70.545	cell organization and biogenesis;defense response;metabolic process;regulation of biological process;response to stimulus;transport
P31946	14-3-3 protein beta/alpha [OS=Homo sapiens]	YWHAB	5	163	2	27	21.855	cell organization and biogenesis;metabolic process;regulation of biological process;response to stimulus;transport
P63104-1	14-3-3 protein zeta/delta [OS=Homo sapiens]	YWHAZ	7	207	5	29	57.138	cell organization and biogenesis;metabolic process;regulation of biological process;response to stimulus;transport
